# Macrophage-rich niches regulate T cell dynamics at the liver invasive margin during gallbladder cancer progression

**DOI:** 10.1172/JCI193672

**Published:** 2026-03-02

**Authors:** Maolan Li, Zhaonan Liu, Shenbing Shan, Ziyao Jia, Yongsheng Li, Fatao Liu, Lina Lu, Shimei Qiu, Chen Li, Ziyi Wang, Siyuan Yan, Yuhao Zhao, Lili Gao, Zhiqing Yuan, Yuanding Liu, Jiyao Ma, Jiayi Feng, Pengxiao Geng, Yiming Li, Xiaojing Xu, Xinhua Lin, Changjun Liu, Zebing Liu, Wenguang Wu, Xiangsong Wu, Wei Gong, Yanjing Li, Dongxi Xiang, Yongning He, Yun Liu, Rong Shao, Kwan Man, Wu Wei, Yingbin Liu

**Affiliations:** 1 Department of Biliary-Pancreatic Surgery, Renji Hospital, Shanghai Jiao Tong University School of Medicine, Shanghai, China.; 2 Department of General Surgery, Jiading Center Hospital, Shanghai Jiao Tong University School of Medicine, Shanghai, China.; 3 Shanghai Key Laboratory of Systems Regulation and Clinical Translation for Cancer, Shanghai, China.; 4 Lingang Laboratory, Shanghai, China.; 5 State Key Laboratory of Systems Medicine for Cancer, Shanghai Cancer Institute, Renji Hospital, Shanghai Jiao Tong University School of Medicine, Shanghai, China.; 6 Network and Information Center, Shanghai Jiao Tong University, Shanghai, China.; 7 Department of Pathology, Xinhua Hospital, Shanghai Jiaotong University School of Medicine, Shanghai, China.; 8 Shanghai Center of Biomedicine Development and; 9 Department of Hepatobiliary Surgery, Hunan Provincial People’s Hospital (The First Affiliated Hospital of Hunan Normal University), Changsha, Hunan Province, China.; 10 Department of Pathology, Ren Ji Hospital, Shanghai Jiao Tong University School of Medicine, Shanghai, China.; 11 Laboratory of General Surgery and Department of General Surgery, Xinhua Hospital affiliated with Shanghai Jiao Tong University School of Medicine, Shanghai, China.; 12 Shanghai Key Laboratory of Biliary Tract Disease Research, Xinhua Hospital, Shanghai, China.; 13 Department of Surgery, Li Ka Shing Faculty of Medicine, The University of Hong Kong, Hong Kong, China.

**Keywords:** Immunology, Oncology, Bioinformatics, Cancer immunotherapy

## Abstract

Liver invasion is one of the most frequent events in the progression of gallbladder cancer (GBC). However, the cellular and pathological role of the tumor-liver–interface microenvironment in liver invasion is still enigmatic. Here, we applied single-cell and spatial transcriptomics to systematically investigate the cellular component and gene expression regulation of the microenvironment from the tumor to the liver, specifically the invasive boundary. Our analyses revealed that CXCL9^+^ macrophage–rich immune cell niches were accumulated in the tumor-liver invasive margin, where 2 subclasses of the CXCL9^+^ immune cell niches, CXCL9^+^TRAC^+^ (CT) and CXCL9^+^C1QB^+^ (CC) niches, were identified. CD8^+^ T cells were recruited by CXCL9^+^ macrophages through CXCL9-CXCR3 interaction in the CT niche, which was located adjacent to the liver. Moreover, the CC niche was proximal to the tumor core, where tumor cells induced CD8^+^ T cell exhaustion via LGALS4 expression. In addition, our cohort study showed that high CXCL9 and low LGALS4 in the liver invasion margin demonstrated a favorable prognosis and better responses to anti–PD-1 immunotherapy for patients with gallbladder cancer. Altogether, these findings demonstrate novel cellular and molecular mechanisms underlying liver invasion and offer clinical value for immunotherapies.

## Abstract 2

In gallbladder cancer, specific immune cells at the liver-tumor border influence T cell activity: high CXCL9 and low LGALS4 levels indicate better prognosis and immunotherapy response.

## Graphical abstract

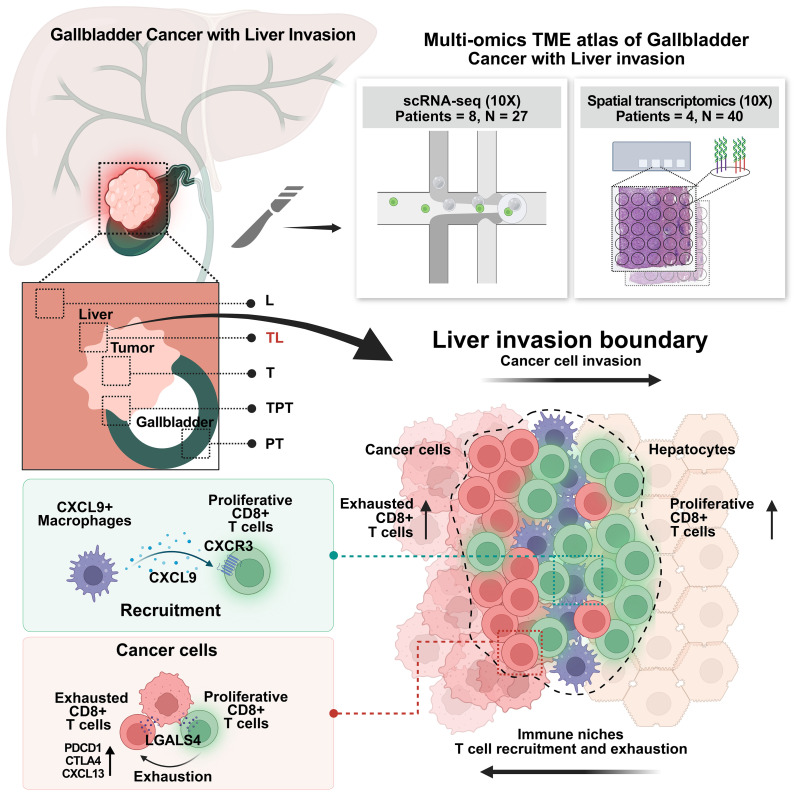

## Introduction

Gallbladder cancer (GBC) is an aggressive carcinoma with a 5-year survival rate of less than 20% (1–3). Strikingly, direct liver invasion is found in 40% of GBC cases due to anatomical adjacency between the gallbladder and liver (4, 5). Given the aggressive ability of GBC to invade the adjacent liver, intervention in this specific pathway has recently received considerable attention in clinical therapy. However, the mechanisms underlying GBC liver invasion remain to be fully understood.

The distinct structural specificities of the gallbladder and liver predispose the development of a highly heterogeneous microenvironment for tumor cell seeding and growth. To successfully invade the liver, GBC cells must overcome a variety of defensive challenges from varied cells located in the frontier niche, including hepatic immune cells and inflammation response cells (6). Therefore, it is of paramount importance to decipher immune cellular components and molecular mechanisms of their interaction with tumor cells in the tumor microenvironment (TME). Single-cell RNA sequencing (scRNA-seq) allowed us to identify various immune subtypes in GBC development, including tumor-associated macrophages, Tregs, and exhausted CD8^+^ T cells (7–9). However, the lack of spatial configuration for heterogeneous tumors fails to recapitulate individual cellular distributions within the TME. To locate these distinct subcellular types in tumors, spatial transcriptomics (ST) has been developed (10). Wu et al. discovered using ST that the invasion boundary of liver tumors harbored varied suppressive immune cells, including Tregs and M2 macrophages (11). This advanced approach has received significant attention in the study of carcinogenesis and cancer immunology.

In this study, we recruited 8 patients with GBC with direct liver invasion and employed an integrative ST approach combined with scRNA-seq. We identified M1-like CXCL9^+^ macrophages enriched in specialized immune cell niches that were orchestrated with 2 subclasses: CXCL9^+^C1QB^+^ (CC) niches and CXCL9^+^TRAC^+^ (CT) niches, showing distinct spatial distribution and cellular composition. In the CT niche located proximal to the liver, CD8^+^ T cells were recruited and stimulated into proliferation states via CXCL9-CXCR3 interaction through CXCL9^+^ macrophages. Meanwhile, in the CC niche proximal to the tumor core, LGALS4^+^ cancer cells induce CD8^+^ T cell exhaustion. The combination of CXCL9 and LGALS4 at the liver invasion boundary was correlated with prognosis and response to anti–PD-1 immunotherapy for patients with GBC. Our study unveiled spatially dynamic interactions among tumor cells, macrophages, and T cells in the immune cell niches during the direct liver invasion, offering mechanistic insights into the development of the complex TME.

## Results

### Multi-omics atlas of gallbladder cancer with direct liver invasion.

**Figure 1:**
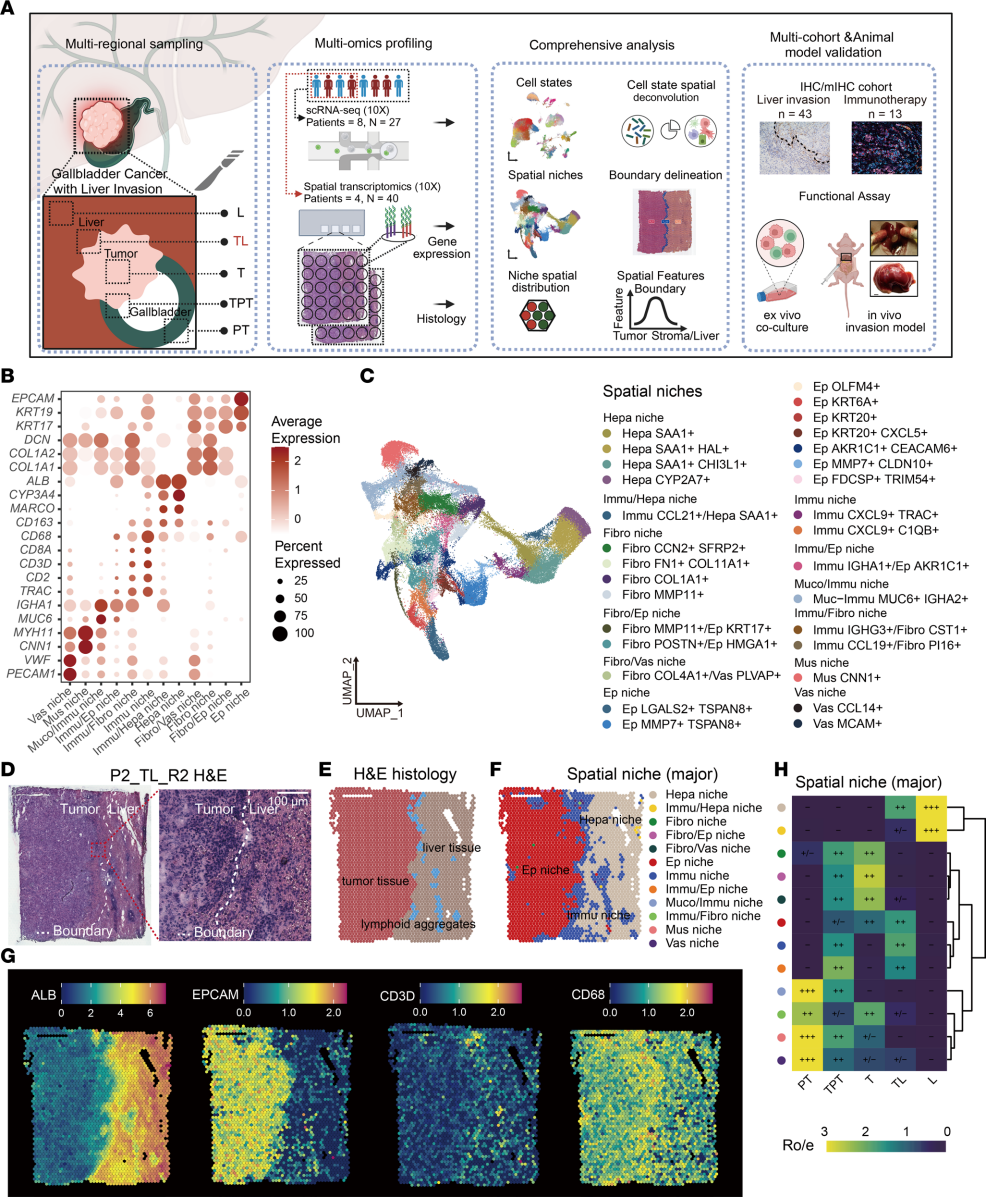
The spatial and single-cell landscape of GBC with direct liver invasion. (**A**) Schematic for the study workflow. PT, para-tumor gallbladder tissue; TPT, area of tumor invasive margin facing para-tumor gallbladder tissue; T, tumor core area; TL, tumor-liver invasive margin; L, distant normal liver. (Created with BioRender) (**B**) Bubble plot showing marker gene expression of 12 distinct major spatial niches. (**C**) UMAP representation of integrated spatial transcriptomics. (**D**–**G**) H&E staining (**D**), histology annotation (**E**), spatial niche mapping (**F**), and representative marker gene expression (**G**) in P2_TL_R2 slide. (**H**) Ro/e heatmap showing the relative abundance of major spatial niches across different sampling sites. See also Supplemental Figures 1–5.

To investigate the spatial heterogeneity of GBC liver invasion, we enrolled 8 cases of GBCs with direct liver invasion. We employed scRNA-seq and 10X Visium ST on tissue samples, including para-tumor gallbladder tissue (PT), area of tumor invasive margin facing PT (TPT), tumor core area (T), tumor-liver invasive margin (TL), and distant normal liver (L). A total of 27 samples from 8 patients with GBC were profiled by scRNA-seq, among whom 4 patients also contributed 40 samples for ST (Supplemental Table 1). Subsequent integrated analyses and validation strategies were summarized in the workflow diagram (Figure 1A).

scRNA-seq analysis obtained 140,213 high-quality cells with an average of 1,232 genes and 3,441 unique molecular identifiers (UMIs) per cell (Supplemental Figure 1A). According to canonical cell type markers, these cells were annotated into 8 main cell types and 1 cluster with a high proliferation feature (Supplemental Figure 1, B–E). T/NK cells dominated the most popular cells in TME (53.6%), followed by myeloid cells (15.6%) (Supplemental Figure 1, F and G). Furthermore, more T/NK cells were identified in the TL than in the T region (Supplemental Figure 1, H and I). To substantially define subtypes of these cells, we reclustered and reannotated our scRNA-seq data into 49 cell subtypes, including varied T cell subsets (Supplemental Figure 2, A–G, and Supplemental Table 2). Some of the identified cell substates were also reported elsewhere (Supplemental Figure 2, H–M) (12–14). These results indicate the immune cell heterogeneity within the TME of GBCs.

Next, 40 ST slides from 4 patients with GBC were employed to analyze. 70,207 spots with an average of 2,961 genes and 7,998 UMIs per spot were defined (Supplemental Figure 3A). All the spots were first integrated using robust principal component analysis (RPCA) and then classified into optimal numbers of niches according to the Fowlkes-Mallows Index (FMI). 12 major niches (Figure 1B, Supplemental Figure 3, B–F, and Supplemental Table 3), and subsequently 30 spatial subniches were designated based on their gene expression profiling (Figure 1C, Supplemental Figure 3G, and Supplemental Table 3). We also used scNiche (15), a state-of-the-art method to validate the niches we identified quantitatively (Supplemental Figure 3, H–K).

To precisely recapitulate these spatial niches in cancer samples, we compared the spatial niche with the histology classification annotated by 2 board-certified pathologists based on H&E staining (Figure 1, D–F). All the spots were annotated into 6 histological regions (Supplemental Figure 4, A–C). Of note, although a small portion, the lymphoid aggregates tissue increased in TL and L tissue compared with other sites (Supplemental Figure 4D). Correlation analysis demonstrated high consistency between spatial niches and histology classification (Supplemental Figure 4E). Gene expression patterns in the TL revealed exclusive distribution of *EPCAM* (tumor cell marker) and *ALB* (hepatocyte marker) to the tumor and liver, respectively (Figure 1G). *CD3D* (T cell marker) and *CD68* (myeloid cell marker) were predominantly expressed at the tumor-liver boundary in the TL region. To quantify the spatial patterns of niches shown in Supplemental Figure 5A, we employed Ro/e (the ratio of observed over expected cell numbers) analysis. The Immune (Immu) and Immune/Epithelial cell (Immu/Ep) niches with multiple myeloid and T cell marker expression were enriched in TL and TPT regions (Figure 1H and Supplemental Figure 5, B–D). In conclusion, the high-resolution atlas of GBC cell substates and spatial niches with scRNA-seq and ST established a novel platform reliable for the study of subcellular interaction, particularly for immune reaction during the direct liver invasion of GBC.

### CXCL9^+^ Immu niche is located at the tumor-liver invasion boundary.

**Figure 2:**
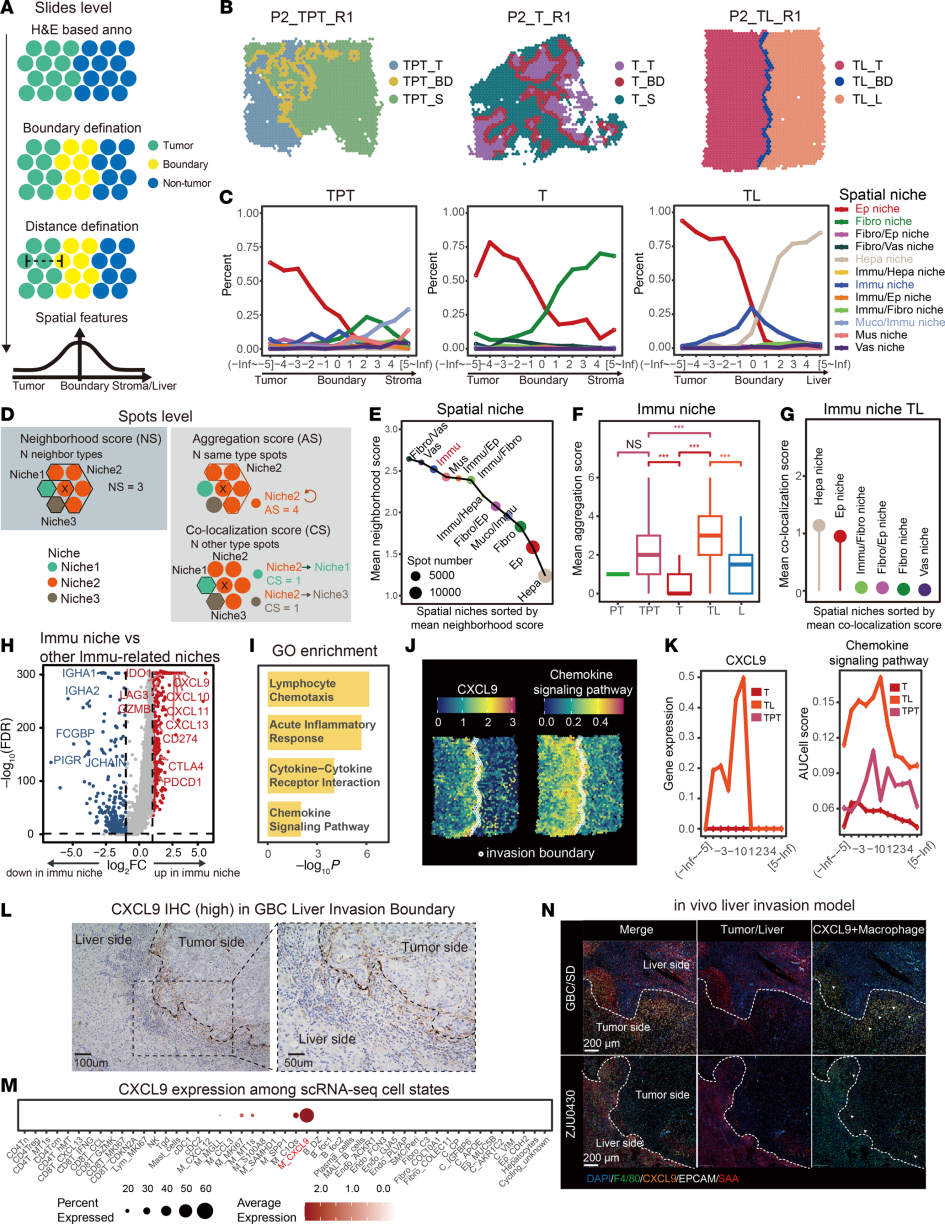
Spatial distribution and molecular characteristics of immune cell niches. (**A**) Scheme of invasion boundary (BD) and distance to BD analysis method in POSST pipeline. (**B**) BD was identified in different tumor regions (TPT, T, TL) from patient 2. (**C**) Dynamic changes of 12 spatial niches in different tumor regions (TPT, T, TL). Each layer was about 100 μm in width. (**D**) Schematic of neighborhood score (NS), aggregation score (AS), and colocalization score (CS) definition for center spot x in POSST pipeline. NS, AS, and CS reflect the regional diversity of proximate niches, self-clustering tendency, and abundance of specific interniche spatial association, respectively. (**E**) Line graph ordering of the mean NS from spatial niches. (**F**) Box plot showing AS of the immune cell niche (Immu niche) in 5 sampling sites. ***P < 0.001. (**G**) Lollipop graph displaying the mean CS between the Immu niche and other spatial niches in TL. (**H**) Volcano plot showing differentially expressed genes in the immune-cell–related (Immu-related) niches. (**I**) Bar plot revealing gene ontology analysis in Immu niches. (**J** and **K**) *CXCL9* expression (left) and AUCell score of the chemokine signaling pathway (right) in representative TL slides (**J**) and all samples (**K**). BD spots were labeled with hollow circles. (**L**) Representative CXCL9 IHC staining in a GBC tumor-liver invasion section. The left panel shows the entire interface; the right panel is a magnified view of the boxed area, highlighting the invasive boundary. Scale bars: 100 μm (left); 50 μm (right). (**M**) Bubble plot showing CXCL9 expression in distinct cell states from scRNA-seq. (**N**) Representative mIHC staining of tumor liver invasion tissue in the liver invasion model developed by GBC cell lines GBC/SD and ZJU0430 in nude mice. CXCL9^+^ macrophages were labeled with white arrows. See also Supplemental Figures 6 and 7.

To determine the molecular signature of the Immu niches characterized by myeloid and T cells in the tumor-liver invasion boundary, we developed position analysis of sequencing-based spatial transcriptomics (POSST). This R-based pipeline defines the tumor invasion boundary (BD) and evaluates the distance from each spatial spot to BD. As shown in Figure 2, A and B, and Supplemental Figure 6A, the boundary was located at the center, with left spots representing the tumor region (negative distance) and right spots for the stroma/liver region (positive distance). Along this trajectory, the Epithelial cell (Ep) niches decreased, while fibroblast (Fibro) and mucosal/immune cell (Muco/Immu) niches in TPT, Fibro niches in T, and hepatocyte (Hepa) niches in TL increased (Figure 2C). Notably, Immu niches rose to the peak at the liver invasion boundary (TL_BD).

To further characterize the spatial architecture of Immu niches in TL, we defined 3 metrics: neighborhood, aggregation, and colocalization scores with POSST, reflecting regional diversity of proximate niches, self-clustering tendency, and abundance of specific inter-niche spatial association, respectively (Figure 2D). The Immu niche displayed a high diversity of proximate niches (Figure 2E). It also showed the highest self-clustering tendency in TL with strong interaction with Hepa and Ep niches (Figure 2, F and G). Immu niches in TPT highly interacted with Ep niches (Supplemental Figure 7A). The data imply that immune cell niches in TL may exert critical immunopathological activity in tumor invasion.

Then, transcriptomic profiling of the Immu niches showed significantly elevated expression of multiple chemokines (*CXCL9*, *CXCL10*, *CXCL11*, *CXCL13*), effector molecules in immunity (*GZMB*), and immune checkpoint-related genes (*CD274*, *PDCD1*, *LAG3*, *CTLA4*) compared with other immune-related niches (Immu/Hepa, Immu/Ep, Immu/Fibro, Muco/Immu niches) without boundary-specific distribution (Figure 2H and Supplemental Table 4). Pathway enrichment analysis highlighted lymphocyte chemotaxis, inflammatory response, and cytokine and chemokine signaling pathways within Immu niches (Figure 2I and Supplemental Table 5). The top signature gene, *CXCL9,* in the Immu niche was identified (Figure 2H). *CXCL9* was accumulated in the boundary of TL sites where chemokine pathways were markedly activated (Figure 2, J and K). In the validation IHC cohort of 43 patients with GBC with liver invasion, CXCL9 expressed in TL_BD was higher than that in the tumor core (Figure 2L and Supplemental Figure 7, B and C). CXCL9*,* a chemokine expressed mainly in myeloid cells, induces CD8^+^ T cell chemotaxis (16). Our scRNA-seq data also confirmed that *CXCL9* was mainly expressed in macrophages, termed CXCL9^+^ macrophage (M_CXCL9) (Figure 2M), which is also reported elsewhere (17). To validate the association of M_CXCL9 with liver invasion, we established an in situ GBC liver invasion model in nude mice, which confirmed the accumulation of M_CXCL9 in TL (Figure 2N and Supplemental Figure 7D).

To further investigate whether the boundary-specific distribution of Immu niches is unique to GBC or also exists in other liver-associated tumors, we collected 61 Visium slides of primary or metastatic liver cancers, in which 16 liver boundary slides were found (Supplemental Figure 7E and Supplemental Table 6). Accordingly, these slides were annotated histologically and analyzed with POSST for boundary definition (Supplemental Figure 7F). Agreed with earlier results, the Immu niches were enriched in the liver-tumor boundary, and the typical enrichment pattern was observed in 10/16 slides (Supplemental Figure 7, G and H), implicating that the boundary-specific distribution of the Immu niche is a feature for partial liver cancer. Using murine cell lines of pancreatic and colorectal cancer (PAN02 and CT26), we established immunocompetent liver invasion mouse models and observed CXCL9^+^ macrophages accompanied by T cell infiltration at the liver invasion boundary. The mIHC assays showed that specific knockout or overexpression of CXCL9 in macrophages via adeno-associated virus serotype 9 (AAV9) vectors led to a respective decrease or increase of CD8^+^ T cells in this region, consistent with the known role of CXCL9 in T cell recruitment (Supplemental Figure 7I). In summary, immune cell niches expressing CXCL9 — which are enriched with chemotactic signals for immune cells — form prominently at the invasive boundary of the liver.

### Two Immu subniches are spatially colocalized to exert different T cell activity.

**Figure 3:**
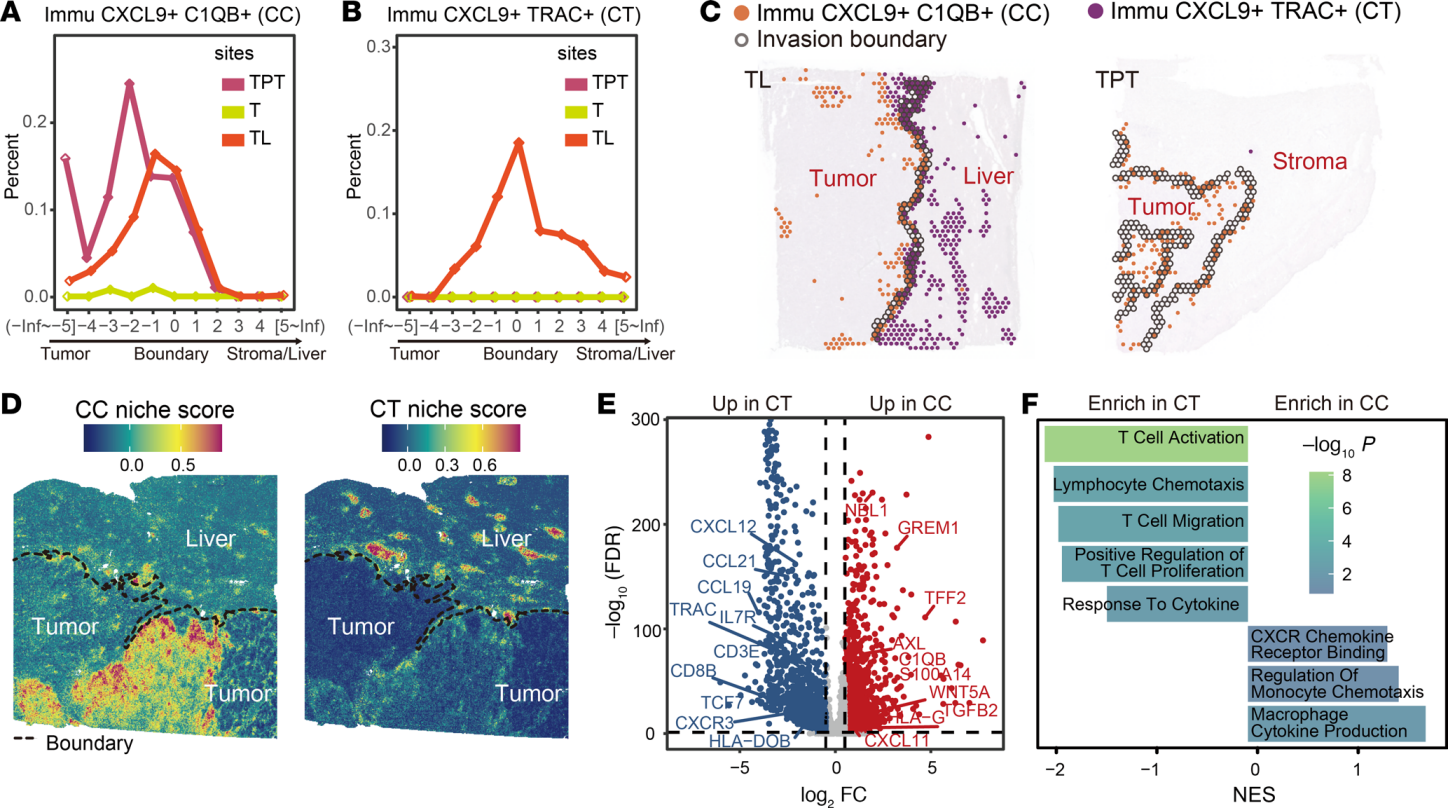
Spatial distribution and molecular characteristics of the Immu subniches. (**A** and **B**) Line graph showing the average fraction of the Immu CXCL9^+^C1QB^+^ (CC) and CXCL9^+^TRAC^+^ (CT) niches among different sampling tissues across spatial layers. Each layer was about 100 μm in width. (**C**) Spatial mapping showing the CC and CT niches’ location in TL and TPT. Invasion boundary spots were labeled with hollow circles. (**D**) Spatial distribution of CC and CT niche scores in the TL sample with Visium HD. The black line represents the liver-tumor boundary. (**E**) Volcano plot showing differential gene expression between CC and CT niches. (**F**) Bar plot showing enriched GO terms in the CC and CT niches with GSEA. See also Supplemental Figure 8.

To interrogate how T cells respond to M_CXCL9 in these immune cell niches, we analyzed their subclusters. Given the niche clustering in Figure 1C, the Immu niches included 2 subniches, Immu CXCL9^+^C1QB^+^ niches (CC niches) and Immu CXCL9^+^TRAC^+^ niches (CT niches), both of which were mainly distributed at the liver invasion boundary (Figure 3, A and B), while TPT sites only contained CC niches. Furthermore, within the TL region, more CC niches were accumulated on the tumor side; in contrast, CT niches were distributed mainly to the liver side (Figure 3C). To further validate their spatial distribution across the TL boundary, we quantified their top marker gene sets as CC and CT niche scores in an adjacent TL slide using Visium HD, providing a similar niche distribution pattern (Figure 3D). Other immune-associated niches, except TL-enriched niches (e.g., Immu IGHA1^+^/Ep AKR1C1^+^), didn’t show association with their location at the boundary region (Supplemental Figure 8, A and B). The codistribution of CC and CT niches in TL rather than TPT was further validated in Supplemental Figure 8, C–E. These results indicate that the CC and CT subniches robustly and specifically aggregated in the tumor-liver boundary.

To seek transcriptomic differences between CC and CT niches, we employed differential gene expression analysis. Lymphoid-related gene *CD3E*, naive/memory genes *IL7R*, *TCF7*, and T cell activation receptor *CXCR3* were upregulated in the CT niches, while myeloid-cell-associated genes (e.g., *C1QB*) were increased in CC niches (Figure 3E and Supplemental Table 7). Consistent with this, GSEA indicated T cell migration, proliferation, and activation in the CT niches, and more myeloid cells were recruited into the CC niches, where myeloid/T cell motility pathways were upregulated, including monocyte recruitment, macrophage cytokine production, and CXCR chemokine receptor binding pathways (Figure 3F). These results suggest that 2 distinct immune cell subniches exert different T cell functions.

### CXCL9^+^ macrophages were colocalized with MKI67^+^ CD8^+^ T cells and CXCL13^+^ CD8^+^ T cells in the liver invasion boundary.

To further characterize the interaction between macrophages and T cells in these niches, we utilized cell2location to determine each spot’s cellular composition with scRNA-seq (Supplemental Figure 9, A and B). As expected, T cells were prevalently distributed in the TL region relative to the T and TPT areas and were relatively enriched in the boundary zone of TL. Myeloid cells were also extensively distributed in the boundary of TL and showed colocalization with T/NK cells (Supplemental Figure 9, C and D).

**Figure 4:**
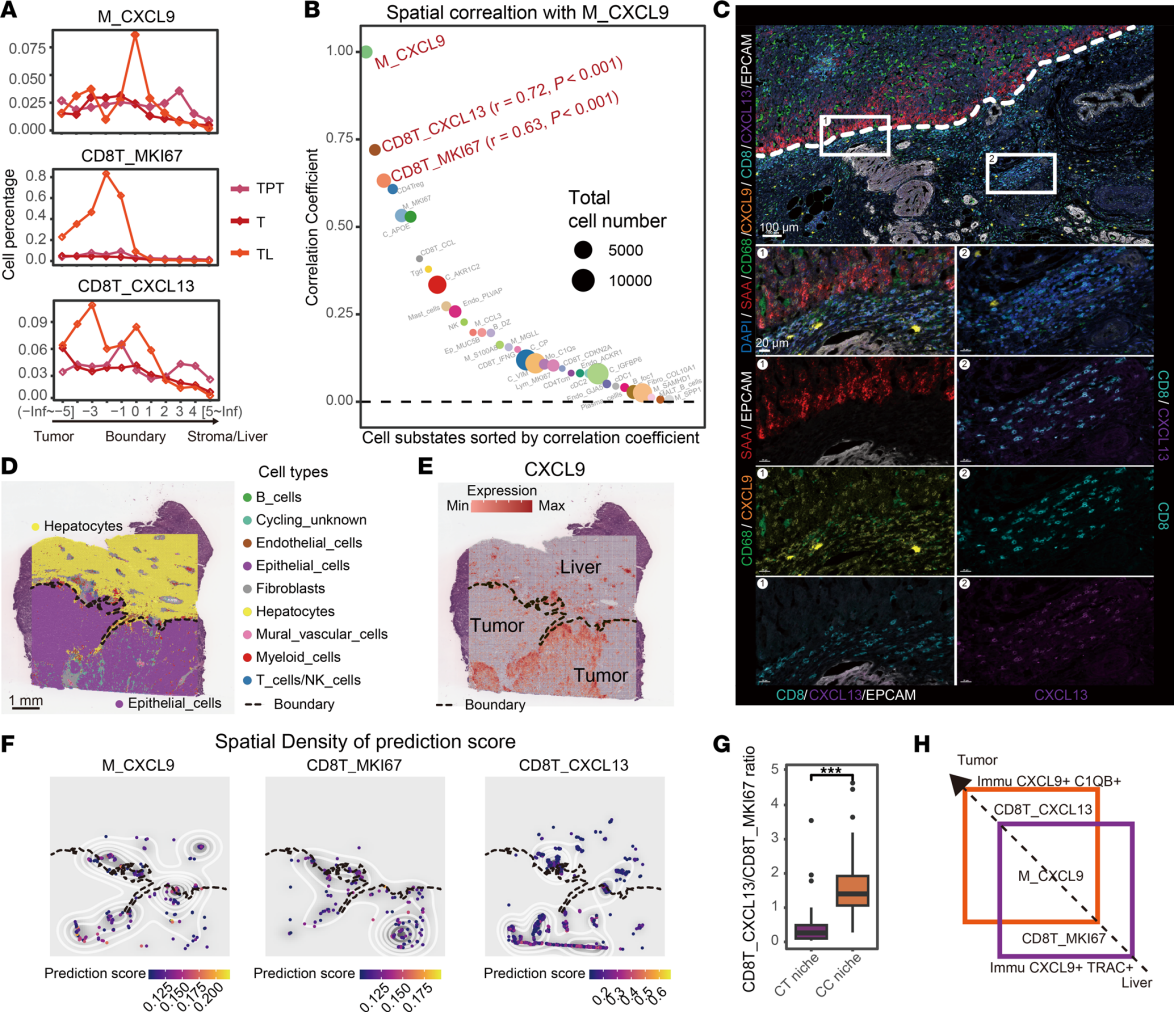
Cellular composition of the Immu subniches. (**A**) Average cell abundance of macrophages and T cells enriched in tumor-liver invasive margin (TL) in different spatial layers. (**B**) Ranking of the correlation coefficient between CXCL9^+^ macrophages (M_CXCL9) and other cell states in TL. Dot size represented the cell number of each cell state in TL. (**C**) Representative mIHC staining of M_CXCL9 and CXCL13^+^ CD8^+^ T cells (CD8T_CXCL13) in TL. Scale bar: 100 μm (upper) μm (lower). (**D**) Spatial plot of cell type distribution inferred by the FindTransferAnchors method in the TL slide using Visium HD. The black line represents the liver-tumor boundary. (**E**) Spatial gene expression profile detected with Visium HD. The black line represents the liver-tumor boundary. (**F**) Spatial density plot of cell substates distribution predicted by the FindTransferAnchors method. Dots indicate inferred cell substates, colored by prediction score. White contour lines depict prediction score density. Black line marks liver-tumor boundary. (**G**) Ratio of CD8T_CXCL13 versus proliferative MKI67^+^ CD8^+^ T cells (CD8T_MKI67) in CXCL9^+^C1QB^+^ (CC) and CXCL9^+^TRAC^+^ (CT) niches. (**H**) Schematic picture of M_CXCL9 and CD8^+^ T cell distribution in CC and CT niches. See also Supplemental Figure 9.

To identify cell subtypes of macrophages and T cells in TL, we utilized detailed annotations from scRNA-seq data. M_CXCL9 was the top population of myeloid cells enriched in TL_BD, where these cells displayed a center-peak distribution pattern (Figure 4A and Supplemental Figure 9E). Interestingly, proliferative MKI67^+^ CD8^+^ T cells (CD8T_MKI67) and CXCL13^+^ CD8^+^ T cells (CD8T_CXCL13) were also mainly distributed in the TL_BD. CD8T_MKI67 cells were accumulated on the tumor side proximal to the BD in TL, while CD8T_CXCL13 cells were highly distributed to both the BD and tumor side (Figure 4A). Further, it was shown that M_CXCL9 was spatially correlated with CD8T_CXCL13 and CD8T_MKI67 (Figure 4B). CXCL9^+^ tumor-associated macrophages are known to recruit CD8^+^ T cells to enhance antitumor immunity — a mechanism supported by numerous previous studies (18, 19). Their colocalization in the liver invasion boundary was further confirmed by mIHC staining (Figure 4C and Supplemental Figure 9F) and Visium HD spatial transcriptome analysis (Figure 4, D–F).

While M_CXCL9 cells were the most abundant macrophages for both niches, CD8T_CXCL13 and CD8T_MKI67 cells were distributed preferentially to the CC niche and the CT niche, respectively (Supplemental Figure 9G). Relative comparison analysis of CD8T_CXCL13 and CD8T_MKI67 displayed a significantly higher ratio in CC niches than in CT niches (*P* < 0.001) (Figure 4G). The spatial distribution of these T cells in the 2 niches exhibited slight overlapping at the boundary from the liver to the tumor (Figure 4H). Altogether, CD8T_CXCL13 and CD8T_MKI67 colocalized with M_CXCL9 are enriched in CC and CT niches at the liver invasion boundary.

### M1-like CXCL9^+^ macrophages are regulated by JAK-STAT signaling in the liver invasion boundary.

**Figure 5:**
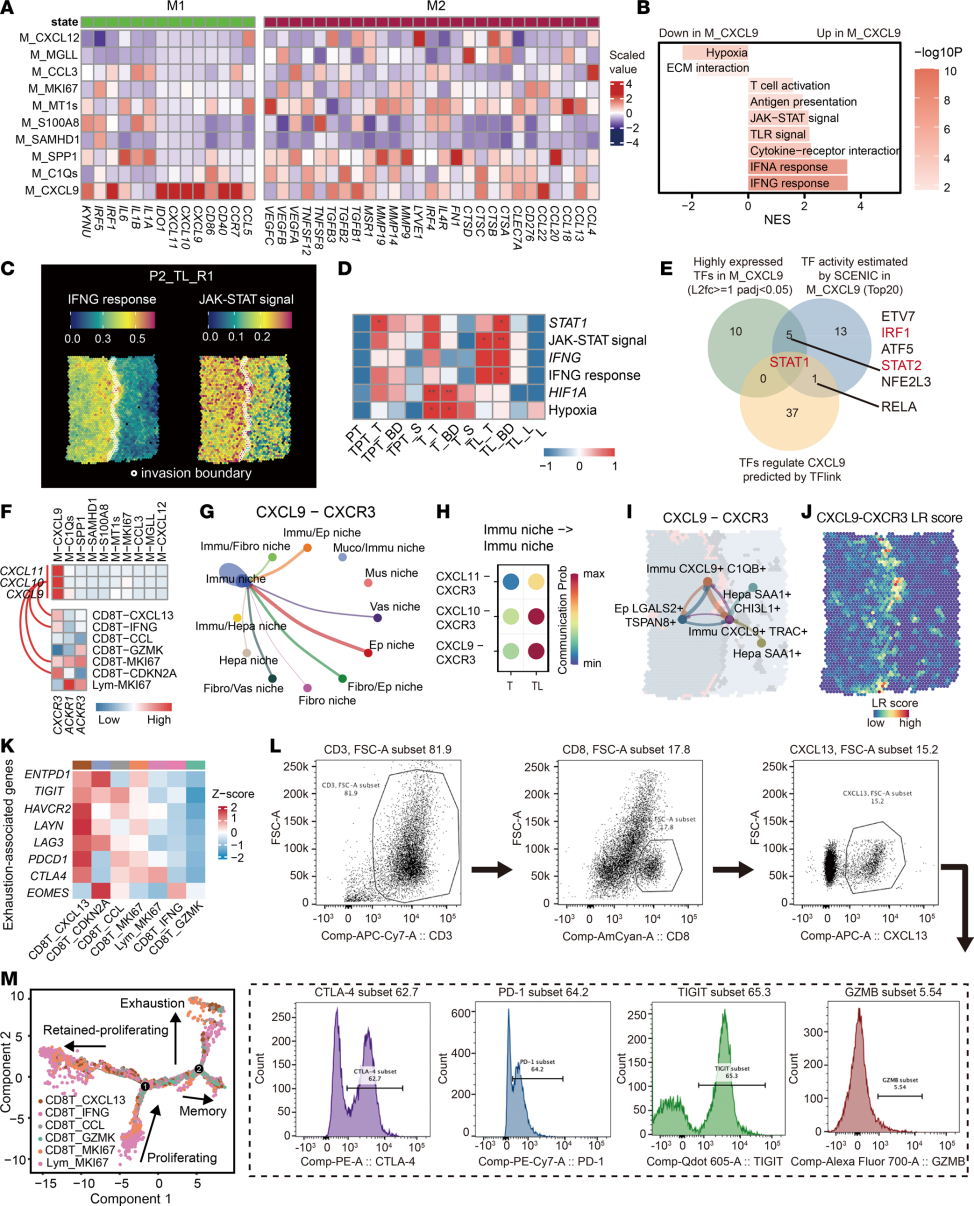
Single-cell characteristics of CXCL9^+^ macrophages and CD8^+^ T cells. (**A**) Scaled expression of representative markers of M1 and M2 states in macrophages. (**B**) Bar plot showing significantly enriched or deprived response in CXCL9^+^ macrophages (M_CXCL9) via GSEA. (**C**) Visualization of the AUCell score of the IFNG response and JAK-STAT signaling pathway in a representative tumor-liver invasive margin (TL) slide. Invasion boundary spots were labeled with hollow circles. (**D**) Heatmap showing scaled gene expression or AUCell scores of the gene sets in different subregions via spatial transcriptome. Asterisks indicate the increased events in a subregion compared with the other subregions. Blank, not significant; *P < 0.05; **P < 0.01. (**E**) Venn plot demonstrating overlapping of highly expressed transcription factors (TFs) in M_CXCL9 (Log_2_ fold change ≥ 1, adjusted *P* < 0.05), top 20 activated TFs inferred with SCENIC in M_CXCL9, and TFs regulating CXCL9 predicted with TFlinks. (**F**) Scaled expression of ligands and receptors among the macrophages and the CD8^+^ T cell states based on single-cell data. The red lines highlighted the CXCL9-CXCR3 ligand receptor (LR) pairs. (**G**) Cell interaction between niches via CXCL9-CXCR3 LR pairs. Line thickness represented the probability levels of communication. (**H**) Bubble plot comparing the communication probability of LR pairs within the Immu niche between TL and T sites. (**I**) In-situ cell-cell interaction of CXCL9-CXCR3 between spatial niches in a representative TL site. (**J**) CXCL9-CXCR3 interaction score in a representative TL slide. (**K**) Exhaustion-associated gene expression in CD8^+^ T cells presented in a trajectory. (**L**) Flow cytometry analyzing exhaustion phenotype of CXCL13^+^ CD8^+^ T cells. T cells were sorted from a healthy donor and cocultured with GBC cell lines in vitro for 48 hours. (**M**) Pseudotime trajectory of CD8^+^ T cells originating from MKI67^+^ lymphocytes (Lym_MKI67) and MKI67^+^ CD8^+^ T cells (CD8T_MKI67). See also Supplemental Figure 10.

Although the interaction between CD8^+^ T cells and macrophages has emerged to play a key role in tumor immunity, immune-specific cell subtypes and molecular mechanisms underlying the spatial interaction, particularly in the tumor-liver boundary, are poorly understood. M_CXCL9 lacked expression of markers for resident liver macrophages (*CD5L*, *CETP*, *TIMD4*), also known as Kupffer cells, suggesting that its primary origin is likely from peripheral blood monocytes (Supplemental Figure 10A). Interestingly, although most myeloid cells both expressed M1 and M2-associated genes, similar to previous single-cell studies (20), M_CXCL9 strongly expressed M1 markers, including *CXCL9*, *10*, *11*, and *IRF*, relative to weak expression of M2 markers (Figure 5A), indicating that M_CXCL9 cells act as M1-like macrophages. GSEA further revealed upregulation of IFN response, cytokine-receptor interaction, antigen presentation, and JAK-STAT signaling pathways in M_CXCL9, while hypoxia-related pathways were downregulated (Figure 5B). Consistent with scRNA-seq results, ST revealed that upregulation of IFNG-related pathways and JAK-STAT pathways in TL sites, especially in the boundary, while hypoxia-related pathways were mainly enriched in the tumor core (Figure 5, C and D). The results show the IFNG signaling in TL sites is activated to mediate macrophage infiltration and differentiation, as reported previously (21).

To further explore the upstream regulatory mechanisms of M_CXCL9 polarization, we sought to define transcription factors from different CXCL9-associated regulation datasets. The only identified potential TF candidate gene was *STAT1* (Figure 5E). *STAT1* was exclusively expressed and activated in M_CXCL9 (Supplemental Figure 10, B and C) and was the hub gene interacting with numerous genes involved in the JAK-STAT pathways (Supplemental Figure 10D). The constitutive and inducible STAT1 binding to the CXCL9 promoter is also reported in multiple studies on other conditions or diseases (22, 23). Except for these pathways, others upregulated in M_CXCL9, including antigen presentation and cytokine-receptor interaction, suggested that M1-like M_CXCL9 participates in the regulation of T cell chemotaxis and activation.

### CXCL9 macrophages recruit proliferative MKI67^+^ CD8^+^ T cells to infiltrate at the tumor-liver invasion boundary.

CXCL9 is a well-known chemokine able to recruit and activate T and NK cells by binding to CXCR3 (16, 24). NK cells defined by high *FCGR3A* and *FGFBP2* and low *CD3D* expression in our study show relatively low *CXCR3* expression compared with other T cell subsets (Supplemental Figure 10E). mIHC showed CXCR3 was expressed at a higher level in CD8^+^ T cells than in NK cells in GBC TL slides (Supplemental Figure 10F). Otherwise, NK cells showed no significant enrichment at the tumor-liver (TL) invasive margin (Supplemental Figure 2G). This low abundance and minimal CXCR3 expression suggest limited CXCL9-CXCR3–based chemotaxis between M_CXCL9 and conventional NK cells in our GBC model.

We subsequently hypothesized that M1-like M_CXCL9 recruits CD8^+^ T cells to the tumor-liver invasion boundary. To test this hypothesis, we examined the expression of CXCR3 and the interaction between CXCL9 and CXCR3. CXCR3 was highly expressed in several CD8^+^ T subtypes, including CD8T_MKI67 and CD8T_CXCL13, and the elevated CXCL9 in macrophages and corresponding CXCR3 in CD8T_MKI67 cells constituted the ligand-receptor interaction loop (Figure 5F). The CXCL9-CXCR3 interaction predominantly occurred in the Immu niche (Figure 5G), where M_CXCL9, CD8T_MKI67, and CD8T_CXCL13 mainly resided (Supplemental Figure 10G). Additionally, intercellular communication through the CXCL9-CXCR3 pair was stronger in TL compared with the T region (Figure 5H), particularly in CC and CT niches (Figure 5I). This event was also validated in the spatial based cell-cell communication analysis stLearn (Figure 5J). These results suggest that CXCL9 can recruit CD8^+^ T cells, especially proliferative CD8^+^ T cells, to infiltrate at the tumor liver invasion boundary.

### Proliferative MKI67^+^ CD8^+^ T cells differentiate into exhausted CXCL13^+^ CD8^+^ T cells in the liver invasion boundary.

To investigate different genetic signatures of CD8T_MKI67 and CD8T_CXCL13, we evaluated the gene expression profile, finding that CD8T_MKI67 cells expressed markers associated with proliferation (*MKI67*, *PCNA*, *STMN1*, *HELLS*) (Supplemental Figure 10H), whereas CD8T_CXCL13 expressed T cell exhaustion markers (*TIGIT*, *LAG3*, *PDCD1*, *CTLA4*) (Figure 5K). To validate the presence of exhausted CD8T_CXCL13, we utilized flow cytometry to analyze the exhaustion phenotype of CD8^+^ T cells cocultured with GBC cells. CD8T_CXCL13 exhibited high expression of PD-1, CTLA-4, and TIGIT, but low expression of GZMB, indicative of a highly exhausted and low cytotoxic phenotype (Figure 5L). We then used scRNA-seq to define the differentiation trajectory for CD8^+^ T cells. CD8T_MKI67 and Lym_MKI67, the initial differentiation cells, displayed varied abilities to develop distinct trajectories, giving rise to multiple subcellular populations, including retained proliferation, exhaustion, and memory T cells (Figure 5M and Supplemental Figure 10I). Notably, the exhausted state of CD8T_CXCL13 took place at the branch end of CD8T_MKI67 differentiation (Figure 5M), as the exhaustion-associated genes were gradually induced during the trajectory of cell differentiation (Supplemental Figure 10J). This finding was in line with our earlier results of the higher ratio of CD8T_CXCL13 to CD8T_MKI67 in CC niches than in CT niches (Figure 4G). The data suggest that CD8^+^ T cells undergo differentiation from proliferative to an exhausted state as they infiltrate into the tumor region from the invasion boundary. Collectively, our data indicate that M1-like M_CXCL9 and CD8T_MKI67 cells activate in TL proximal to the liver, and then these T cells undergo exhaustion differentiation into CD8T_CXCL13 in TL proximal to the tumor. This transition of the immune microenvironment in the TL region renders tumor cells invasive, leading to liver invasion.

### Tumor cells induce CD8^+^ T cell exhaustion dependent on LGALS4.

**Figure 6:**
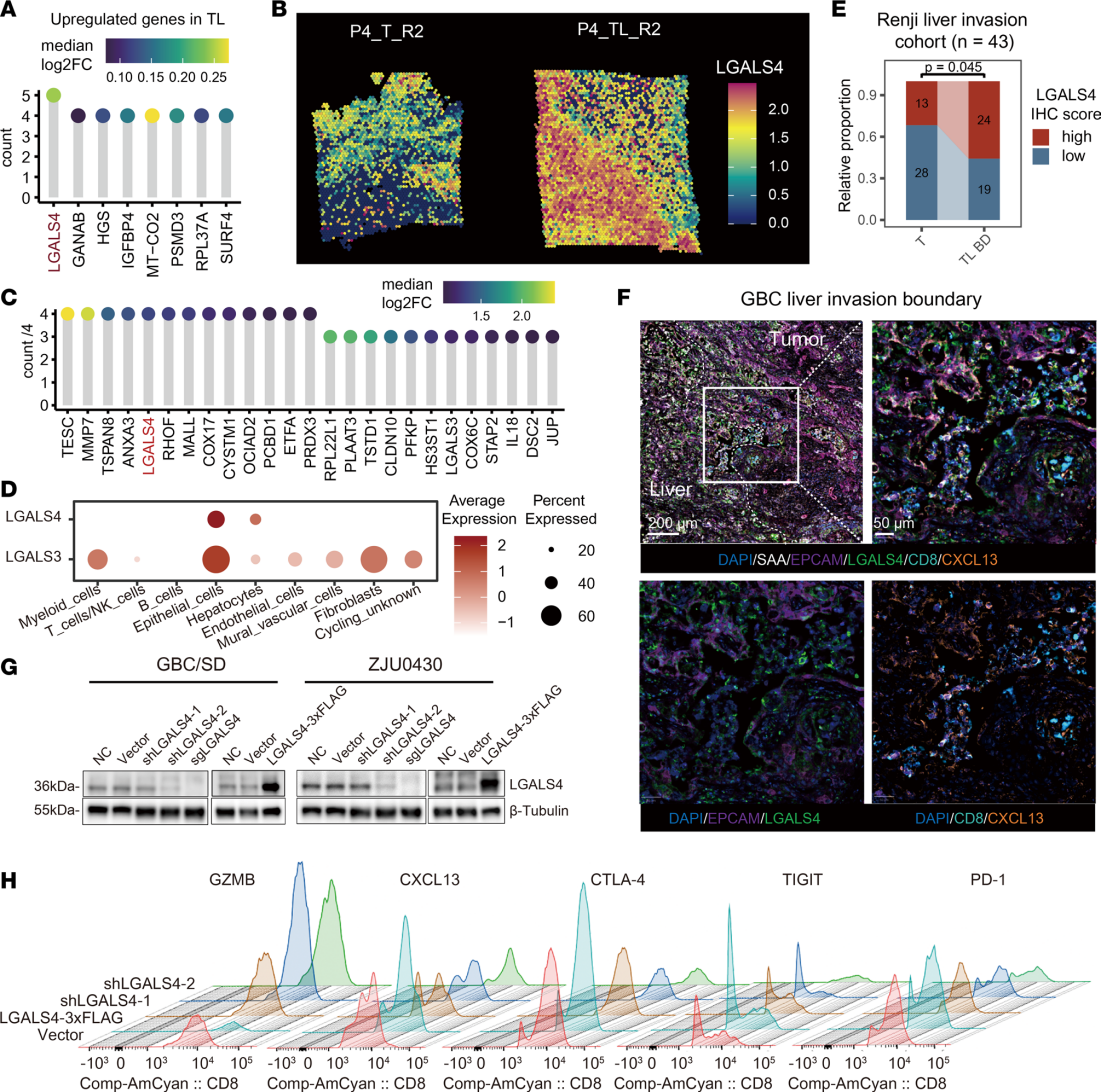
Tumor cells induce CD8^+^ T cell exhaustion dependent on LGALS4. (**A**) Lollipop chart revealing the shared upregulated genes among patients in tumor-liver invasive margin (TL) compared with tumor core area (T) in single-cell data. (**B**) Spatial mapping of *LGALS4* expression in T and TL sites using spatial data. (**C**) Lollipop chart showing the shared upregulated genes among patients in TL compared with T in spatial transcriptomics datasets. (**D**) LGALS4 and LGALS3 expression among different cell types. (**E**) The relative proportion of LGALS4 IHC score in the liver invasion boundary (TL_BD) and T in 43 patients with gallbladder cancer (GBC). (**F**) GBC liver invasion tissue with mIHC staining. Scale bar: 200 μm (upper left), 50 μm (upper right and lower). (**G**) Validation of LGALS4 knockdown, knockout, and overexpression in 2 GBC cell lines. (**H**) Flow cytometry analyzing the gene expression of T cells cocultured with GBC cell lines. See also Supplemental Figures 11 and 12.

The proliferative-to-exhausted state transition of CD8^+^ T cells occurs during their infiltration from the liver to the tumor. Thus, we hypothesized that cancer cells in the liver invasion boundary could induce T cell exhaustion in a certain way. We evaluated genetic alterations in T and TL regions using inferCNV with ST data, revealing amplification of chromosomes 8 and 17 with some of the corresponding oncogenes (*ERBB2*, *MYC*, and *SOX9*) in 3 of 4 patients with cancer (Supplemental Figure 11, A–C), which agreed with previous studies (8, 25, 26). Integrating ST and scRNA-seq datasets identified *LGALS4* as one of the genes upregulated in the highest number of TL sites compared with T tissues (5 of 8 in scRNA-seq, 4 of 4 in ST) (Figure 6, A–C, and Supplemental Figure 12A), and primarily expressed by epithelial cells (Figure 6D). IHC staining in patient samples validated LGALS4 expression in tumor cells at the TL boundary (Figures 6E and Supplemental Figure 12, B and C). These results suggest that LGALS4 upregulation in the liver-invasion clone may mediate tumor cell invasion.

Previous studies have reported that the GAL3 (encoded by LGALS3), another member of the galectin family secreted by tumor cells or macrophages, can induce T cell exhaustion by binding to LAG3 on the surface of T cells (27). Therefore, we investigated whether LGALS4 is a key factor in regulating T cell exhaustion. First, mIHC indicated a spatial colocalization of LGALS4 and CXCL13^+^ exhausted T cells (Figure 6F). To investigate the mechanisms for T cell exhaustion, we further constructed knockdown and overexpression stable cell lines of LGALS4 in 2 GBC cell lines, GBC/SD and ZJU0430 (Figure 6G). These cell lines were cocultured with human primary CD3^+^ T cells. Direct mixed coculture experiments between T cells and tumor cell lines, followed by flow cytometric analysis, showed that LGALS4 overexpression in GBC cells led to induction of CD8^+^ T cell exhaustion, as CXCL13, PD-1, and CTLA-4 expression was increased, but IFN-γ, GZMB, and TNF-α were decreased (Figure 6H and Supplemental Figure 12D). In contrast, knockdown or knockout of LGALS4 increased expression of T cell cytotoxic protein markers and decreased exhaustion markers (Supplemental Figure 12E). Therefore, our data suggest that LGALS4 expressed by tumor cells induces CD8^+^ T cell exhaustion.

### Expression levels of CXCL9 and LGALS4 in the liver invasion boundary are biomarkers for prognosis and immunotherapy responses in GBC.

**Figure 7:**
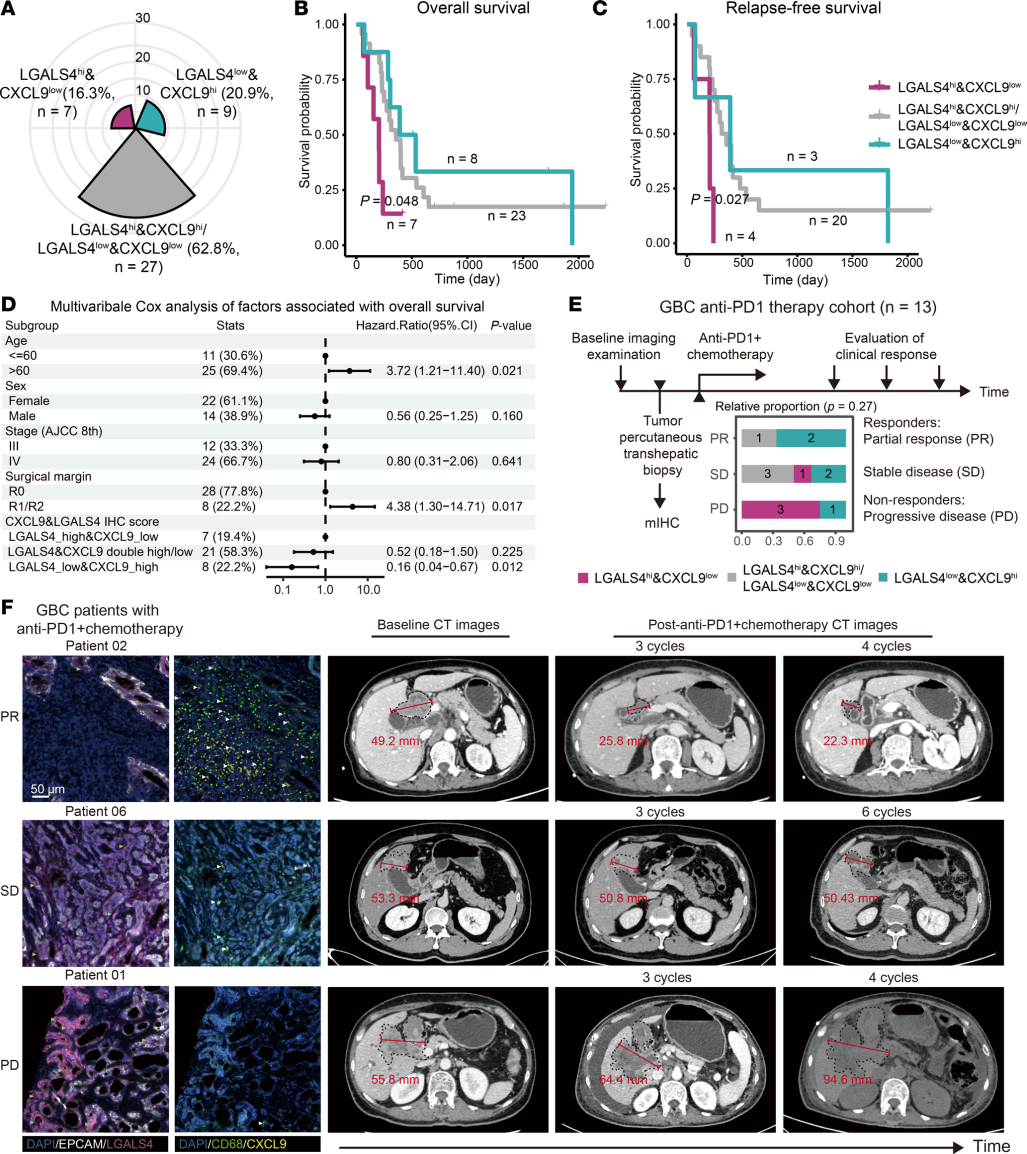
The combination of CXCL9 and LGALS4 in liver invasion tissue predicted prognosis in patients with GBC patients. (**A**) Pie plot showing the CXCL9&LGALS4 expression in GBC liver invasion cohort (*n* = 43). (**B** and **C**) The Kaplan-Meier overall survival (**B**) and relapse-free survival (**C**) curves of GBC liver invasion patients stratified by CXCL9&LGALS4 IHC scores in TL tissues. (**D**) Forrest plot showing multivariate COX analysis of overall survival for cohort in **A**. (**E**) Diagram of the GBC immunotherapy cohort and relative proportion of CXCL9&LGALS4 group in patients with distinct responses to the therapy. (**F**) Representative mIHC staining in tumor tissue of cohort in **E**, and their corresponding baseline and post-treatment CT images illustrating the maximum tumor diameters. Scale bar: 50 μm PR: partial response; SD: stable disease; PD: progressive disease. See also Supplemental Figure 13.

Our analysis revealed that macrophage-derived CXCL9 and tumor cell–derived LGALS4 are key mediators of CD8^+^ T cell recruitment and exhaustion at the liver invasion boundary of GBC. To validate the potential prognostic value of CXCL9 and LGALS4, we detected their expression in a GBC liver invasion cohort through IHC (*n* = 43). Stratification was based on the median expression score. Among the liver invasion area, 7 (16.3%) cases showed LGALS4^hi^&CXCL9^lo^, 9 (20.9%) cancers showed LGALS4^lo^&CXCL9^hi^, and the remaining 27 (62.8%) cases were double low or high (Figure 7A). Other clinicopathological information was summarized in Supplemental Table 8. Patients with LGALS4^lo^&CXCL9^hi^ in TL exhibited better overall survival (OS, *P* = 0.048, *n* = 38) and relapse-free survival (RFS, *P* = 0.027, *n* = 27), while LGALS4^hi^&CXCL9^lo^ demonstrated poor OS and RFS (Figure 7, B and C). Multivariable Cox regression analysis adjusted key prognostic factors and confirmed the significant association between CXCL9&LGALS4 staining and OS (LGALS4^lo^&CXCL9^hi^ versus LGALS4^hi^&CXCL9^lo^, HR = 0.16, 95% CI 0.04-0.67, *P* = 0.012) (Figure 7D). However, CXCL9&LGALS4 expression in the tumor core showed no significant association with OS or RFS (Supplemental Figure 13, A and B). Furthermore, retrospective evaluation of patients with GBC treated with anti–PD-1 (Toripalimab) and chemotherapy (Gemcitabine plus Cisplatin) postbiopsy (*n* = 13) revealed that 66.7% of partial responders (PR) were LGALS4^lo^&CXCL9^hi^, while 75% of patients with progressive disease (PD) were LGALS4^hi^&CXCL9^lo^ (*P* = 0.27) (Figure 7E, Supplemental Table 9). Notably, the tumor samples were collected through percutaneous transhepatic biopsy, representing the liver invasive regions. Changes in tumor volume (quantified by maximum tumor diameter) after 4 treatment cycles correlated positively with LGALS4^+^ tumor cells (*r* = 0.333, *P* = 0.266) and negatively with M_CXCL9 (*r* = –0.535, *P* = 0.06) (Figure 7F and Supplemental Figure 13, C and D). We further validated the predictive value of CXCL9 and LGALS4 for immunotherapy response in pancancer using the CIDE database (28), which revealed a protective association (negative prioritization score) for CXCL9 and a positive score for LGALS4, consistent with our findings (Supplemental Figure 13E). These findings suggest that the expression pattern of CXCL9 and LGALS4 in the liver invasion boundary is indicative of prognosis and immunotherapy response in patients with GBC.

## Discussion

Direct liver invasion is one of the most frequent events in GBCs. However, the mechanistic insights into the pathological invasion are still poorly understood. In this study, we combined ST and scRNA-seq to discover the CXCL9^+^ immune cell niche enriched in the tumor-liver boundary. Within the immune cell niche, the CD8^+^ T cells’ spatial dynamics from proliferation to exhaustion were driven by their multicellular interaction with CXCL9^+^ macrophage and LGALS4^+^ tumor cells.

We developed POSST, an R-based pipeline to (a) quantify the continuous alteration of the biological features from the tumor side, across the boundary, to the liver/stroma side; (b) evaluate intraniche and interniche distribution and their interaction spatially. In addition, spatial cellular patterns were engaged to construct the entire molecular, cellular, and geographical landscape of GBC liver invasion and reveal the dynamic configuration of individual niches, crossover tumor, boundary, and stroma/liver. Of interest, we also found the immune cell niche at the liver-tumor interface in a small population of primary and metastatic liver cancers, suggesting that these CXCL9^+^ niches may be common in liver cancers. However, its universality remains to be studied in more samples.

Previous studies have shown that the tumor invasion boundary exhibits a complex microenvironment, mainly composed of immunosuppressive cells like M2 macrophages, Treg cells, CAFs, and TANs (6–9, 29). Additionally, another study has indicated the enrichment of CD8^+^ T cells infiltrating the liver invasion boundary of GBC (30). Of note, we have comprehensively profiled the microenvironment of the liver invasion boundary and identified M1-like CXCL9^+^ macrophages able to recruit proliferative CD8^+^ T cells to the leading edge of tumor invasion, indicating a defense mechanism of the tumor immune microenvironment during liver invasion. CXCL9, expressed by myeloid cells, has been reported in numerous studies to recruit and activate T cells and is associated with the efficacy of immunotherapy (16, 31). While CXCL9^+^ macrophages recruited a significant number of CD8^+^ T cells to the liver invasion area, tumor cells built an “exhaustion barrier” at the edge. Our data confirmed that high LGALS4 expression in tumor cells led to significant CD8^+^ T cell exhaustion, providing insight that LGALS4^+^ tumor cells can undermine the antitumor function of recruited CD8^+^ T cells, thus permitting further tumor progression at the tumor frontier. However, due to the lack of murine gallbladder cancer cell lines, it was not feasible to employ HLA-matched T cells in coculture assays, which represents a limitation that may confound the interpretation of T cell–tumor interactions. The molecular mechanism underlying tumor cell–mediated T cell exhaustion through LGALS4 needs further exploration with a better model.

The approval of immune checkpoint blockade therapy combined with chemotherapy (gemcitabine and cisplatin) for advanced biliary tract tumors, as evidenced by the TOPAZ-1 trial, marks a significant advance (32). In the context of GBC with liver invasion, where radical surgery remains the standard of care, the neoadjuvant approach combining chemotherapy and immunotherapy, currently under clinical trials (NCT05640791, NCT04308174, NCT05451290, NCT06017297), holds promise. Our study identified that CXCL9 and LGALS4 were indicators of T cell recruitment and exhaustion, respectively. Interestingly, their combined expression profile at the liver invasion boundary, rather than the tumor core, was a prognostic and predictive biomarker for immunotherapy response. Although our sample size is limited by the rarity of GBC, our findings offer a perspective that not only the level of gene expression but also its spatial distribution pattern would be vital to the cellular function and clinical significance. Future work should prioritize collaborations of multiple centers to overcome the limitation of sample size.

In summary, our study has integrated multiple advanced technologies of ST and scRNA-seq with comprehensive data analysis models, unveiling an immune cell–rich TME located at the liver invasion boundary in GBC. Identifying the unique molecular and cellular aspects of CXCL9+ macrophage niches with individual proliferating and exhausted CD8+ T cells will likely be beneficial to improve efficacy prediction of immunotherapies.

## Methods

### Sex as a biological variable

Since no evidence shows sex differences in gallbladder cancer liver invasion, our study exclusively examined male mice. It is unknown whether the findings are relevant for female mice.

### Patients and tissue samples

Gallbladder cancer patients’ tissue and the peripheral blood of healthy donors were obtained from Renji Hospital, Affiliated with Shanghai Jiao Tong University School of Medicine. All patients’ diagnoses were pathologically confirmed. Clinical information of patients’ samples with the single-cell and spatial sequencing is summarized in Supplemental Table 1. Retrospective cohorts’ patient information was summarized in Supplemental Tables 8 and 9.

### Single-cell isolation of tissue

Tumor tissues for spatial transcriptome assays were collected. For single-cell isolation, the samples were processed as follows: minced, dissociated with digestant (0.25% trypsin [Thermo Fisher]), and 10 μg/mL DNase I (Sigma) dissolved in PBS with 5% FBS (Thermo Fisher), and incubated at 37°C with a shaking speed of 50 rpm for about 40 minutes. Cell suspensions were filtered using a 40-μm nylon cell strainer, and red blood cells were removed using 1X Red Blood Cell Lysis Solution (Thermo Fisher). Dissociated cells were washed with 1x DPBS containing 2% FBS. Cells were stained with 0.4% Trypan blue (Thermo Fisher) to check the viability on Countess II Automated Cell Counter (Thermo Fisher). The RNA from single cells was barcoded using 10X Genomics Chromium Single Cell 3′ Library and Gel Bead Kit and processed on a Chromium Single Cell Processor. Library construction followed the manufacturer’s instructions (10X Genomics), and sequencing was performed on a NovaSeq 6000 sequencing system (Illumina).

### scRNA-seq data processing

For sample sequences, the Cell Ranger software (v.7.1.0) was downloaded from 10x Genomics (https://support.10xgenomics.com/single-cell-gene-expression/software/downloads/latest) and used to process raw data, filter low-quality data, align reads to the hg38 human reference genome, and summarize unique molecular identifier (UMI) counts. Reads mapped to intros were not counted as gene expression. Seurat (version 4.0.1) (33) was used for most single-cell data downstream analyses. Before the raw count matrix was loaded into Seurat, genes detected in fewer than 3 cells and cells with fewer than 200 genes were removed. Doublets were removed by DoubletFinder (version 2.0.3) (34). Furthermore, we calculated the metrics of each cell, and cells with more than 15% mitochondrial gene counts, fewer than 500 genes, or more than 5,000 UMIs were removed. After all single samples were merged into one Seurat object, the SCTransform function was applied to the data with the regress variables “percent.mt,” “S.Score,” and “G2M.Score.” The dataset was analyzed for the highly variable genes (HVGs), and the top 50 principal components were used in downstream analysis. Harmony (version 1.2.0) (35) was applied to remove patient batch effects. The top 30 components from Harmony were selected to execute the RunUMAP function in Seurat, generating a non-linear dimension reduction projection using Uniform Manifold Approximation and Projection (UMAP) or t-distributed Stochastic Neighbor Embedding (t-SNE) for visualization. A K nearest neighbor (KNN) graph was generated by FindNeighbors using the top 30 Harmony components, and clusters were classified by the FindClusters function with a manually selected resolution from 0.1 to 1.5. To obtain better annotation results, we processed 2 rounds of cell annotation. For the first round of annotation, a resolution of 0.5 was chosen to classify clusters, and canonical cell markers were used to annotate cell clusters into myeloid cells, T/NK cells, B cells, Epithelial cells, and other stromal cells. The first round of annotation gave rise to the main cell types. Then, a second round of annotation was run based on each major cell type, starting from the filtered cell expression count matrix. Notably, the second round of annotation was based on the signature genes of each cluster.

### Spatial transcriptome assay: Visium V1 assay

#### Sample preparation and sequencing.

Frozen tissue samples were embedded in OCT (Tissue-Tek) and cryosectioned. The 10-μm section was placed on the prechilled Optimization slides (Visium, 10X Genomics), and the optimal lysis time was determined. The tissues were treated as recommended by 10X Genomics, and the optimization procedure showed an optimal permeabilization time of 12 or 18 minutes of digestion and release of RNA from the tissue samples. Spatial gene expression slides (Visium V1, 10X Genomics) were used for spatial transcriptomics following the Visium User Guides. Next-generation sequencing libraries were prepared according to the Visium user guide. NovaSeq 6000 System (Illumina) was used for loading (300 pM) and sequencing libraries with a NovaSeq S4 Reagent Kit.

#### H&E staining tissue annotation.

The Visium V1 Spatial Gene Expression Slide cryosection was fixed with methanol, and the H&E Staining was performed. Brightfield histological images were scanned. Two board-certified oncology pathologists annotate all Visium spots into different histology areas and further classify them as “tumor” or “nontumor” using whole-slide H&E scans, without access to transcriptomic data with Loupe Browser 7.

### Data analysis

#### Data preprocessing.

For Visium V1 spatial transcriptome data, the SpaceRanger software (v2.1.0) was used to preprocess the sequencing data. hg38 was used as the reference genome for human data alignment, for tissues that shared the same Visium slides, manual image fiducial alignment and tissue detection were performed using Loupe Browser 7. Filtered feature-barcode expression matrices were used as initial input for the spatial transcriptomics analysis using Seurat (v4.0.1). Spots with less than 500 UMIs or over 20% mitochondrial gene percentage were filtered out. Raw count matrices were log-normalized and scaled for analysis.

#### Multisample integration.

For each patient’s replicate treated as a batch, we selected the top 2,000 highly variable genes (HVGs), and only those consistently variable across patients were retained for integration (Seurat:SelectIntegrationFeatures). Principal component analysis (PCA) was then applied to this shared feature space. To mitigate patient-specific batch effects, we performed integration using reciprocal PCA (RPCA). Integration anchors were identified across batches using the top 30 principal components and a conservative k.anchor parameter of 5 to prioritize robust alignments (Seurat:FindIntegrationAnchors). The datasets were integrated with the IntegrateData function.

#### Clustering optimization.

Integrated datasets were scaled, reduced to 30 principal components (PCs), and projected into UMAP space. A shared nearest-neighbor graph was constructed with FindNeighbors and clustered using FindNeighbors. We evaluated clustering robustness across resolutions (0.1–2.0) using the Fowlkes-Mallows Index (FMI). FMI remained > 0.7 (indicating high stability), peaking at 0.87 for resolution 1.2 (optimal cluster separation) (Supplemental Figure 3F).

#### Spatial niche robustness validation.

To further validate niche definitions, we applied scNiche, a method that integrates gene expression and spatial cell composition (via cell2location deconvolution). Following the developer’s pipeline, we: (a) performed scVI-based batch integration (aligned with our Seurat RPCA approach); (b) clustered spots using scNiche’s joint representation model; and (c) determined the optimal cluster number (k = 17) via FMI (Supplemental Figure 3H). The niches from scNiche showed high concordance with our original spatial niches, with a high median Adjusted Rand Index (ARI) of 0.67 (major niches) and 0.65 (minor niches) at tumor-liver interface regions. Niche 8 from scNiche, corresponding to the Immu niche, showed similar marker genes and spatial distribution (Supplemental Figure 3, I–K).

### Spatial transcriptome assay: Visium HD assay

For the Visium HD slide of the adjacent liver invasion tissue, we trimmed the tissue block by removing an approximately 200 μm layer to eliminate potential artifacts from previous spatial transcriptomic runs, ensuring tissue integrity. RNA quality assessment of the trimmed section confirmed its high quality (RIN = 8.5, Agilent 2100 Bioanalyzer). Following the Visium HD Fresh Frozen Tissue Preparation Handbook (CG000763), we processed the sample with H&E staining and imaging, then performed sequencing following the Visium HD Spatial Gene Expression Reagent Kits User Guide (CG000685) with Visium Human Transcriptome Probe Kit v2. Spatial and gene expression matrices were generated with Space Ranger 4.0.1 (level bin16, approximately single cell, for downstream analysis). H&E images were annotated by a board-certified pathologist using Loupe Browser 9. The precise boundary separating the tumor from the liver side was labeled. To resolve cell-type composition, we integrated single-cell RNA-seq data as a reference. We applied Seurat’s FindTransferAnchors function for label transfer, enabling quantitative mapping of cell-type distributions across tissue regions.

#### Tissue preference of spatial niches and cell states estimated by Ro/e.

We used the Ro/e value to estimate the tissue preference of spatial niche and cell states, as previously described (36). Ro/e represented the ratio of observed-to-expected spot numbers in a niche. The expected spot numbers of each niche cluster in each tissue or subregion were obtained from the χ^2^ test. Ro/e >1 represented the niche enriched in that region. The R package STARTRAC was used to calculate the Ro/e value.

### POSST (position analysis of sequencing-based spatial transcriptomics)

An open R tool, POSST, was developed to delineate the tumor invasion boundary and quantify the niche spatial distribution. The R package was uploaded to GitHub, which can be installed with “devtools:install_github(“zaozaozaonan/POSST”) (commit ID 753373d6f1bfcd19462c889ec1f42cdae1d8209e).

#### Tumor invasion boundary definition and distance to the boundary.

POSST employs a quantitative system developed in collaboration with clinical pathologists to define tumor invasion boundaries. The tumor invasion boundary is the interface region between tumor tissue and nontumor tissue, which 2 board-certified pathologists determine according to the H&E images. To delineate the boundary, we devised a set of rigorous criteria for assessing the inclusion of each spot within the interface zone. We evaluated 6 adjacent spots for each spot owing to the hexagonal geometry of Visium. A spot is classified as a boundary zone if it meets the following criteria: at least 2 over 6 proximal spots annotated as “tumor” and at least 2 over 6 neighbors annotated as “nontumor” spontaneously. Spatial calibration was also considered, as the 100 μm center-to-center spot distance generates boundaries 100–200 μm wide, aligning with histopathological standards for invasive fronts.

Moreover, to better analyze the correlation between the spatial features and their relative location from the boundary, we calculated the minimum Euclidean distance to the boundary for every spot. It was further divided by the minimum distance between 2 different Visium spots to eliminate the interference of disunified scale factors. The distance to the boundary for tumor spots was artificially assigned as a negative value, while the distance for non-tumor spots was a positive value. The distance for spots within the invasion boundary was zero. As a result, we projected each spatial point onto a one-dimensional axis with an origin at the boundary.

### Neighborhood score (NS), aggregation score, and co-localization score (CS) of spatial niches

#### Neighborhood score, representing neighborhood diversity.

The neighborhood score quantifies the regional diversity of proximate niches. For each central spot, we examine its 6 nearest neighboring spots (hexagonal lattice pattern) and count the number of distinct niche categories present among these neighbors. This count ranges from 1 (all 6 neighbors belong to the same niche category, indicating homogeneous surroundings) to 6 (each neighbor represents a different niche category, indicating maximum microenvironmental diversity). The mean neighborhood score across all spots of a particular niche type reflects how diverse that niche’s typical surroundings are within the tissue architecture.

#### Aggregation score, representing self-clustering tendency.

The aggregation score (AS) measures how strongly a particular niche type tends to cluster with itself. For each spot belonging to niche X, we count how many of its 6 nearest neighbors also belong to niche X. This count ranges from 0 (completely isolated spot) to 6 (spot at the center of a perfect cluster). The aggregation score for niche X is then calculated as the average of these counts across all spots assigned to niche X. High aggregation scores indicate that the niche forms tight, self-contained clusters in the tissue, while low scores suggest more dispersed or evenly distributed patterns.

#### Co-localization score, representing interniche spatial relationships.

The colocalization score (CS) systematically evaluates spatial relationships between different niche types. To calculate niche A’s colocalization with niche B, we examined each spot belonging to niche A and counted how many of its 6 neighbors belonged to niche B. We then averaged these counts across all niche A spots. The score ranges from 0 (no spatial association) to 6 (perfect surrounding), with intermediate values indicating partial but nonrandom spatial relationships.

### Definition of single-cell gene signature scores

All single-cell gene signature scores were calculated by the R package AUCcell (version 1.16.0) (37). Cell ranking was calculated by the AUCell_buildRankings function with normalized data, and AUCcell scores were counted by the AUCell_calcAUC function with aucMaxRank equaling the row number of cell ranking * 0.1. Genesets used in T/NK cells were downloaded from the supplementary file of this study (38). Hallmark datasets were downloaded from the MsigDB database (https://www.gsea-msigdb.org/gsea/msigdb) (39).

### Cell-state spatial mapping

Cell2location was used to infer cell state abundance for each spot (40). Negative binomial regression was applied to estimate reference cell type signatures from the significant and fine-annotated single-cell datasets without downsampling. Each slide was later deconvoluted using hierarchical Bayesian models (Default) with hyperparameters’ N_cells_per_location = 10’ and ‘detection_alpha = 20.’ The models were trained using complete data (batch_size = None) with ‘max_epochs = 30000’. The 5% quantile of the posterior distribution in the Bayesian model was used to represent the value of cell abundance with high confidence.

### Transcription factor analysis

The SCENIC (37) pipeline was used to infer statistically active transcription factors (TFs) and their targets across the myeloid cells with default settings. All the myeloid cells were extracted from the scRNA-seq dataset. First, by running GENIE3 (37), SCENIC identified potential targets for each TF based on co-expression, formatting the co-expression modules. Second, it filtered indirect targets from modules using DNA motif analysis (cisTarget) (41). The remaining TF and its potential direct target were called a regulon. Finally, AUCell (37) was used to compute the activity of each regulon in each cell.

### Spatial cell-cell communication analysis

We applied the R package CellChat v2.0.0 (42) to analyze spatially proximal cell-cell communication with spatial transcriptomics data sets. For robustly computing the communication probabilities and weights among different niches, we used the computeCommuProb function with the following parameters: type = “truncatedMean”, trim = 0.1. Then, the ligand-receptor(L-R) interactions within specific niches were compared between sites using the netVisual_bubble function. To further confirm the in situ spatial interaction in the slides, we recreated the cellchat object for each slide with their tissue coordinates and scale factors JSON files. For cellchat objects with spatial info, cellular communications were recorrected with spatial distance in the computeCommunProb function with the following parameters: distance.use = TRUE, scale.distance = 1. Besides, we applied stLearn (0.4.11) (43) to compute the L-R score with spatially constrained 2-level permutation (SCTP) analysis.

### Single-cell CNV analysis

CNV was estimated by the R package inferCNV (version 1.10.1) (44). An annotation file was generated from GTF using Cell Ranger. RNA assay count matrices were used to feed inferCNV with the main cell types as annotations. Notably, all cells without epithelial cell marker gene expression were regarded as reference cells. InferCNV was run on each sample with the following parameters: cutoff=0.1, cluster_by_groups = TRUE, denoise = TRUE, and HMM = F. InferCNV outputs were scaled across genes in each cell to define the CNV scores of epithelial cells. The scaled CNV value was first subtracted from the minimum value and then divided by the range. The result was multiplied by 2 and subtracted by 1 to obtain a range of –1 to 1. Finally, the square of the above result was averaged to calculate the CNV score. The detailed calculation process was denoted as follows:
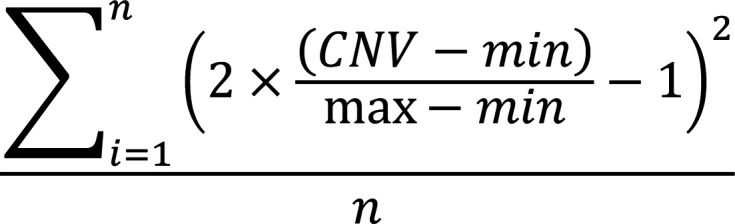


*CNV* means the scaled output of InferCNV; *min* and *max* denote the min and max values of the scaled CNV value of each cell.

### Spatial inferCNV

Since clonal evolution occurs independently within each patient’s tumor, inferCNV analysis was conducted for each patient’s data using their normal muscle tissue as a control. Tumor spots were clustered based on their CNV, with optimal classification numbers for each patient’s tumor data determined by silhouette coefficient analysis. The parameters for inferCNV and the method for calculating the CNV score were identical to scRNA-seq data, which was mentioned above.

### Gallbladder cancer cell culture

Cell lines GBC-SD, ZJU-0430, PAN02, and CT26 were derived from our laboratory preservation and cultured under standard conditions. Cells were maintained in DMEM medium (Gibco, Cat# 11965092) supplemented with 10% fetal bovine serum (FBS; Gibco, Cat# A5670701), 100 U/mL penicillin, and 100 μg/mL streptomycin (Gibco, Cat# 15140122). Cells were cultured at 37°C in a humidified atmosphere containing 5% CO_2_.

### Establishment of an orthotopic liver invasion cancer model

Male BALB/c nude mice and C57BL/6J mice were purchased from GemPharmatech Co., Ltd and housed under specific pathogen-free (SPF) conditions with a 12-hour light/dark cycle, constant temperature (22 ± 2°C), and humidity (55 ± 10%). Mice were provided with standard rodent chow and water ad libitum.

Mice were anesthetized with 2% isoflurane in oxygen and placed in a supine position. The abdominal area was disinfected with 70% ethanol, and a small incision (approximately 1 cm) below the xiphoid was made to expose the gallbladder. Using a 30-gauge needle, 20 μL of the cell suspension (1 × 10^6^ cells) was slowly injected into the gallbladder wall to establish the orthotopic tumor. Care was taken to avoid leakage of the cell suspension during injection. After implantation, the gallbladder was carefully returned to the abdominal cavity, and the incision was closed in 2 layers with 4-0 absorbable sutures.

C57BL/6J mice were used to establish an orthotopic liver tumor model with PAN02 and CT26 cell lines for the evaluation of liver invasion. To manipulate macrophage-derived CXCL9 in vivo, mice received a tail vein injection of adeno-associated virus serotype 9 (AAV9) encoding F4/80 promoter–driven constructs for either CXCL9 overexpression or siRNA-mediated CXCL9 knockdown 4 weeks before tumor implantation. Orthotopic liver tumors were then generated by implanting PAN02 or CT26 cells into the hepatic parenchyma. Two weeks after tumor implantation, mice were euthanized, and livers/tumors were collected for histological assessment of hepatic invasion. Cohorts were defined by cell line (PAN02 or CT26) and vector treatment (CXCL9 overexpression or CXCL9 knockdown).

### Flow cytometry

Primary human T cells were cocultured in vitro with gallbladder cell lines. For surface staining, cells were incubated with fluorochrome-conjugated antibodies: Pacific Blue anti-CD45, APC-Cy7 anti-CD3, AF700 anti-GZMB, BV510 anti-CD8, PE-Cy7 anti–PD-1, PE anti-CTLA-4, BV605 anti-TIGIT, and APC anti-CXCL13 for 30 minutes at 4°C. Following staining, cells were washed with PBS and fixed with 1% paraformaldehyde. Flow cytometric analysis was performed using a BD LSRFortessa X-20, and data were analyzed with FlowJo software. Appropriate compensation controls were included to ensure accurate measurement of fluorescence.

### Immunofluorescence staining of tissue

Formalin-fixed, paraffin-embedded (FFPE) tissue sections (4 μm thick) were deparaffinized in xylene and rehydrated through a graded series of ethanol to water. Each primary antibody was diluted in the Antibody Diluent/Block and applied to the sections. Incubation with each primary antibody was performed overnight at 4°C. Multiplex immunofluorescence staining was performed using the Opal 7-Color Manual IHC Kit (Akoya Biosciences, Cat# NEL811001KT) according to the manufacturer’s instructions. All the detailed information of primary antibodies is listed in Supplemental Table 10. 

### Abbreviations

For a list of abbreviations, see Supplemental Table 11.

### Statistics

For comparisons between two groups, 2-tailed Student’s t-test or Wilcoxon rank-sum test was used. Specifically, for cell-state spatial enrichment analysis, the differences of scaled median cell type composition among groups on the slide basis were examined by the 1-sided Wilcoxon rank-sum test. For differentially expressed genes analysis, a Wilcoxon rank-sum test with Bonferroni-adjusted *P*-values was obtained for each gene. For multiple group comparisons, 1-way ANOVA (parametric) or Kruskal-Wallis (nonparametric) tests were applied. Categorical variables were analyzed using the chi-square test or Fisher’s exact test. Survival analysis was performed using the Kaplan–Meier method, and differences between groups were assessed by the log-rank test. Multivariable Cox proportional hazards regression models were used to adjust for potential confounding factors. Correlation analysis was performed using Pearson or Spearman methods, depending on data distribution.

### Study approval

This study was approved by the Ethics Committee of Renji Hospital Affiliated to Shanghai Jiao Tong University School of Medicine, with approval No. KY2024-003-C. All patients provided informed consent, and the study was performed following the Declaration of Helsinki. All animal experiments were conducted according to institutional guidelines and approved by the Institutional Animal Care and Use Committee at Xinhua Hospital Affiliated with Shanghai Jiao Tong University School of Medicine (XHEC-QT-2023-016).

### Data availability

The raw sequence data reported in this paper have been deposited in the Genome Sequence Archive in National Genomics Data Center (HRA008543) and OMIX (OMIX007397), China National Center for Bioinformation / Beijing Institute of Genomics, Chinese Academy of Sciences are accessible at https://ngdc.cncb.ac.cn.The pipeline POSST is available at https://github.com/zaozaozaonan/POSST; commit ID 753373d6f1bfcd19462c889ec1f42cdae1d8209e.

## Author contributions

The order of the cofirst authors was determined based on their overall contribution to the project leadership and manuscript development. Cofirst authors: ML: cosupervision; Zhaonan Liu: formal analysis, data curation, writing (original draft), revision, sample collection, validation (patient cohorts); SS: formal analysis, writing (original draft), conceptualization, revision; ZJ: validation (animal models, Western blot, mIHC staining, flow cytometry), writing (original draft), revision; Yongsheng Li: investigation, sample collection. Corresponding authors: Yingbin Liu: conceptualization, resources, supervision; W Wei: resources, supervision, writing (review & editing); RS and KM: writing (review & editing). Other authors: LL, C Li, and YH: methodology; XX: project administration; Yun Liu: visualization; SQ, LG, Yuanding Liu, XL, ZW, SY, JM, C Liu, and Zebing Liu: Resources; ZY, JF, and PG: data curation; FL, YZ, W Wu, XW, WG, Yanjing Li, DX, Yiming Li: investigation.

## Funding suppport

- National Natural Science Foundation of China (32130036, 81870187, 81911530167, 82073206).
- Shanghai Shenkang Clinical Technology Innovation Project (SHDC12021101).
- Basic Research Project of Science and Technology Commission of Shanghai Municipality (20JC1419100).
- Shanghai Rising-Star Program (23QA1408500).
- Young Talents Project of Shanghai Municipal Health Commission (2022YQ061).
- Science and Technology Innovation Action Plan Technical Standards Project of Science and Technology Commission of Shanghai Municipality (23DZ2202800).
- Cooperative Research Projects of Shanghai Jiao Tong University (2022LHA13).
- Horizontal project of Renji Hospital (RJKY-23-002, RJKY23-001, RJKY24-002).
- Program of Shanghai Outstanding Academic Leader [23XD1450700].
- Major Science and Technology R&D Project of the Science and Technology Department of Jiangxi Province (20213AAG01013).
- National Key R&D Program of China (2021YFF0703802).
- The National Key Research and Development Program of China (2021YFE0203300).
- The Shuguang Program of Shanghai Education Development Foundation and Shanghai Municipal Education Commission (20SG14).
- Shanghai Municipal Health Commission Grants 202140050.
- Shanghai Jiao Tong University Medical Engineering Cross Fund (YG2022ZD006).
- Shanghai Municipal Health Commission Health Industry clinical research special project (20224Z0014).
- National Clinical Key Specialty Construction Project (10000015Z155080000004).
- High-level Talent Cultivation Program (Category A) of Renji Hospital Shanghai Jiaotong University School of Medicine.
- Shanghai Leading Talent Program of Eastern Talent Plan (LJ2025102).
- Guizhou Provincial Major Scientific and Technological Program.
- Peak Plateau Discipline Construction Project of Shanghai Jiao Tong University School of Medicine (20181808).
- Lingang Laboratory (LGL-8888).
- National Science and Technology Major Project Funding of China (2025ZD0552412).
- Basic Research Project of Shanghai Cancer Institute and Shanghai Runda Rongjia Biotechnology Co., Ltd (SYXF0120022383).

## Supplementary Material

Supplemental data

Unedited blot and gel images

Supplemental table 1

Supplemental table 2

Supplemental table 3

Supplemental table 4

Supplemental table 5

Supplemental table 6

Supplemental table 7

Supplemental table 8

Supplemental table 9

Supplemental table 10

Supplemental table 11

## Version 1 03/02/2026

Electronic publication
