# Supplementary material for: Macrophage-rich niches regulate T cell dynamics at the liver invasive margin during gallbladder cancer progression
Source: J Clin Invest. 2026 Mar 2;136(5):e193672. doi: 10.1172/JCI193672 (PMC12948426; doi:10.1172/JCI193672)
Supplement: Supplemental data [file jci-136-193672-s034.pdf]

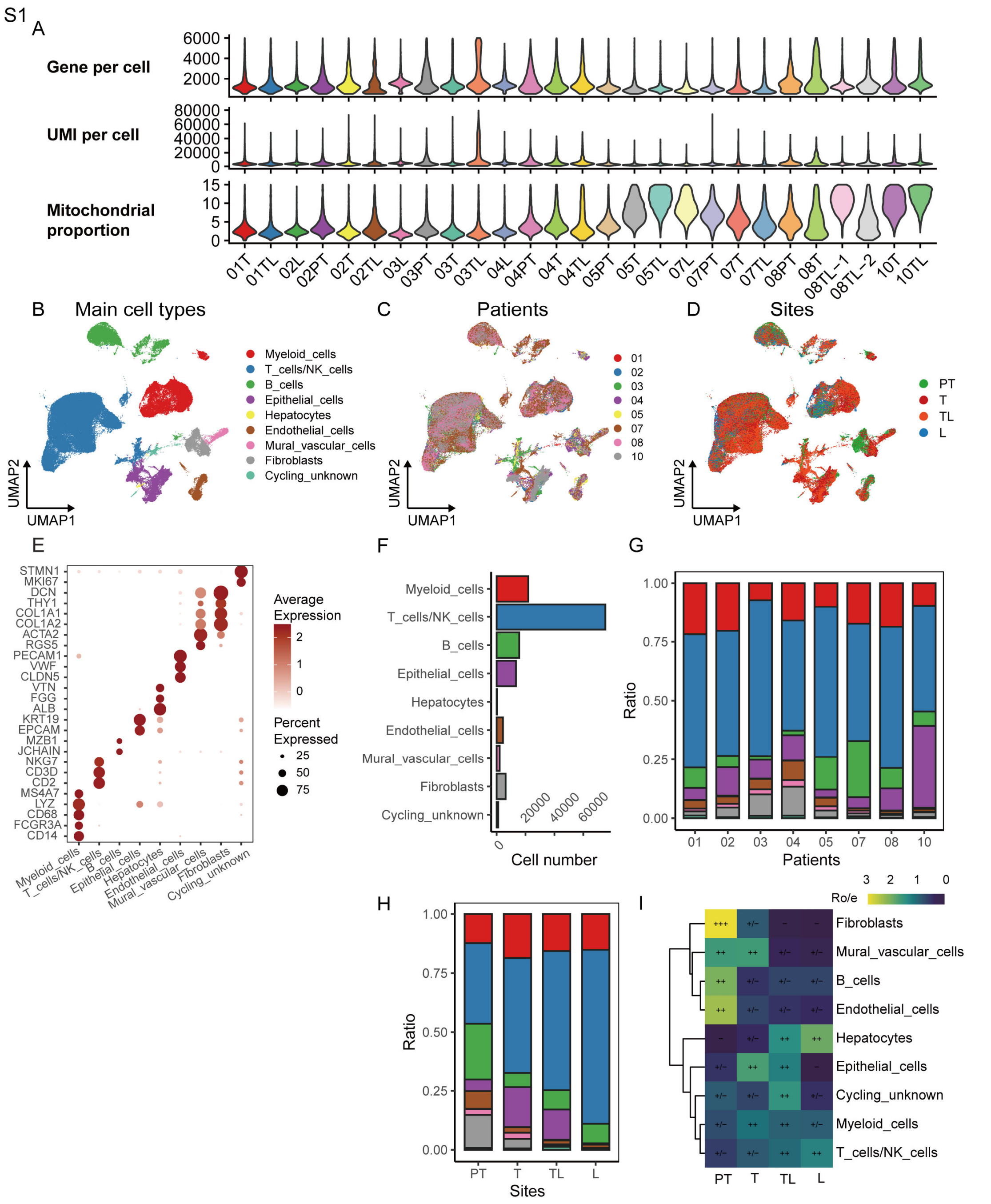

Figure S1. Single-cell landscape of gallbladder cancer with direct liver invasion, related to Figure 1

(A) Quality control of the single-cell data sets. 27 samples from 8 gallbladder cancer (GBC) patients were subjected to profiling using single-cell transcriptomics. Genes & unique molecular identifiers (UMIs) detected per cell, and mitochondrial proportions were measured.

(B) Uniform manifold approximation and projection (UMAP) representation of all single-cell transcriptomes for 9 main cell types.

(C) UMAP representation of all single-cell transcriptomes for 8 patients.

(D) UMAP representation of all single-cell transcriptomes for 4 different sites. PT, para-tumor; T, tumor; TL, liver invasion boundary; L, liver.

(E) Bubble plot showing marker gene expression of cell types.

(F) Bar plots showing cell numbers of 9 different cell types.

(G) The distribution of cell types in each patient. The colors were the same as those shown in (F).

(H) Distribution of cell types in 4 sampling sites. The colors were the same as those shown in (F).

(I) Ro/e heatmap showing the relative abundance of main cell types across 4 sampling sites.

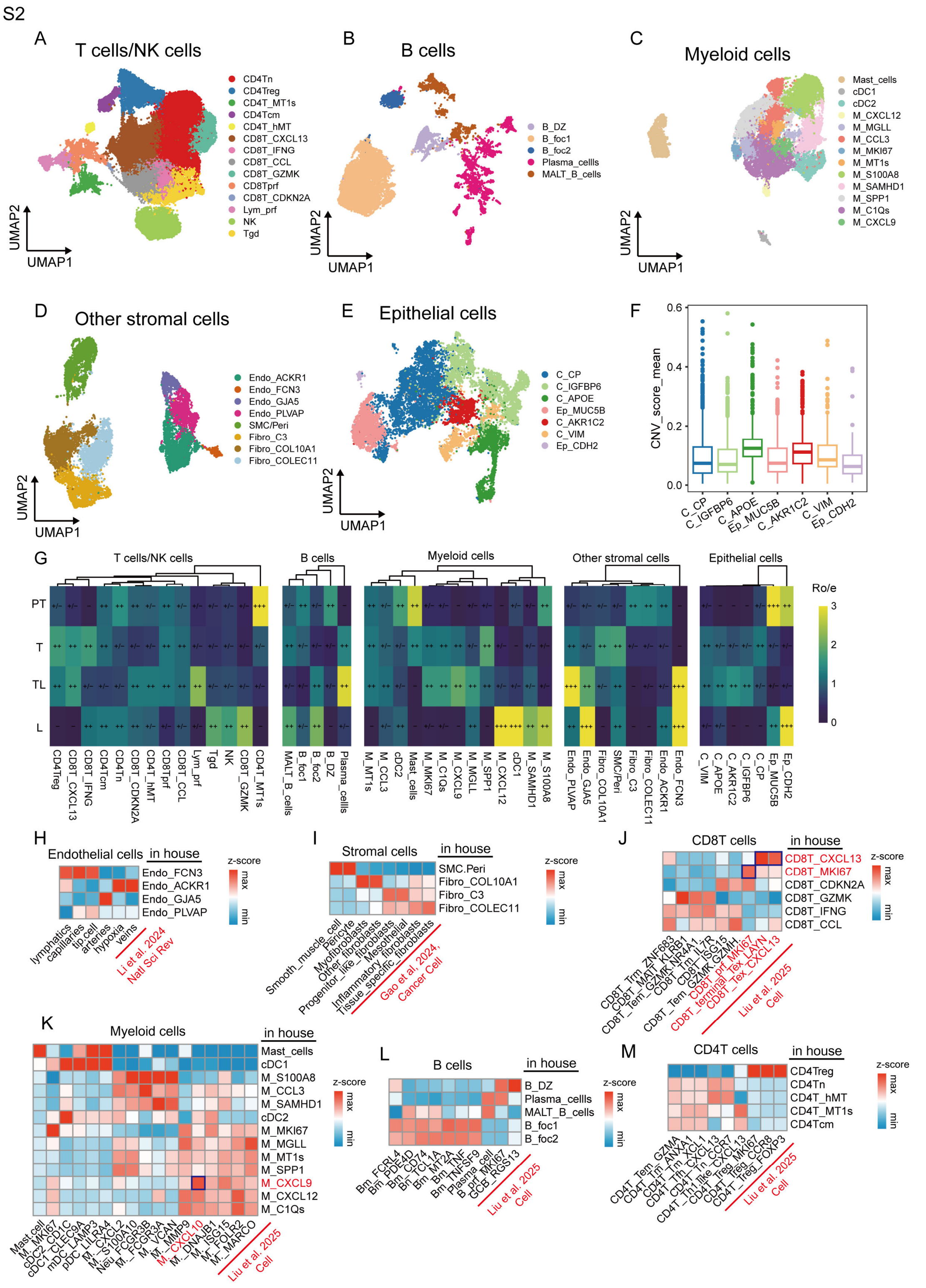

Figure S2. The single-cell landscape of cellular subtypes, related to Figure 1

(A-E) UMAP representation of all subtypes of T/NK cells (A), B cells (B), myeloid cells (C), stromal cells (D), and epithelial cells (E) from the above samples in Figure S1.

(F) CNV score of epithelial cell subsets.

(G) Ro/e heatmap showing the relative abundance of cell subsets across different sampling sites.

(H-M) Heatmap of scaled correlation among in-house cell substates (rows) and cell types in published datasets (columns).

S3

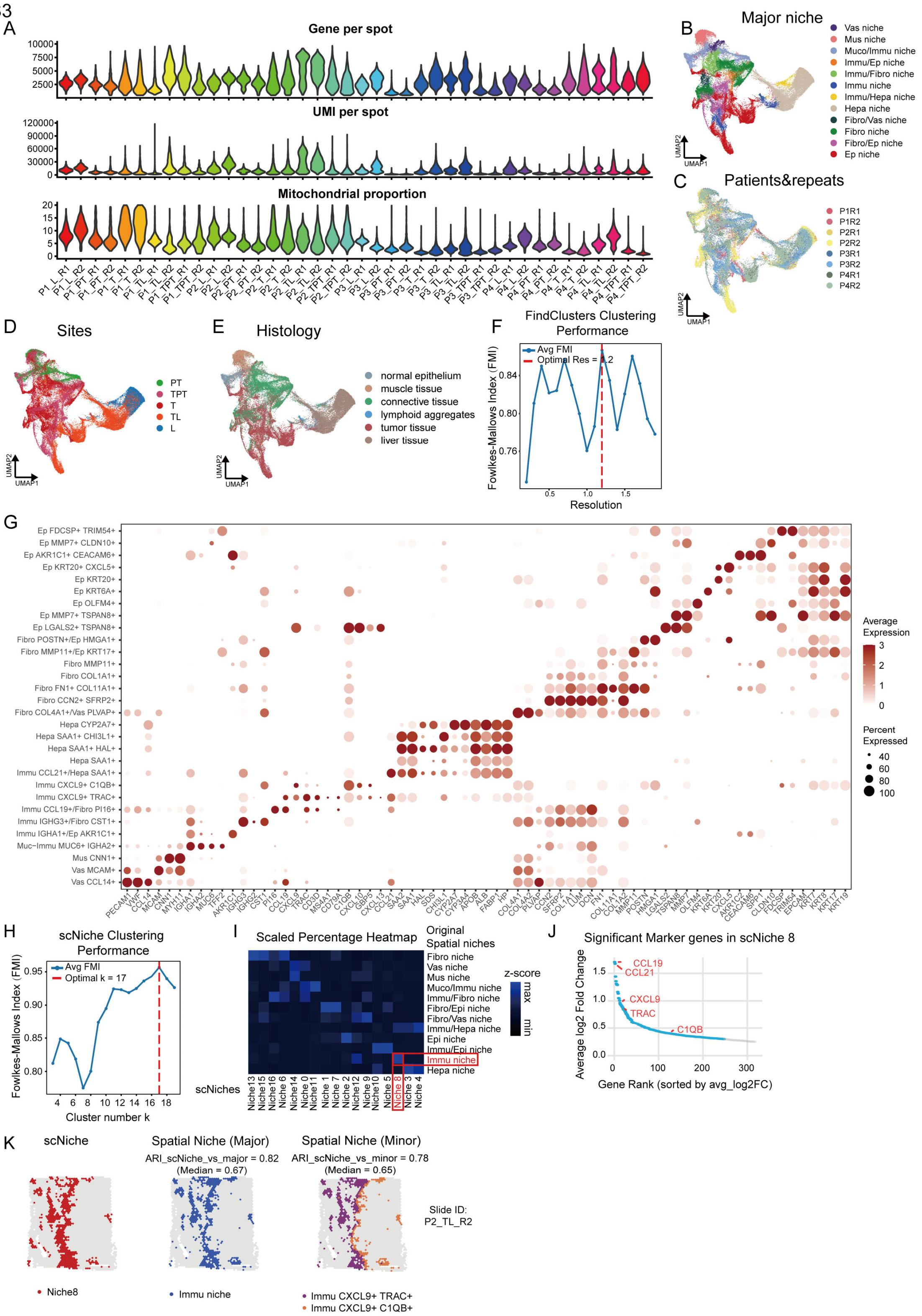

Figure S3. Spatial niches identification, related to Figure 1

(A) Quality control of the spatial transcriptomics data sets from 40 samples of 4 GBC patients revealed the number of genes and unique molecular identifiers (UMIs) detected per spot, as well as the mitochondrial proportion.

(B-E) UMAP plot of 12 spatial niches (B), 8 patients and repeats (C), 4 sampling sites (D), and 6 histological regions (E) with integrated spatial transcriptome.

(F) Clustering performance quantified with Fowlkes-Mallows Index (FMI) among different resolutions with FindClusters. The optimal resolution was labeled when the FMIs reached peak.

(G) Bubble plot showing marker genes of spatial sub-niches.

(H) Clustering performance quantified with Fowlkes-Mallows Index (FMI) among different cluster numbers with scNiche. The optimal k value was identified when the FMI reached its peak.

(I) Heatmap of the scaled percentage of scNiche-derived niches (columns) among original spatial niches (rows).

(J) Dot plot showing the top significant marker genes of niche 8 from scNiche. Genes were sorted according to their average log2FC.

(K) Representative spatial plot for scNiche 8 (left), Immu niche (middle), and Immu CXCL9+ TRAC+/ Immu CXCL9+ C1QB+ niches (right) of liver invasion (TL) slides in Patient 2. Adjusted Rand Indexes (ARI) between niches identified with scNiche and the original major/minor spatial niches in the corresponding slides are labeled.

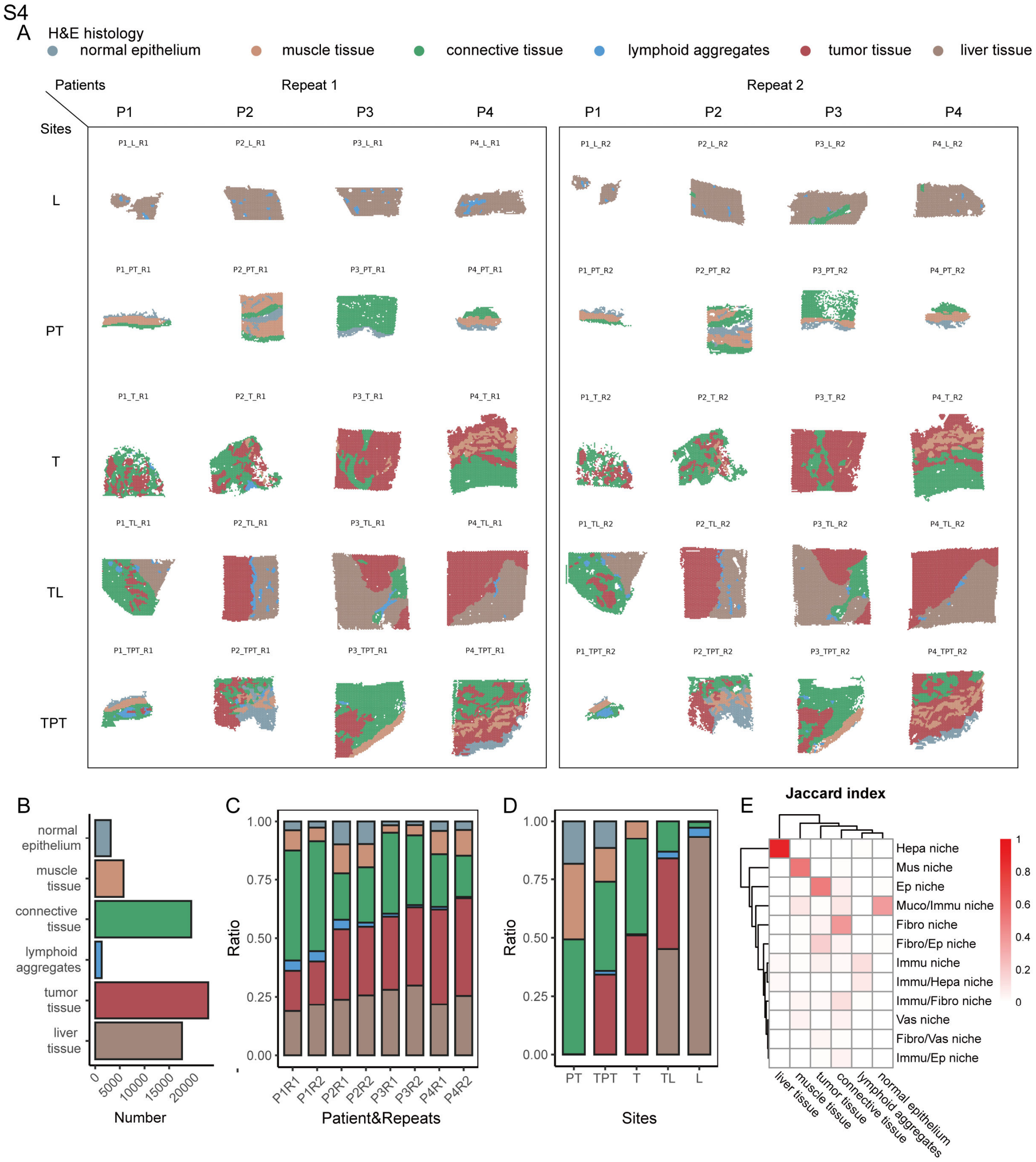

Figure S4. Histology of the spatial transcriptomics, related to Figure 1

(A) Histology annotation of all the spatial transcriptomics slides.

(B) Bar plot showing 6 histological regions.

(C) Relative distributions of 6 histological regions in each cancer sample. The colors were the same as those shown in (A).

(D) Relative distributions of 6 histological regions in each sampling site. The colors were the same as those shown in (A).

(E) Jaccard index between spatial niches and histological regions.

S5

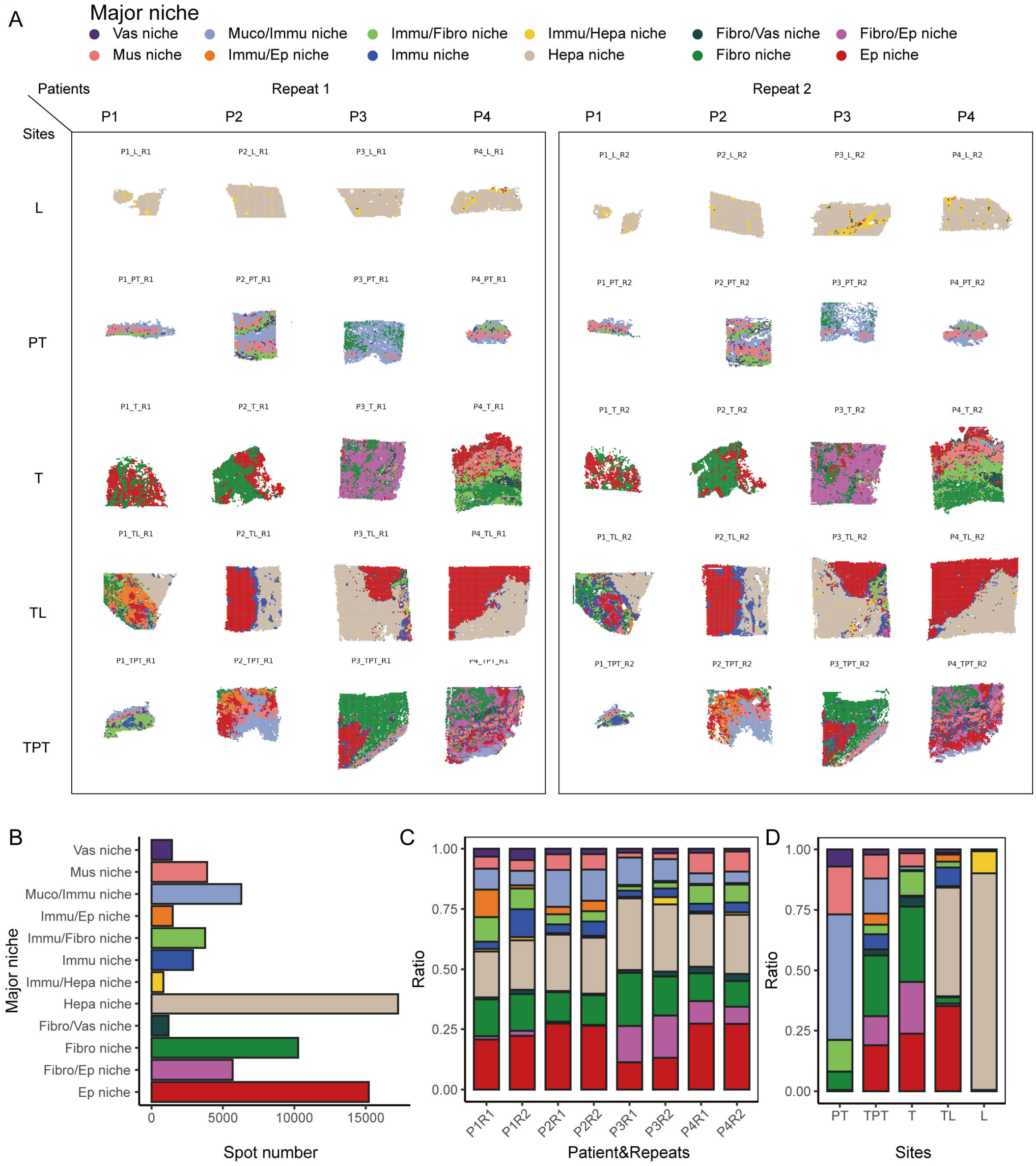

Figure S5. Niche histology of Spatial transcriptomics, related to Figure 1

(A) Spatial mapping of niches in all the slides.

(B) Bar plot showing spot number of 12 spatial niches.

(C) Relative distributions of 12 spatial niches in individual patients and repeats. The colors were the same as those shown in (A).

(D) Relative distributions of 12 spatial niches in 5 different sampling sites. The colors were the same as those shown in (A).

S6A

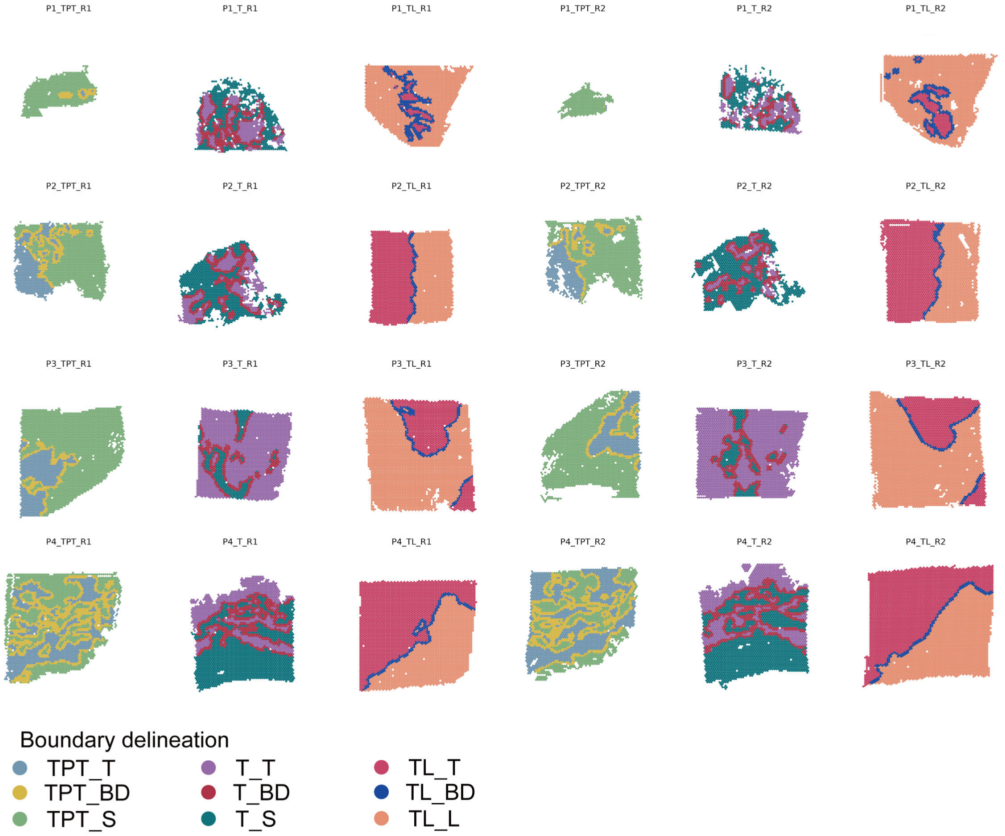

Figure S6. Boundary delineation of tumor samples, related to Figure 2

(A) Boundary delineation in tumor slides. TPT_T, tumor subregion of tumor-para-tumor invasion (TPT); TPT_BD, invasion boundary of TPT; TPT_S, stroma subregion of TPT; T_T, tumor subregion of tumor core (T); T_BD, invasion boundary of T; T_S, stroma subregion of T; TL_T, tumor subregion of tumor-liver invasion (TL); TL_BD, invasion boundary of TL; TL_L, liver subregion of TL.

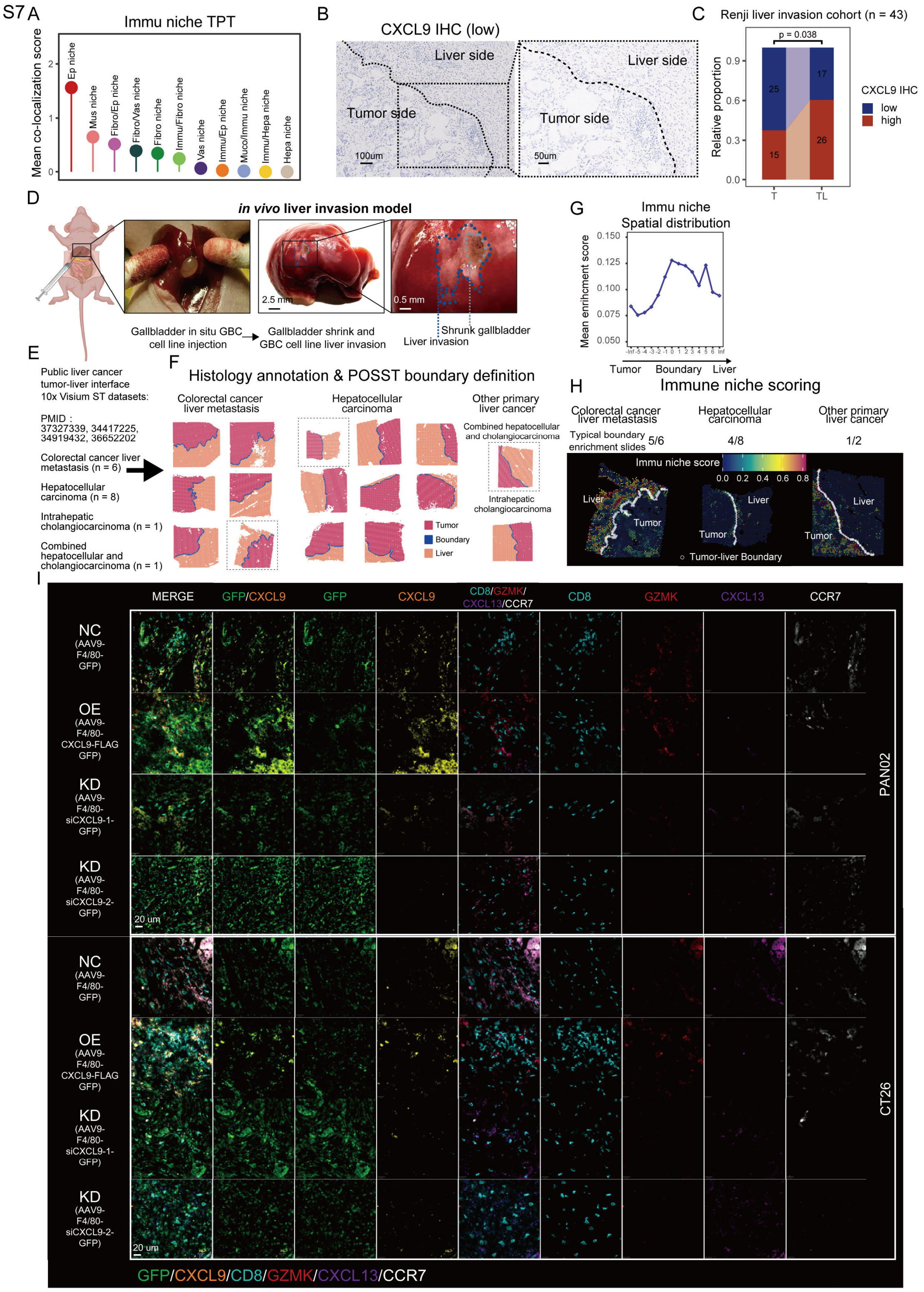

Figure S7. Spatial distribution of CXCL9+ Immune niches *in vivo* and among liver cancer tissues, related to Figure 2

(A) Co-localization score between the Immu niches and their neighboring niches in the TPT region of GBC.

(B) IHC score for CXCL9 low levels in gallbladder cancer patients with liver invasion.

(C) Column plot showing the relative proportion of CXCL9 IHC score in the tumor core (T)

region and liver invasion boundary in 43 GBC patients.

(D) The schematic diagram shows an animal model for GBC liver invasion in mice.

(E) & (F) Public liver cancer 10x Visium datasets (E) filtered for histologic annotation and boundary definition with POSST analysis (F).

(G) Line graphs showing the average enrichment score of the immune cell niche (Immu niche) in all 16 boundary slides. Each layer was about 100 μm in width.

(H) Spatial distribution of the Immu niche in representative boundary slides of colorectal cancer liver metastasis (left), hepatocellular carcinoma (middle), and combined hepatocellular & cholangiocarcinoma (right).

(I) The mIHC staining of liver invasion region in immunocompetent C57BL/6J mice with PAN02 and CT26 cell lines invading the liver. CXCL9 was either knocked out or overexpressed in macrophages using an adeno-associated virus 9 vector (AAV9).

S8

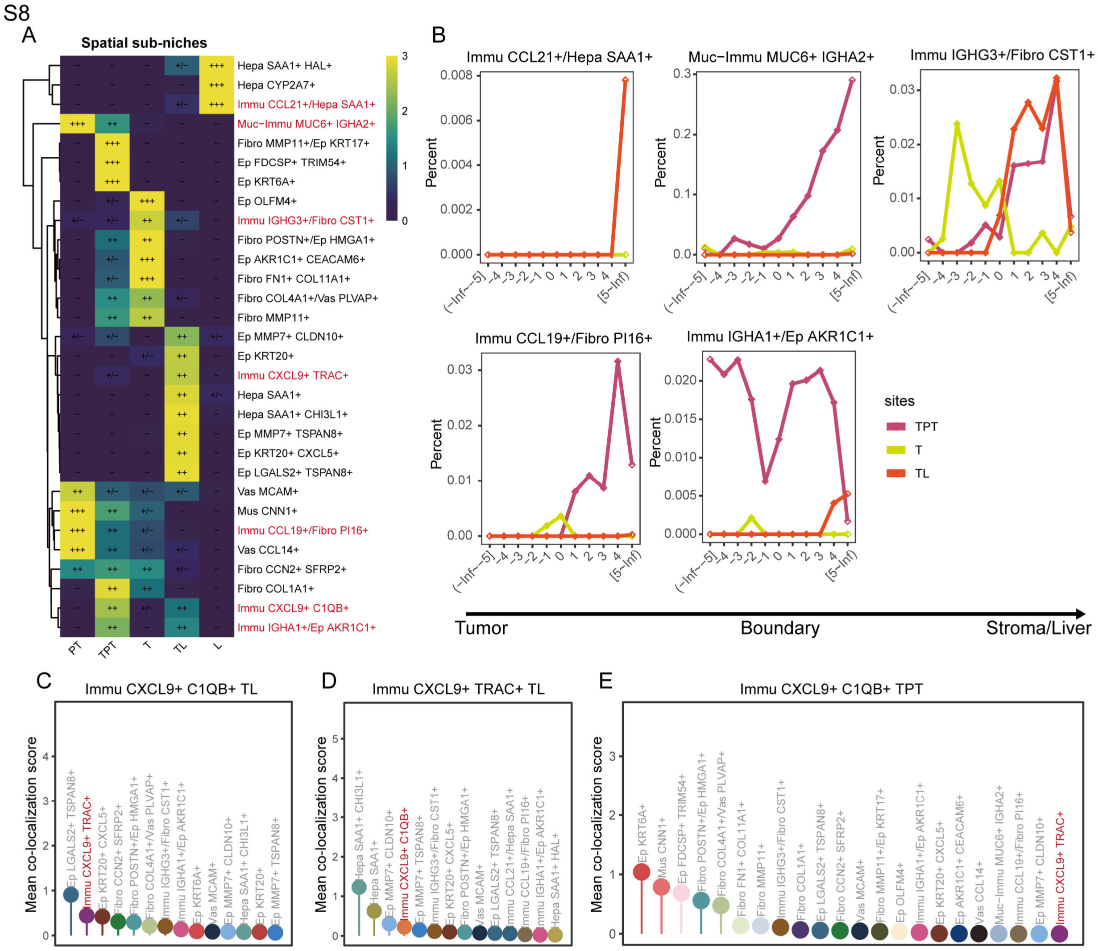

Figure S8. Co-localization of CC and CT niches in TL sites, related to Figure 3

(A) Ro/e heatmap showing the relative abundance of spatial sub-niches across different sampling sites. Immune-cell-associated sub-niches were labeled with red words.

(B) Line graphs showing the average fraction of Immune-cell-associated sub-niches labeled with red (above Figure S8A) in different spatial sites based on spatial data. Each layer was about 100 μm in width.

(C-E) Lollipop graphs show the rank of mean co-localization scores of each sub-niche surrounding CXCL9+C1QB+ (CC) niche in TL (C), CXCL9+TRAC+ niche in TL (D), and CC niche in TPT (E).

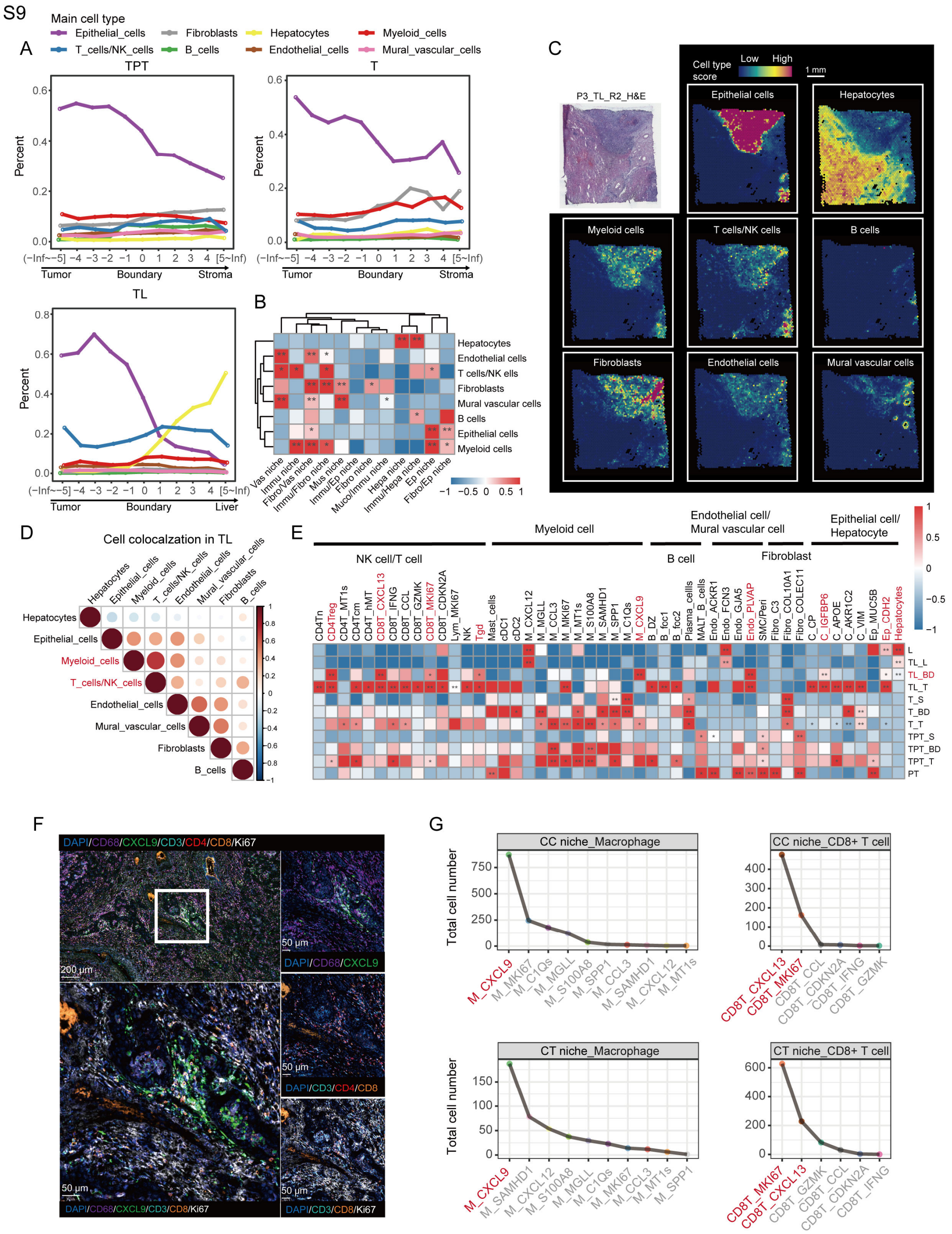

Figure S9. Spatial distribution and co-localization of cell states, related to Figure 4

(A) Line graph showing the average percentage of cell type distribution via cell2location in different spatial layers. Each layer was about 100 μm in width.

(B) Heatmap showing the relative enrichment of cell types in spatial niches. Asterisks indicate the increased cell types in a niche compared with other niches. Blank, not significant; *, $p < 0.05$ **; $p < 0.01$.

(C) H&E stain and corresponding spatial mapping of cell types in a representative TL region.

(D) Correlation heatmap illustrating spatial co-localization of cell types at the TL site. Dot size and color represented the value of the correlation coefficient.

(E) Heatmap showing the relative enrichment of cell subtypes in different subregions using cell2location. Asterisks indicate the increased cell types in subregions compared with the other subregions. Words marked with red indicate significant enrichment in the tumor-liver invasion boundary (TL_BD). Blank, not significant; *, $p < 0.05$ **; $p < 0.01$.

(F) Representative mIHC staining of CXCL9+ macrophages and proliferative CD8+ T cells in TL tissues.

(G) Subcellular distribution of macrophages and CD8+ T cells in Immu CXCL9+C1QB+ and CXCL9+TRAC+ niches.

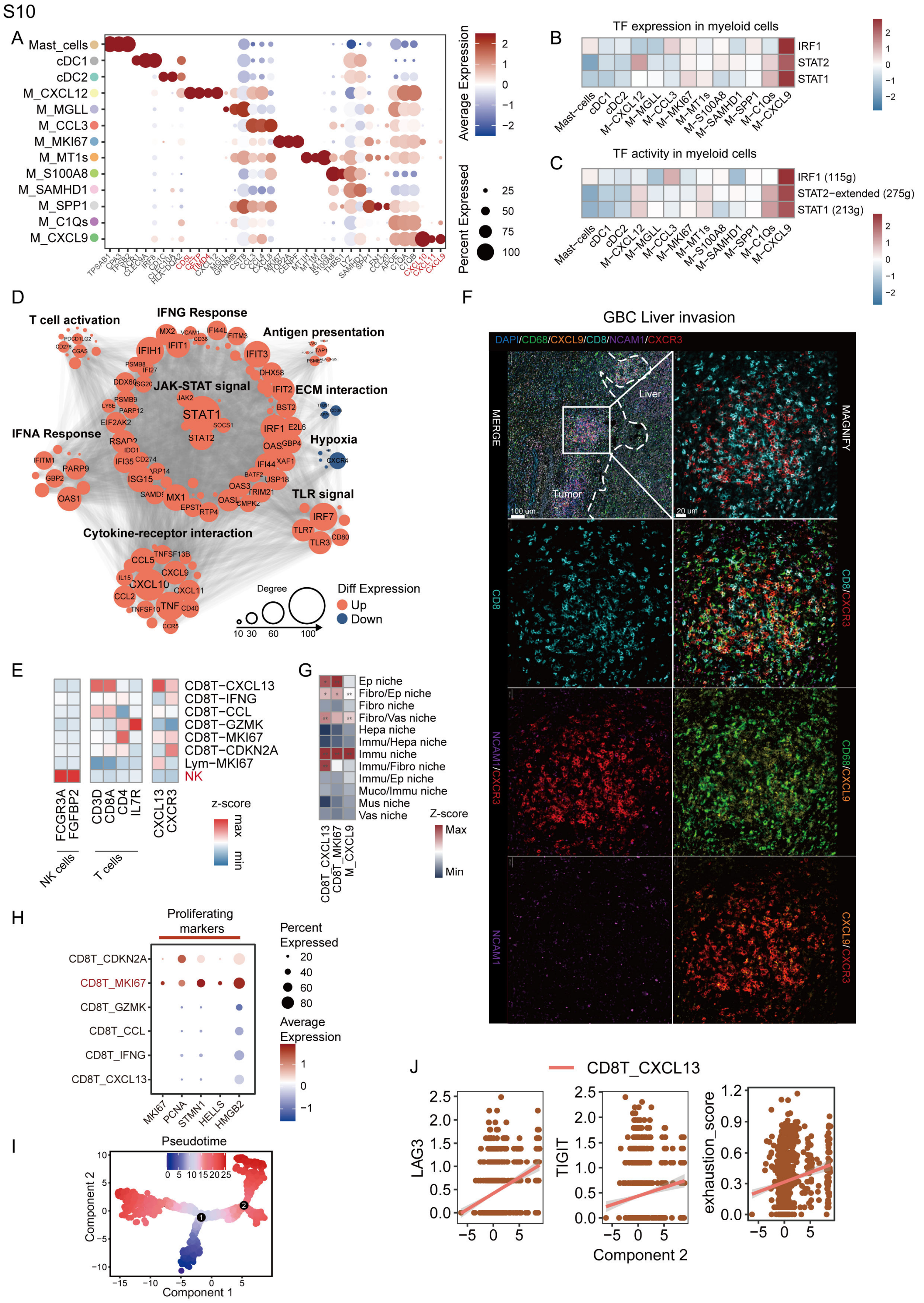

Figure S10. Single-cell characteristics of CXCL9+ macrophages and CD8+ T cells, related to Figure 5

(A) Bubble plot showing marker gene expression of myeloid cells.

(B) Transcription factors (TFs) expression in myeloid cells.

(C) TF transcriptional activity in myeloid cells.

(D) Network diagram showing the connection of JAK-STAT signal genes of CXCL9+

macrophages (M_CXCL9) with other genes or cellular responses. Genes were clustered based on their associated pathways. Each circle represented one gene. The size of the circle indicates the number of genes connecting with other genes. The color of the circle indicated whether the gene was up (orange) or down (blue) in M_CXCL9.

(E) Scaled heatmap of marker gene expression among NK cells and CD8+ T cells.

(F) GBC liver invasion tissue with mIHC staining.

(G) Relative abundance of CXCL13+ CD8+ T cells (CD8T_CXCL13), proliferative MKI67+ CD8+ T cells (CD8T_MKI67), and M_CXCL9 in spatial niches.

(H) Dot plot showing proliferating marker expression in CD8+ T cells.

(I) Pseudotime trajectory of CD8+ T cells originating from Lym_MKI67 and CD8T_MKI67.

(J) Changes of exhaustion-associated gene expression and levels of CD8T_CXCL13 in the exhaustion trajectory.

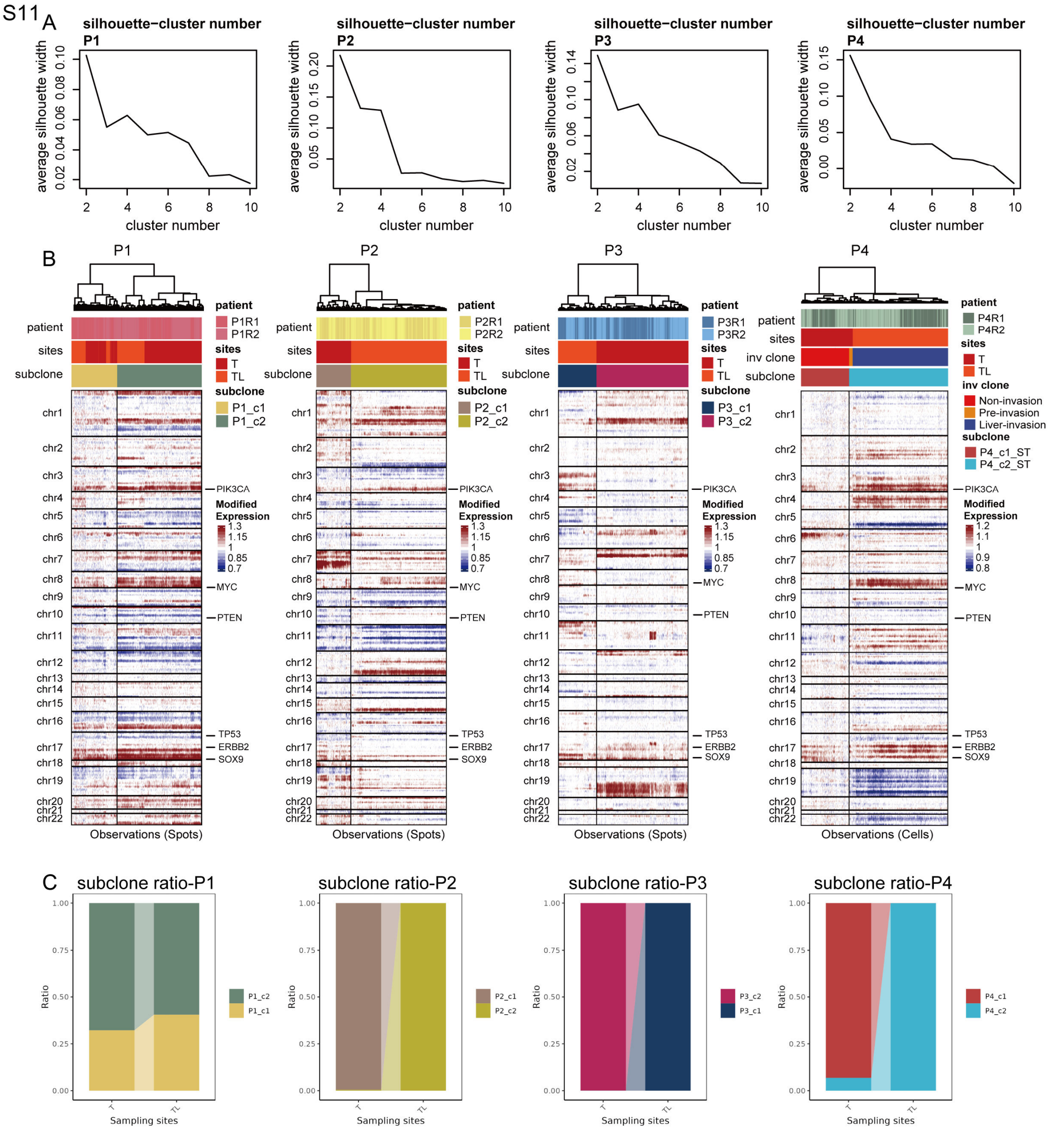

Figure S11. CNV-based tumor subclone identification, related to Figure 6

(A) Line graph showing the average silhouette width across different numbers of spatial CNV tumor subclones for hierarchical clustering in 4 patients.

(B) CNV heatmap of tumor spots for 4 patients using spatial transcriptomics.

(C) Subclone ratios of C1 and C2 in T and TL sampling sites for 4 patients.

S12

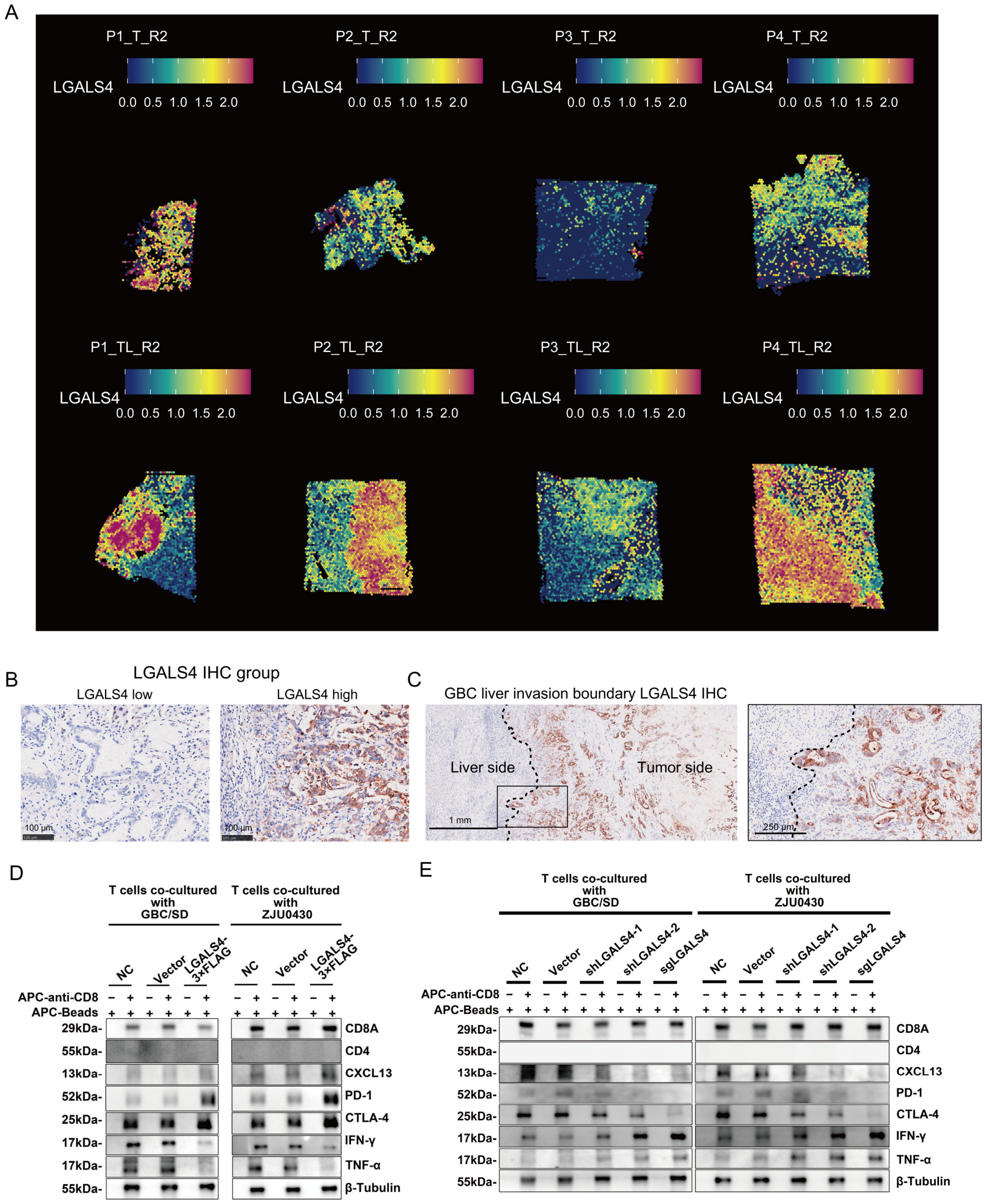

Figure S12. LGALS4 expression in TL tissues, related to Figure 6

(A) Spatial mapping of *LGALS4* expression in T and TL sites.

(B) Representative IHC staining images of LGALS4 high and low tissues.

(C) Representative images of LGALS4 IHC staining with high expression in tumor cells of gallbladder cancer (GBC) liver invasion boundary.

(D) & (E) Exhaustion- and cytotoxic-associated gene expression was detected using western blot in CD8+ T cells co-cultured with GBC cell lines. LGALS4 was overexpressed (D) and knockdown or knockout (E) in GBC cells.

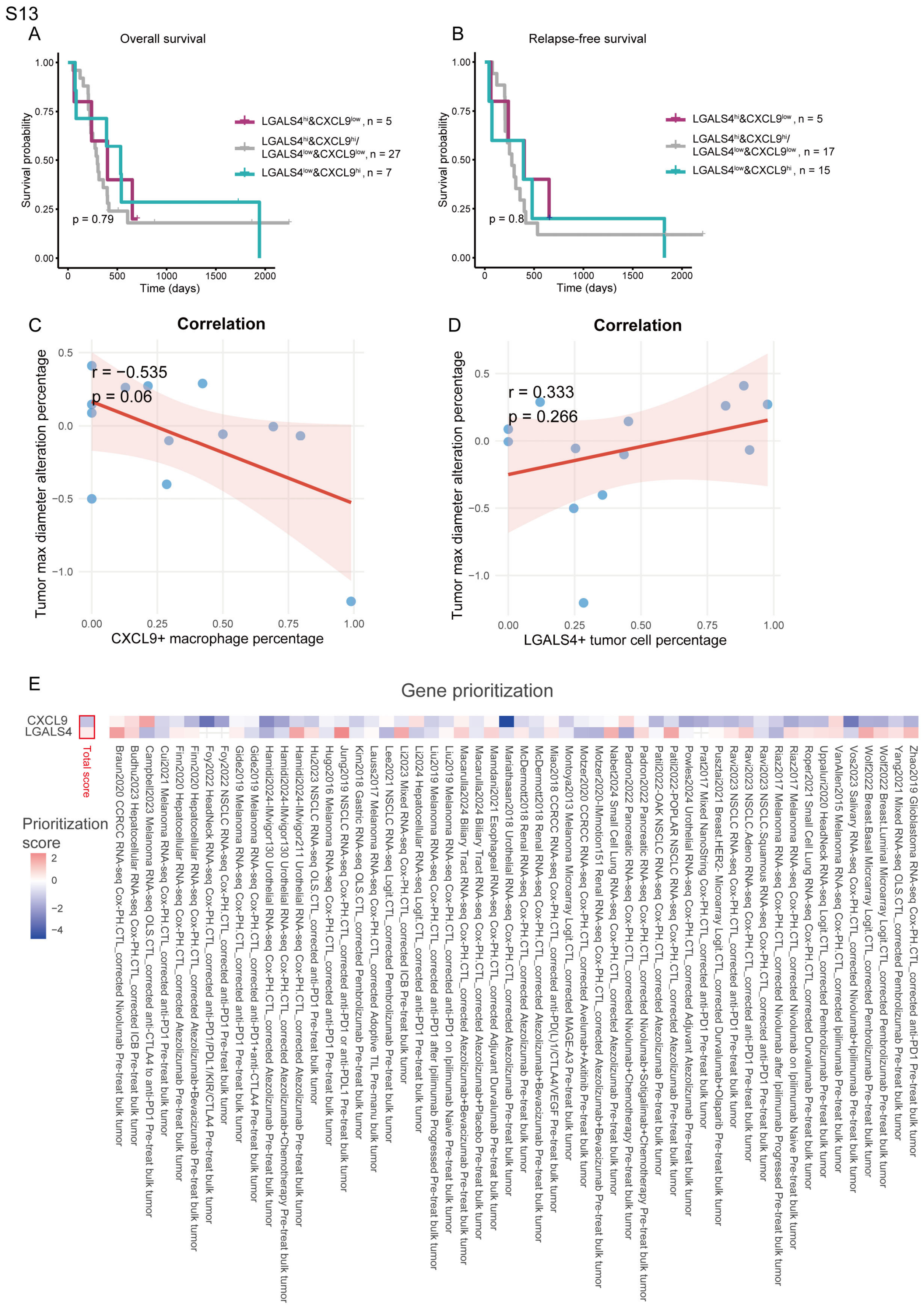

Figure S13. The combination of CXCL9 and LGALS4 in liver invasion tissue predicted prognosis in GBC patients, related to Figure 7

(A) & (B) The Kaplan–Meier overall survival (A) and relapse-free survival (B) curves of GBC liver invasion patients stratified by CXCL9&LGALS4 IHC scores in tumor core tissues.

(C) & (D) Correlation of tumor diameter change (post-treatment minus baseline) with percentages CXCL9+ macrophage (C) or LGALS4+ tumor cell (D). Pearson correlation r

and *p* values are shown.

(E) Gene prioritization score of CXCL9 and LGALS4 in Cancer Immunology Data Engine (CIDE) database.
