# Supplementary material for: Macrophage-rich niches regulate T cell dynamics at the liver invasive margin during gallbladder cancer progression
Source: J Clin Invest. 2026 Mar 2;136(5):e193672. doi: 10.1172/JCI193672 (PMC12948426; doi:10.1172/JCI193672)

Figure 6G GBC-SD

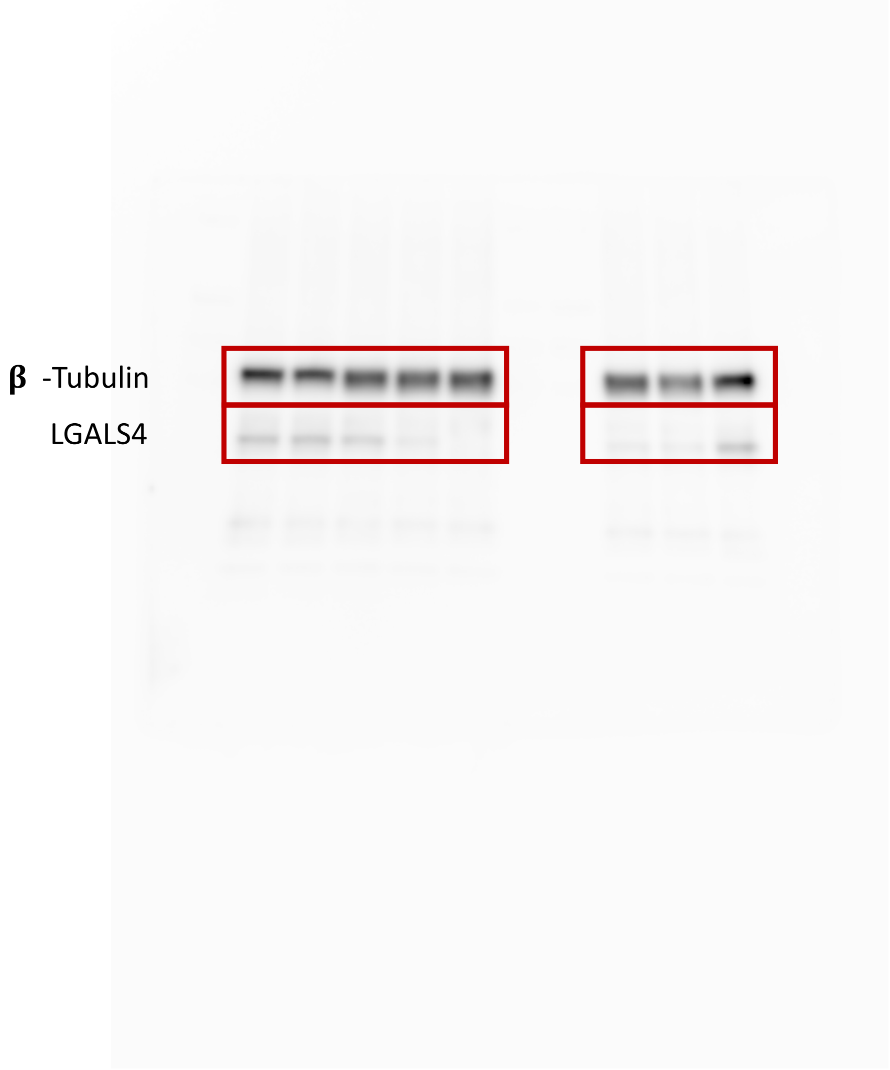

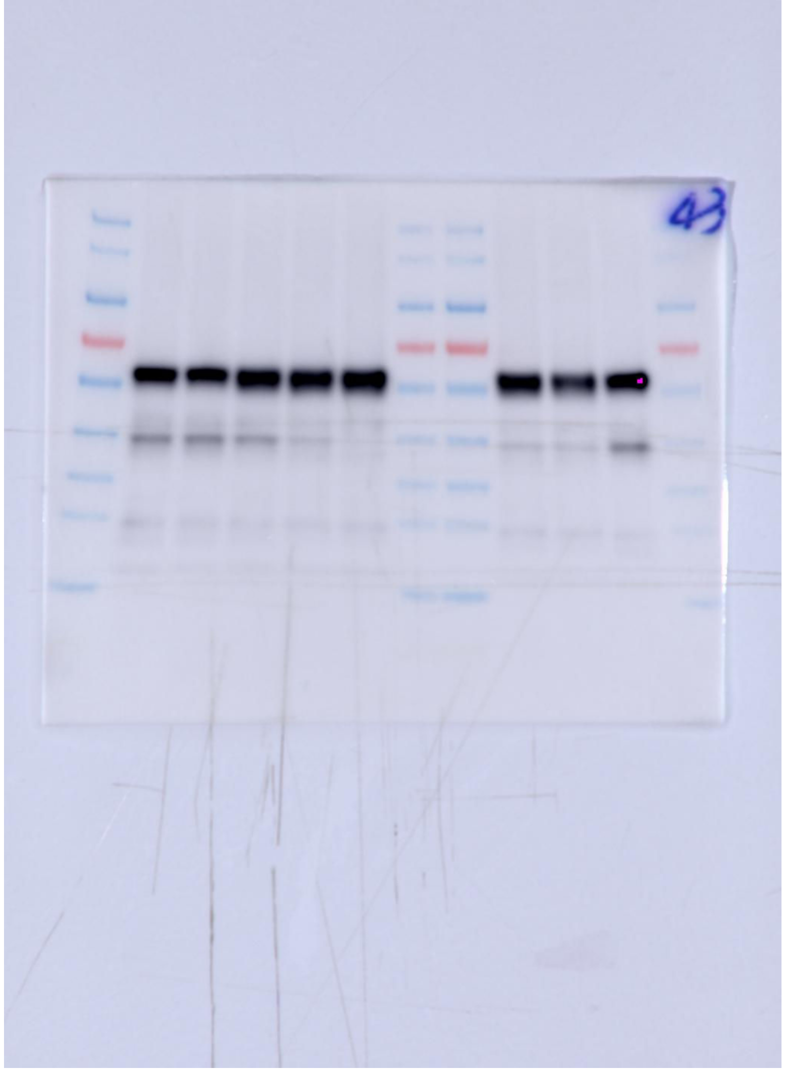

Figure 6G ZJU-0430

β -Tubulin

LGALS4

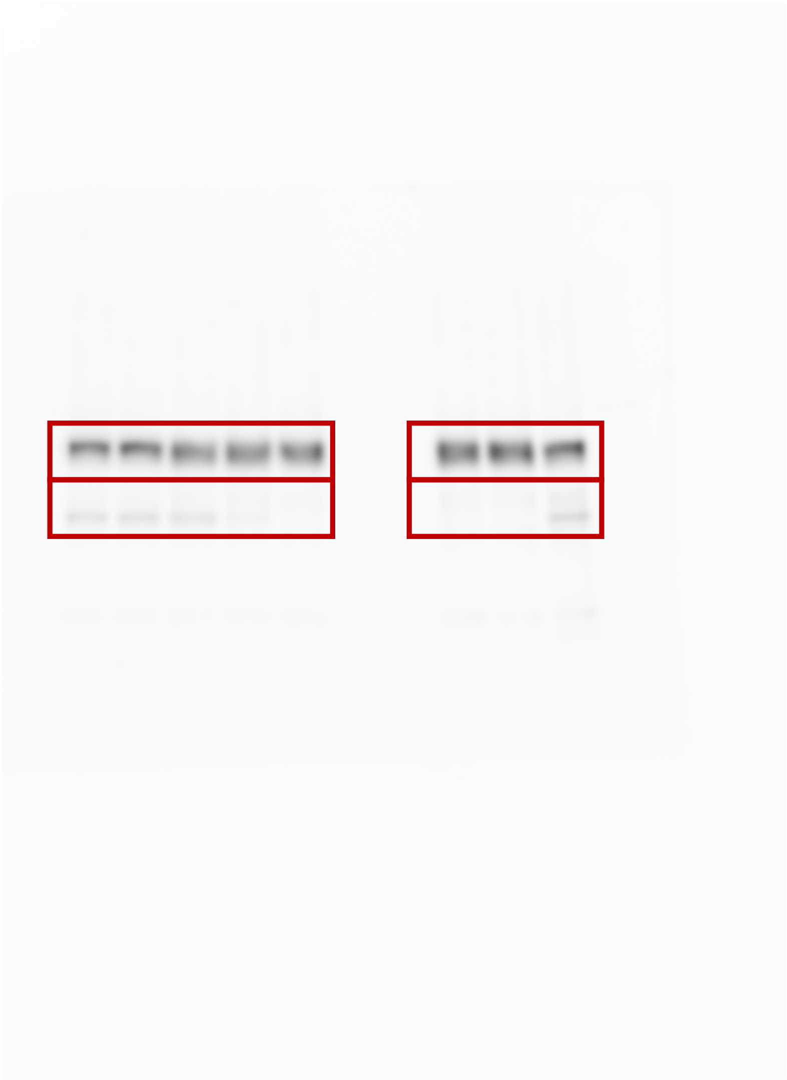

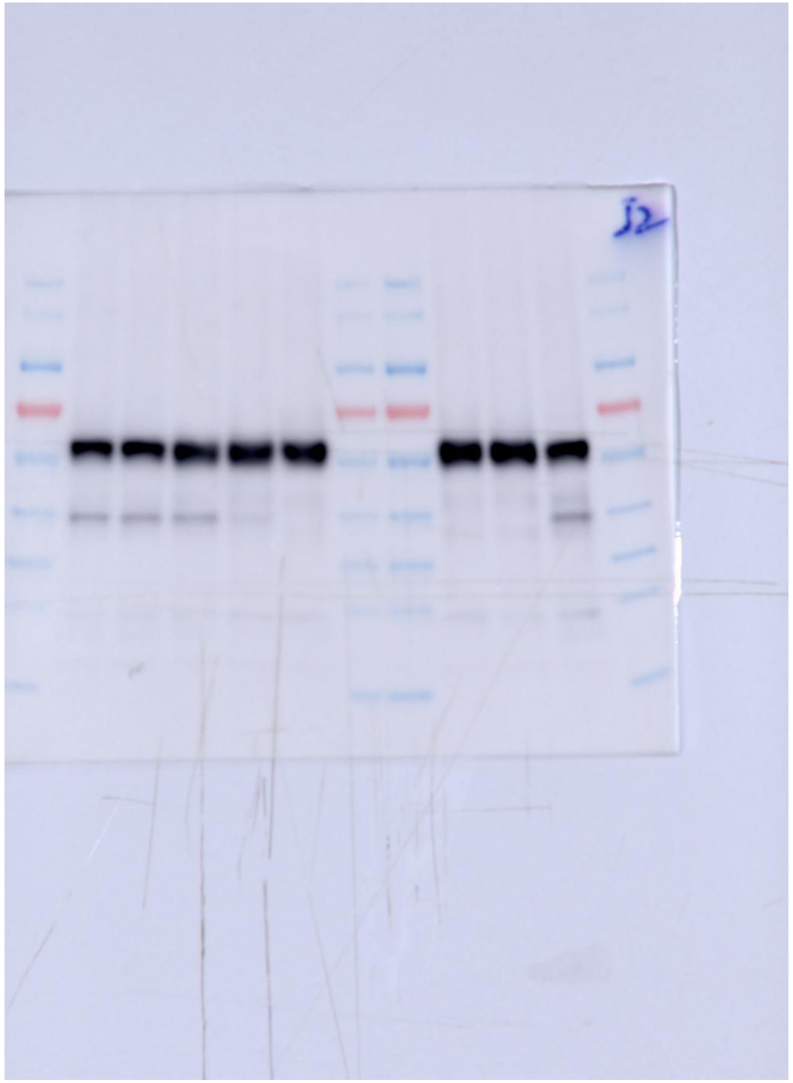

Figure S12D T cells co-cultured with GBC/SD

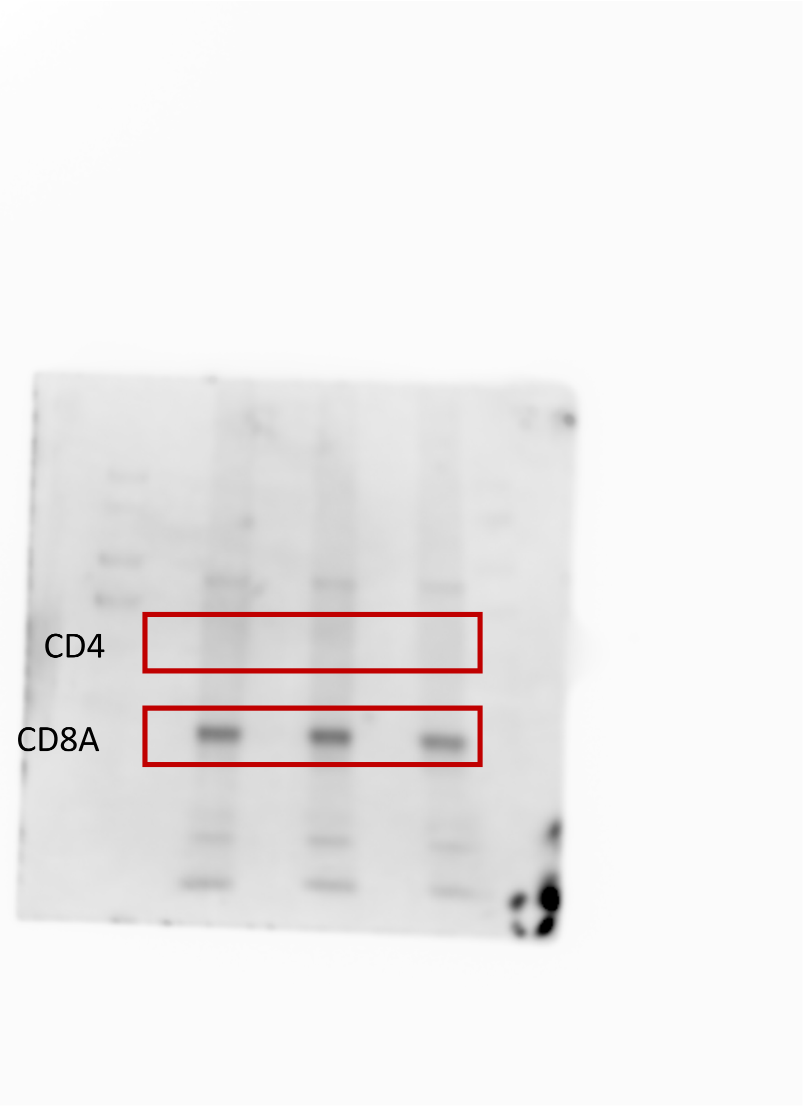

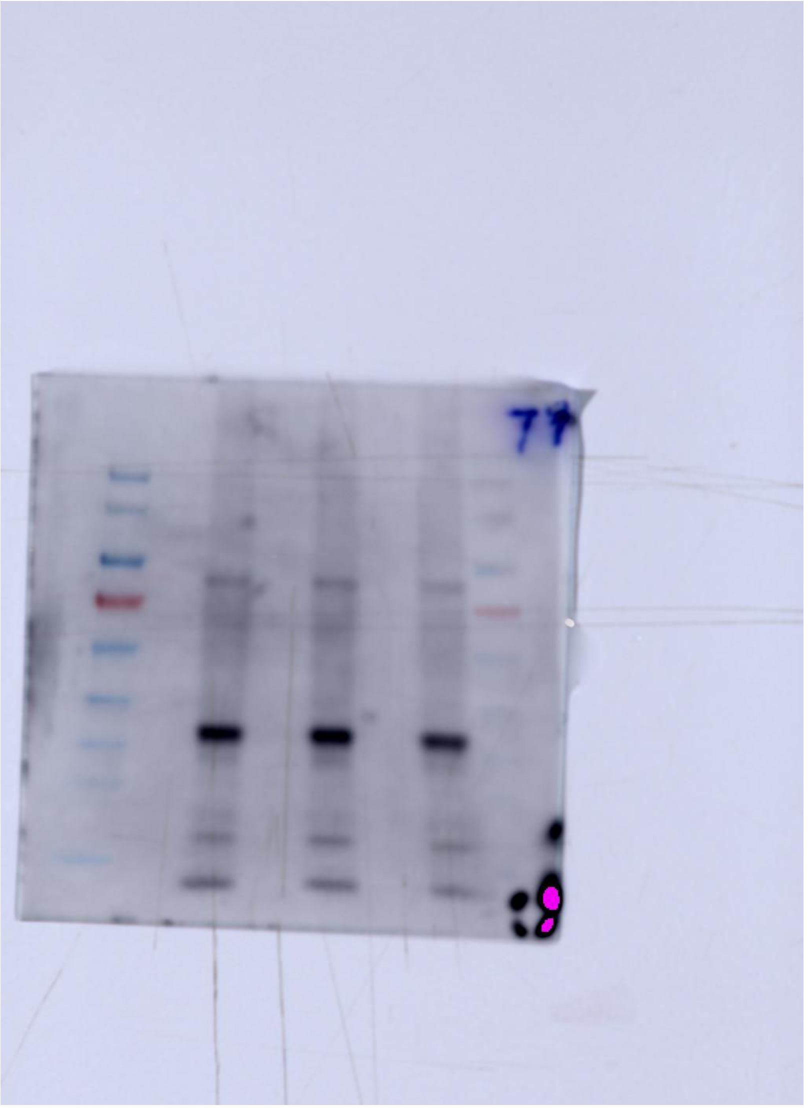

Figure S12D T cells co-cultured with GBC/SD

CXCL13

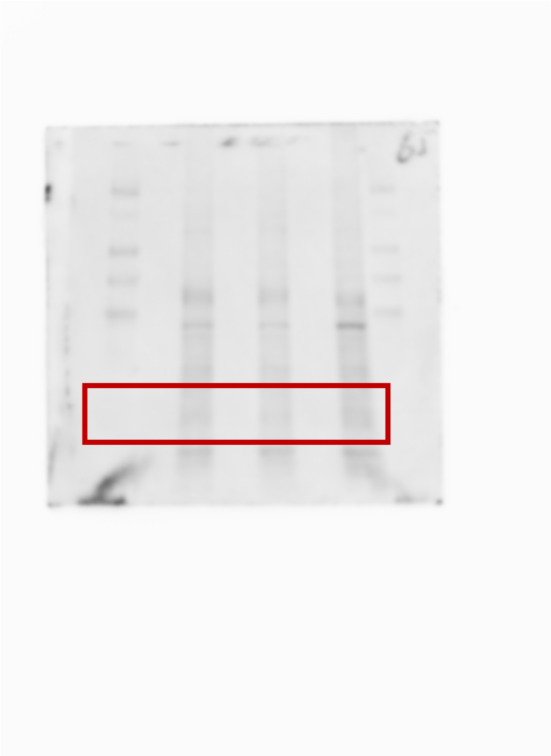

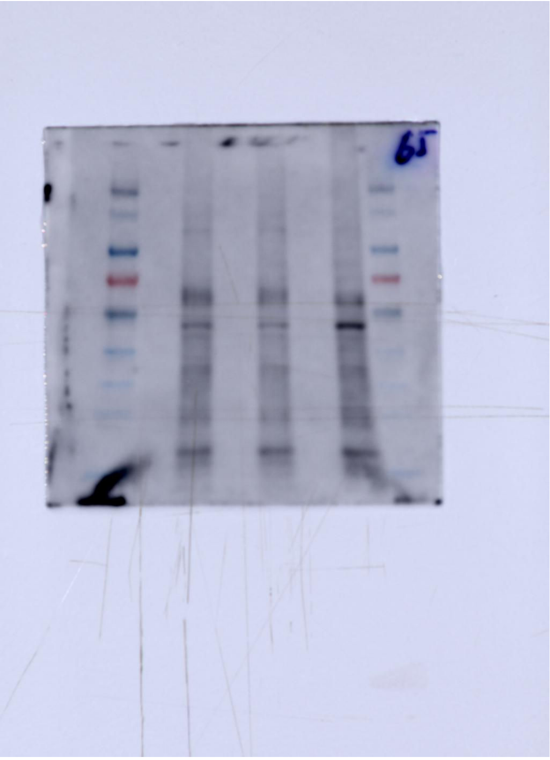

β -Tubulin

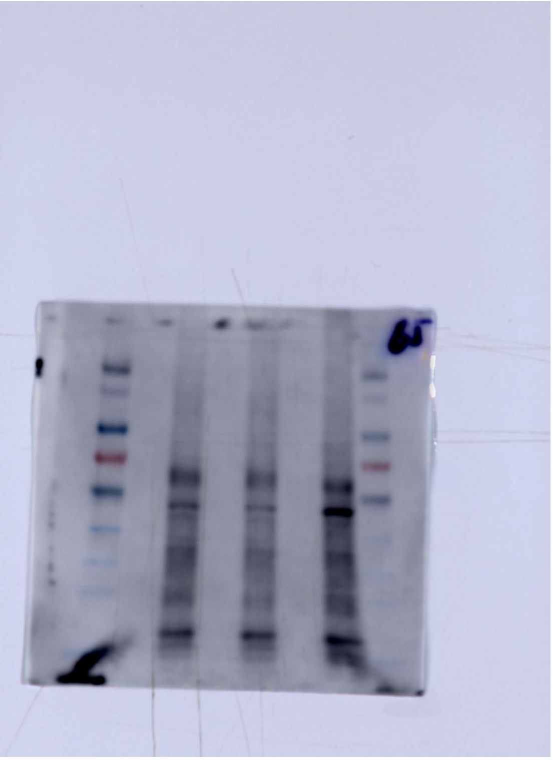

Figure S12D T cells co-cultured with GBC/SD

PD-1

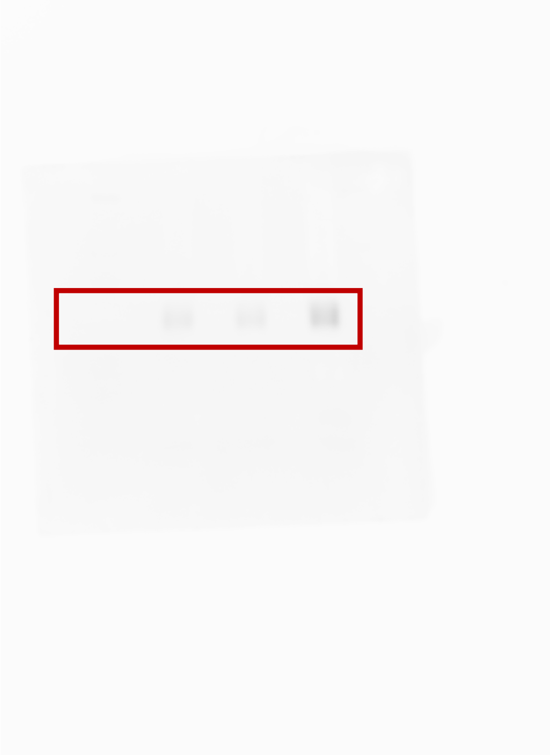

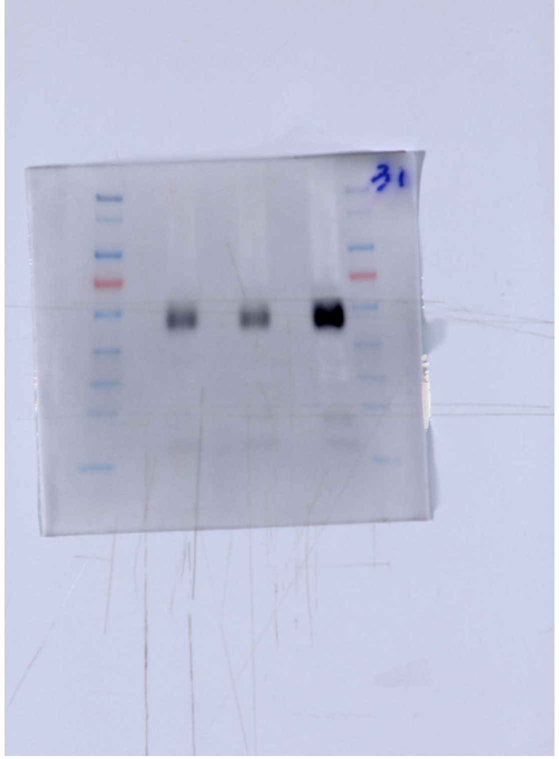

β -Tubulin

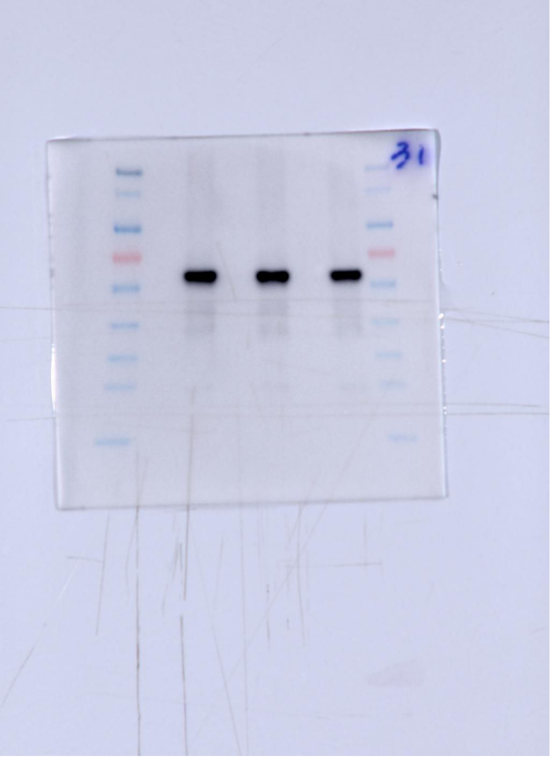

Figure S12D T cells co-cultured with GBC/SD

CTLA-4

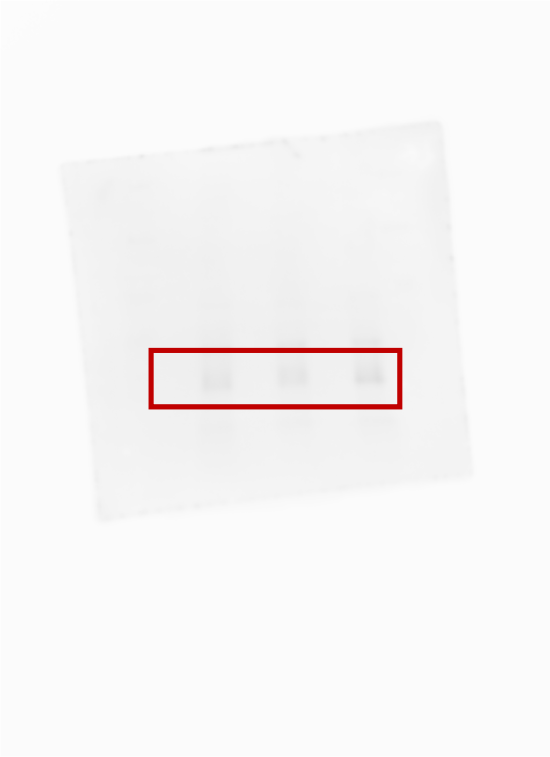

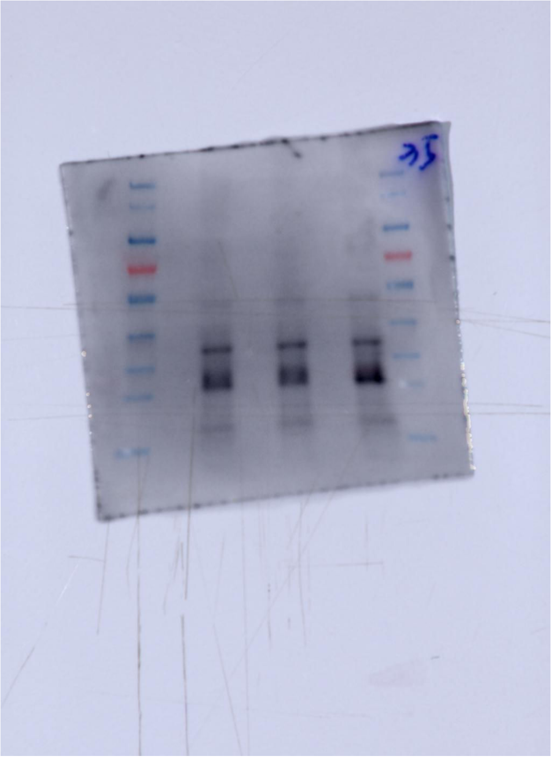

Figure S12D T cells co-cultured with GBC/SD

IFN-γ

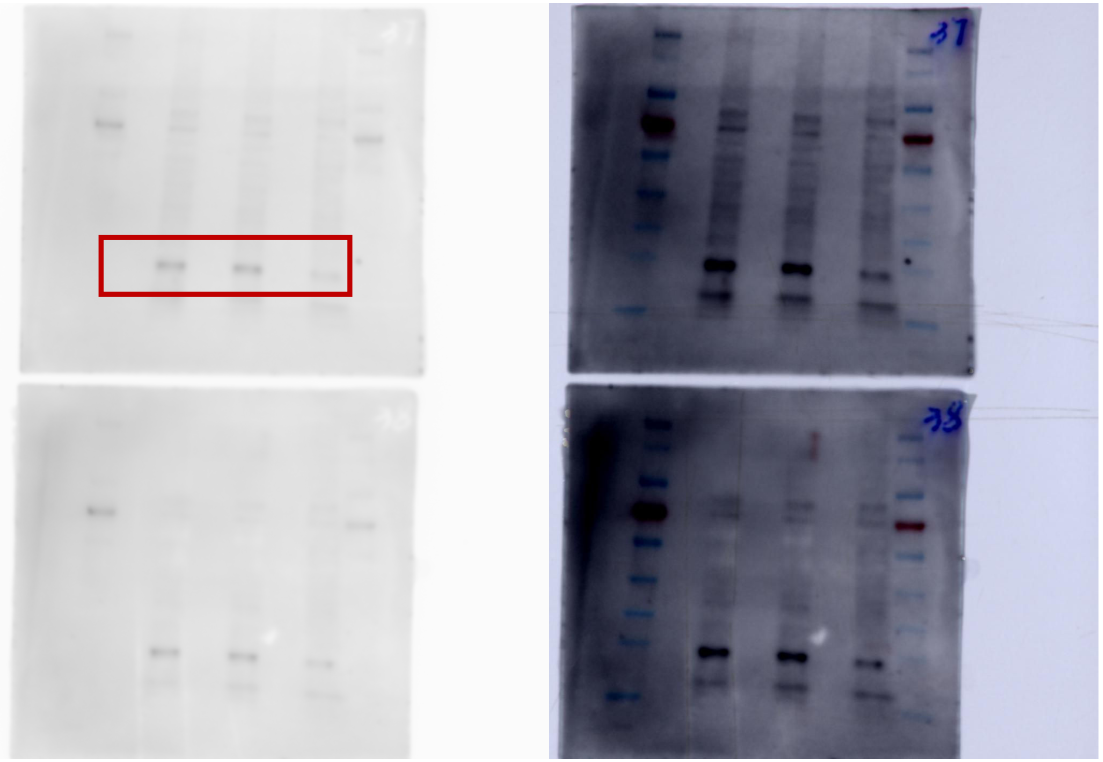

β -Tubulin

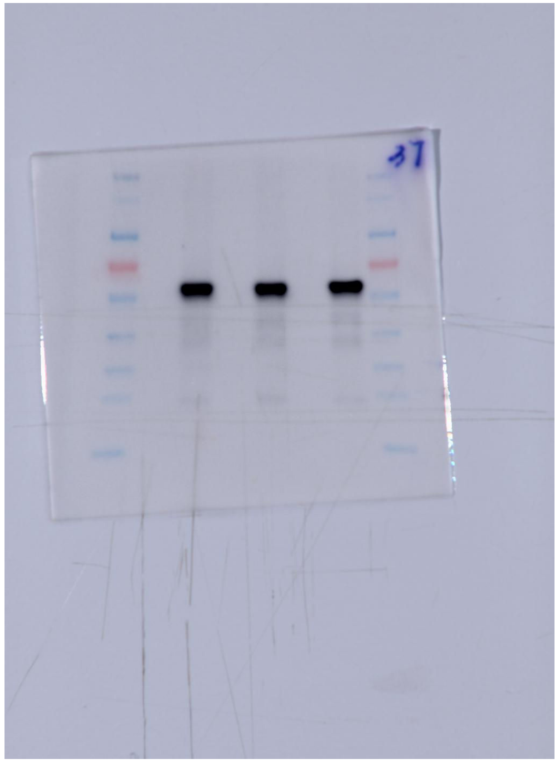

Figure S12D T cells co-cultured with GBC/SD

TNF-α

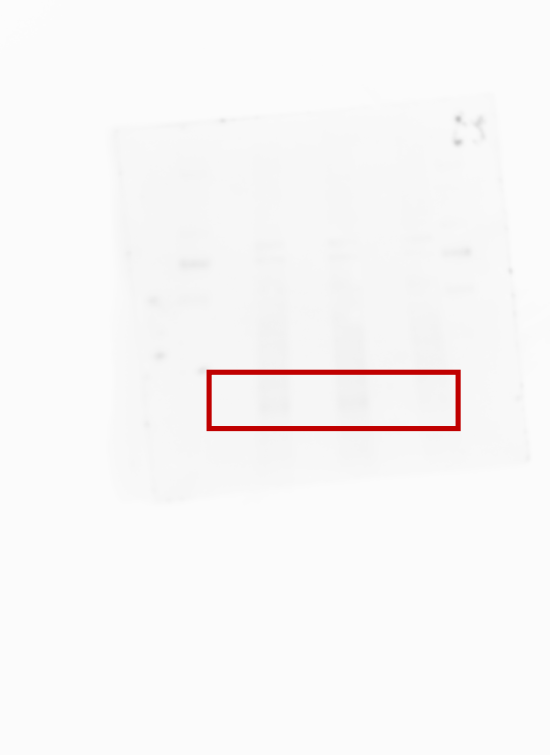

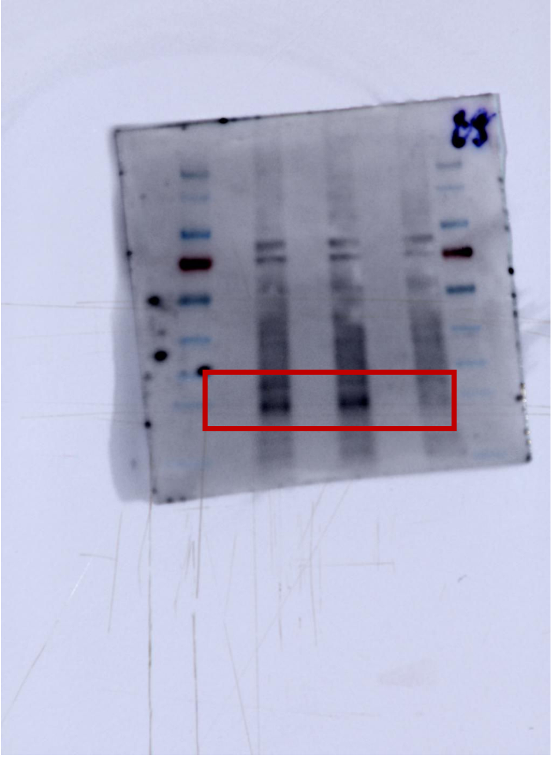

β -Tubulin

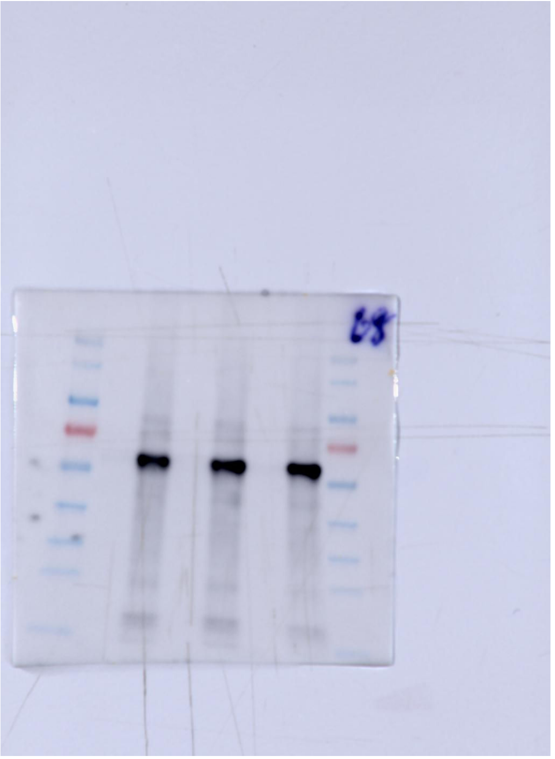

Figure S12D T cells co-cultured with ZJU0430

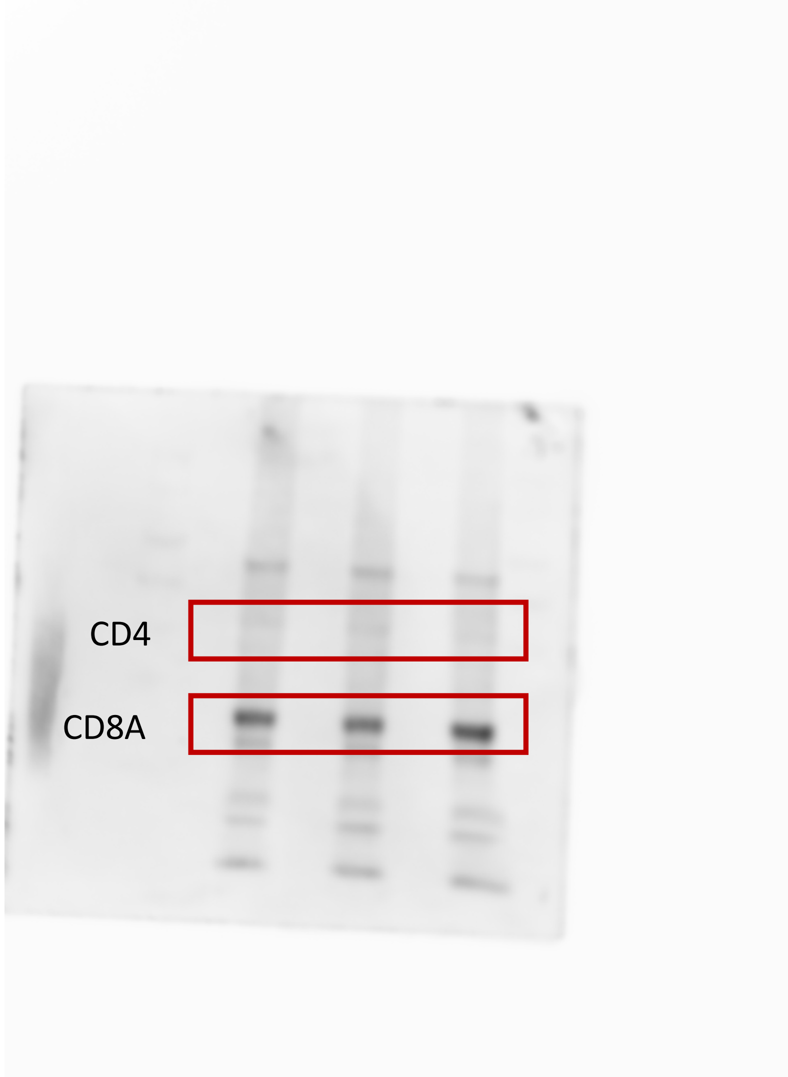

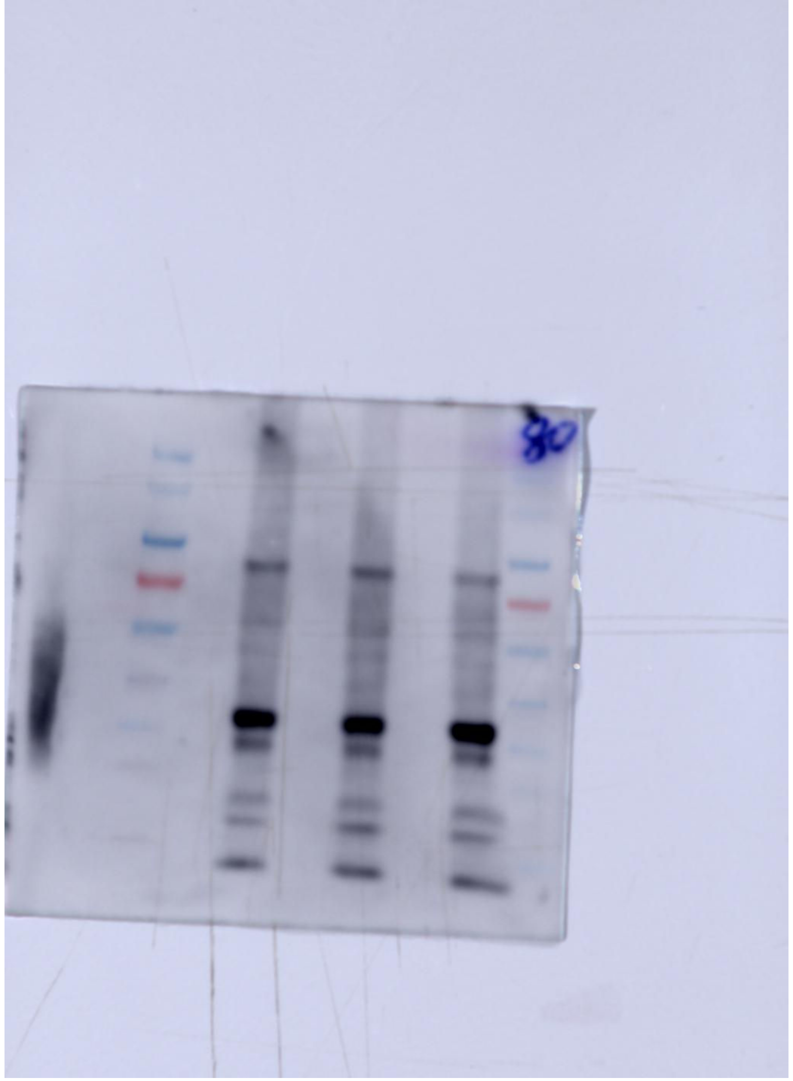

Figure S12D T cells co-cultured with ZJU0430

CXCL13

β -Tubulin

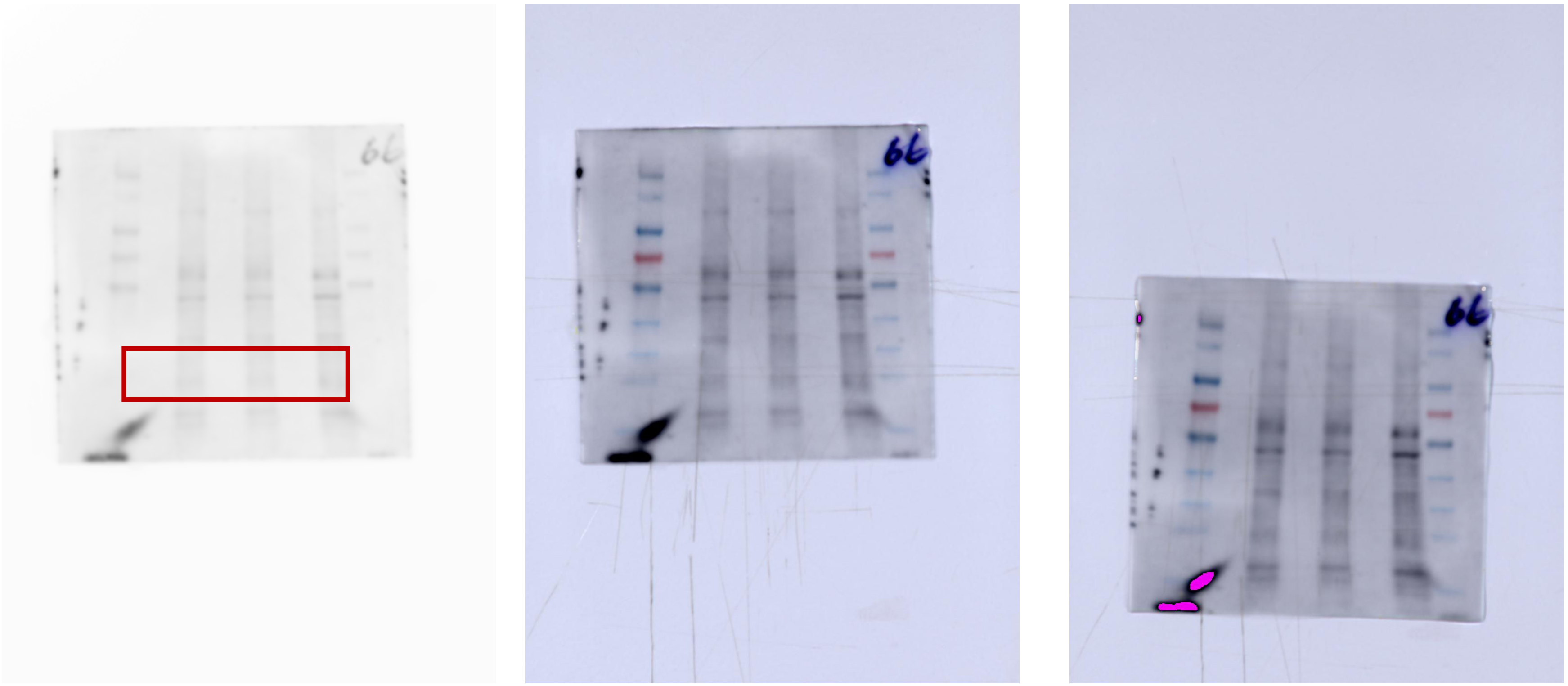

Figure S12D T cells co-cultured with ZJU0430

PD-1

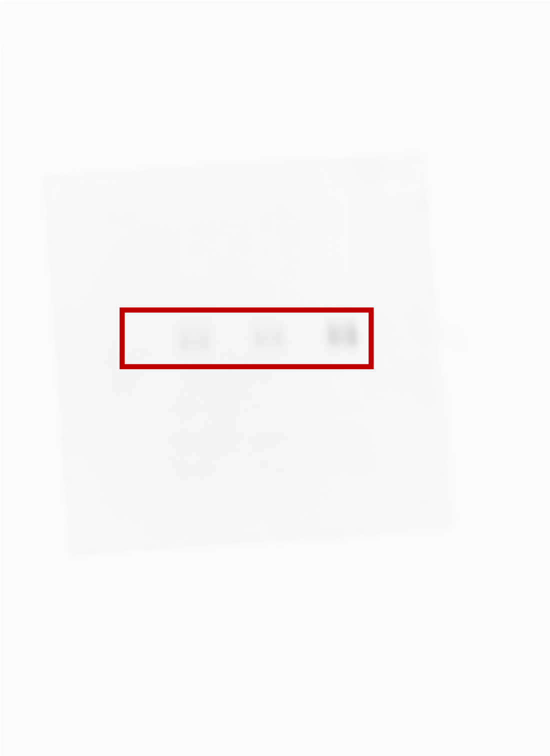

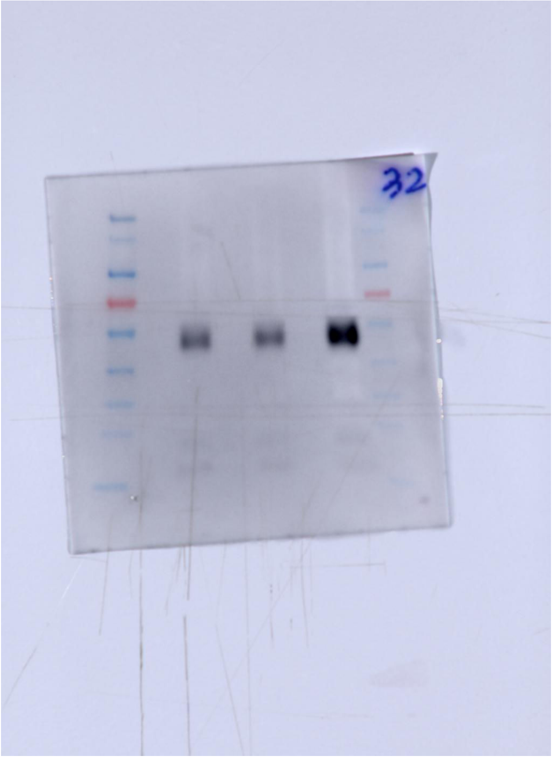

β -Tubulin

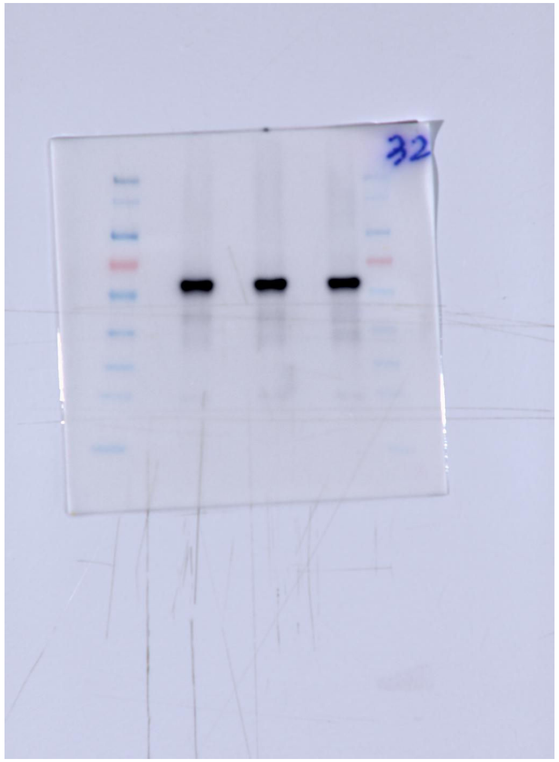

Figure S12D T cells co-cultured with ZJU0430

CTLA-4

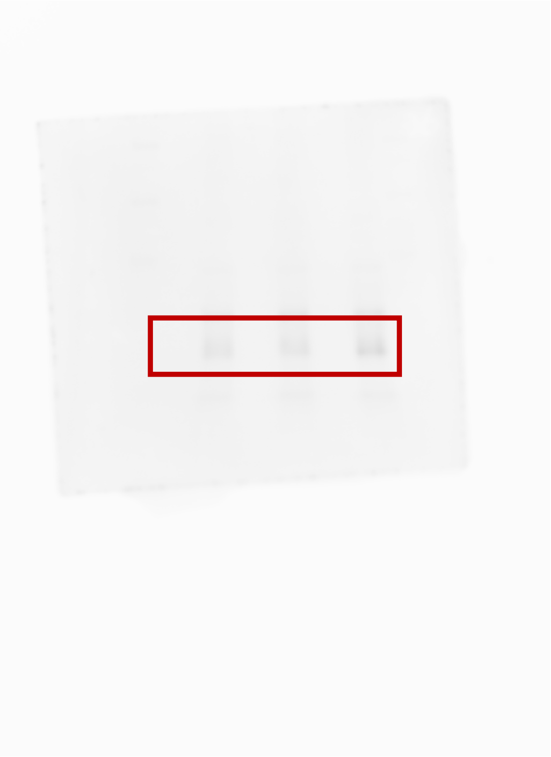

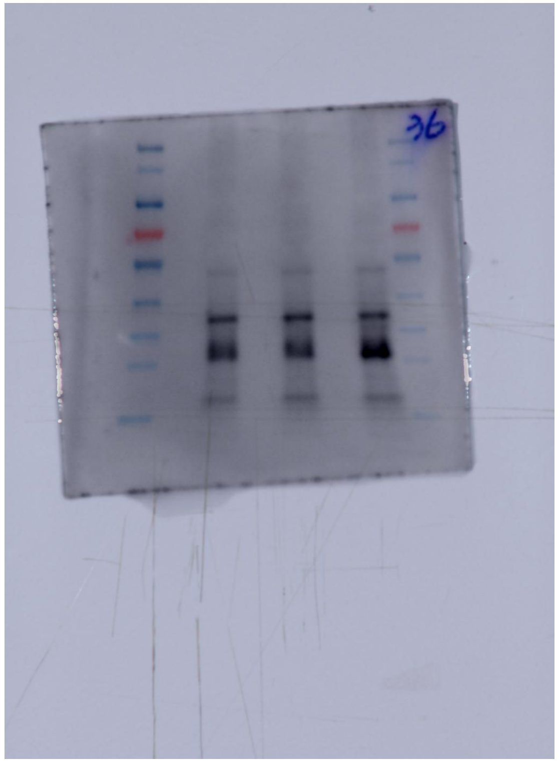

β -Tubulin

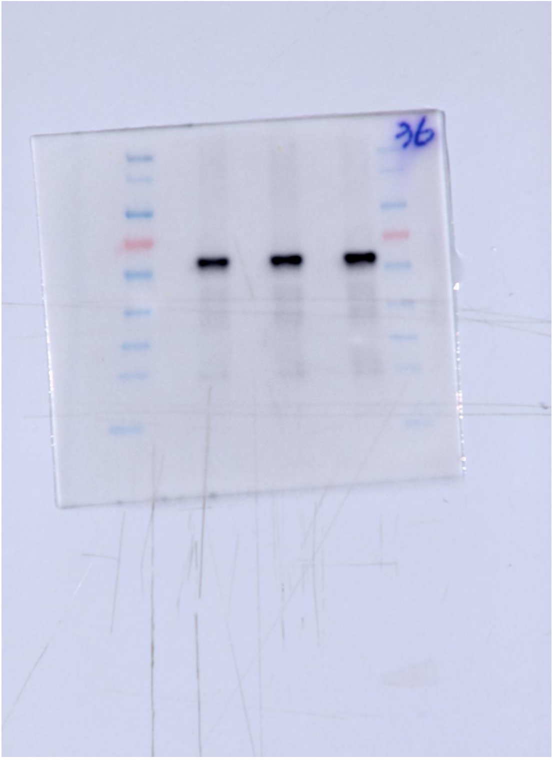

Figure S12D T cells co-cultured with ZJU0430

IFN- γ

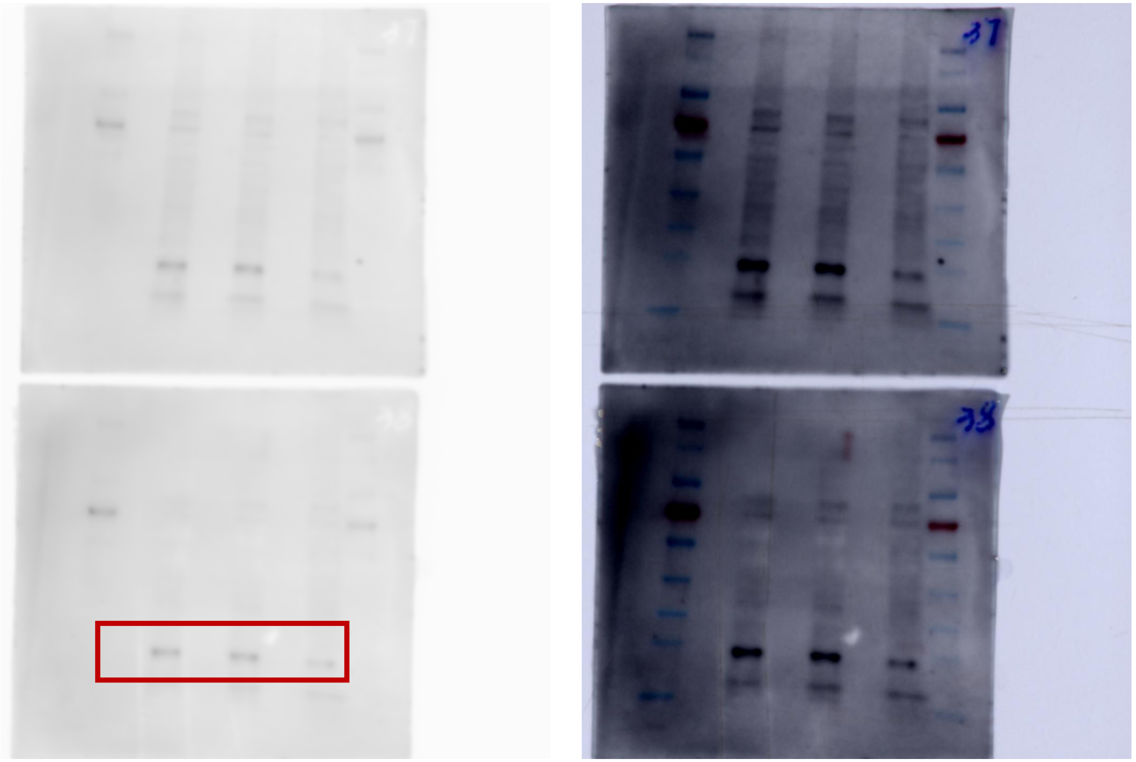

β -Tubulin

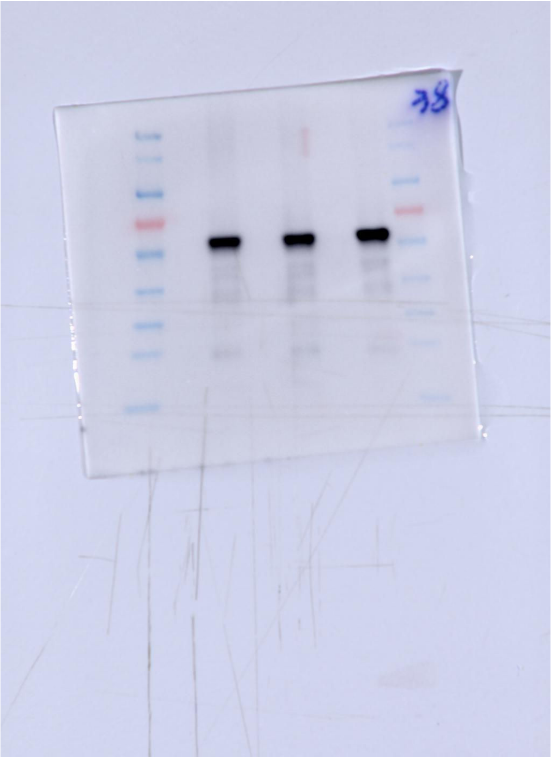

Figure S12D T cells co-cultured with ZJU0430

TNF-α

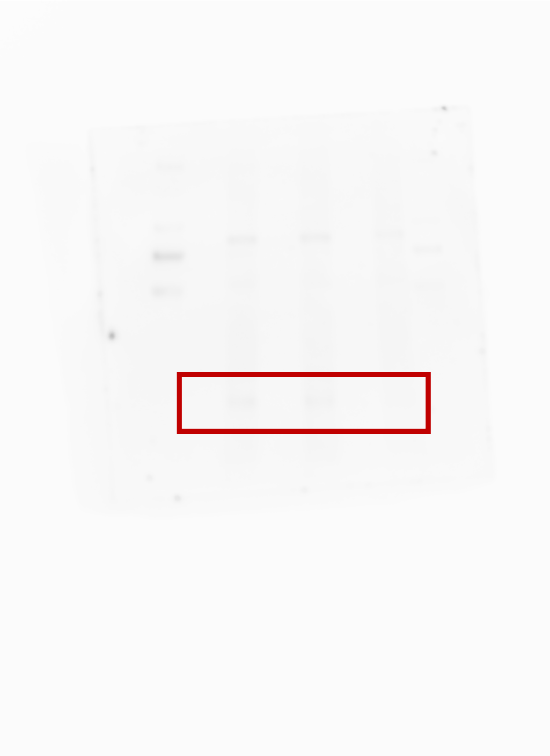

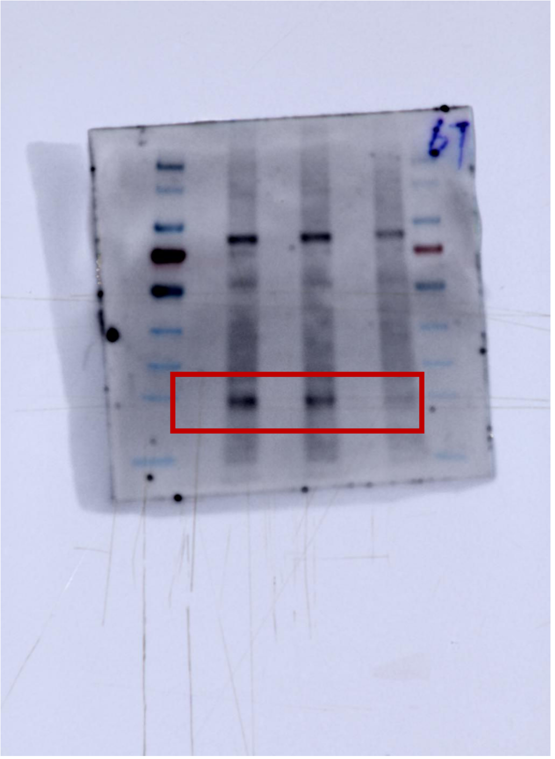

β -Tubulin

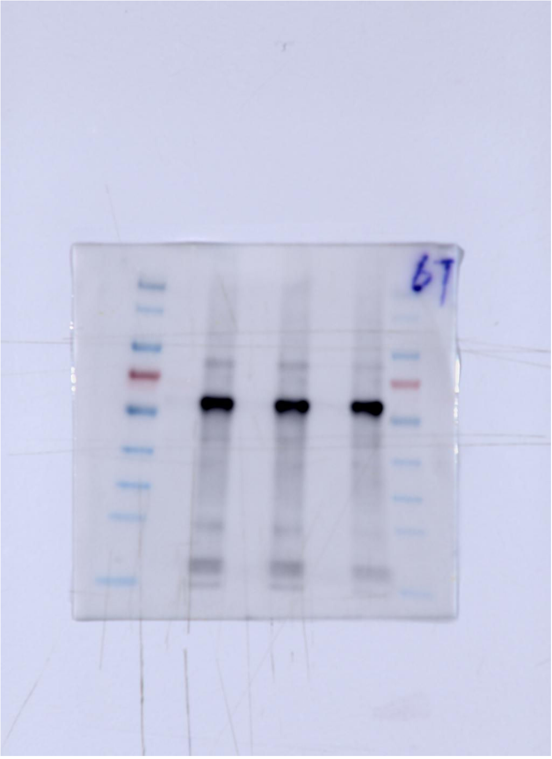

Figure S12E T cells co-cultured with GBC/SD

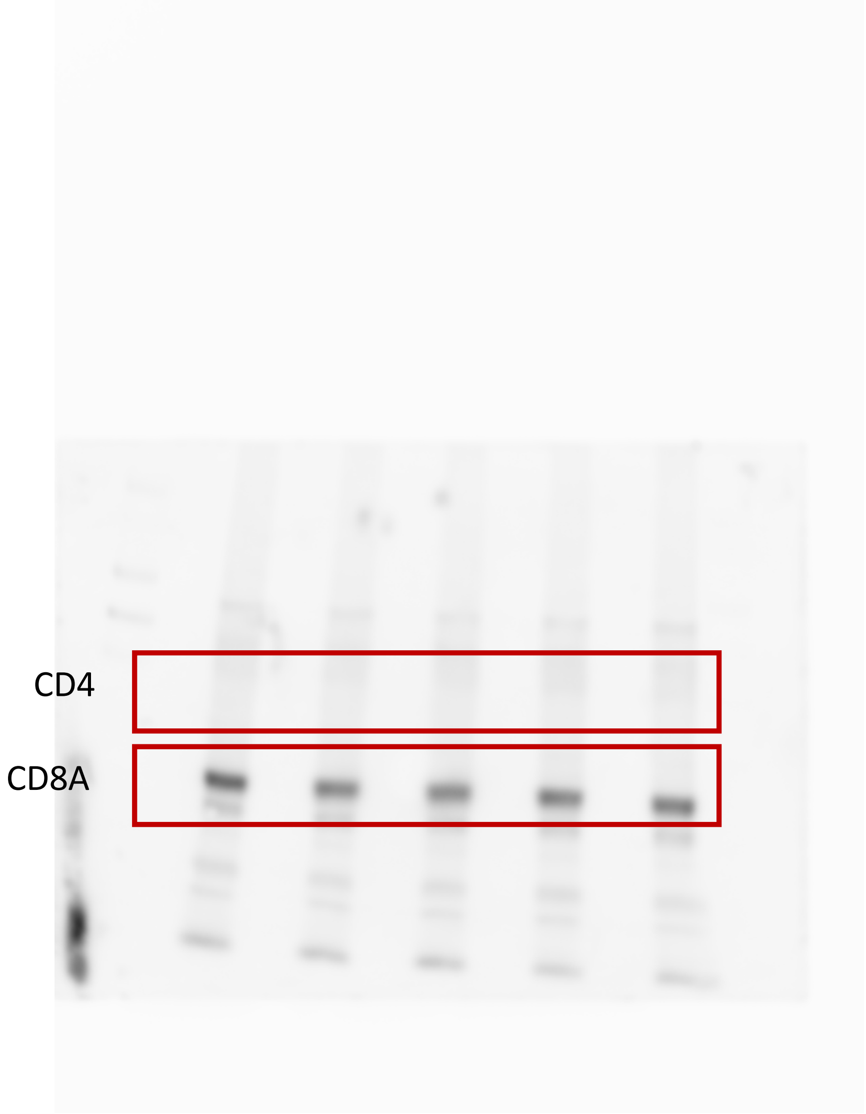

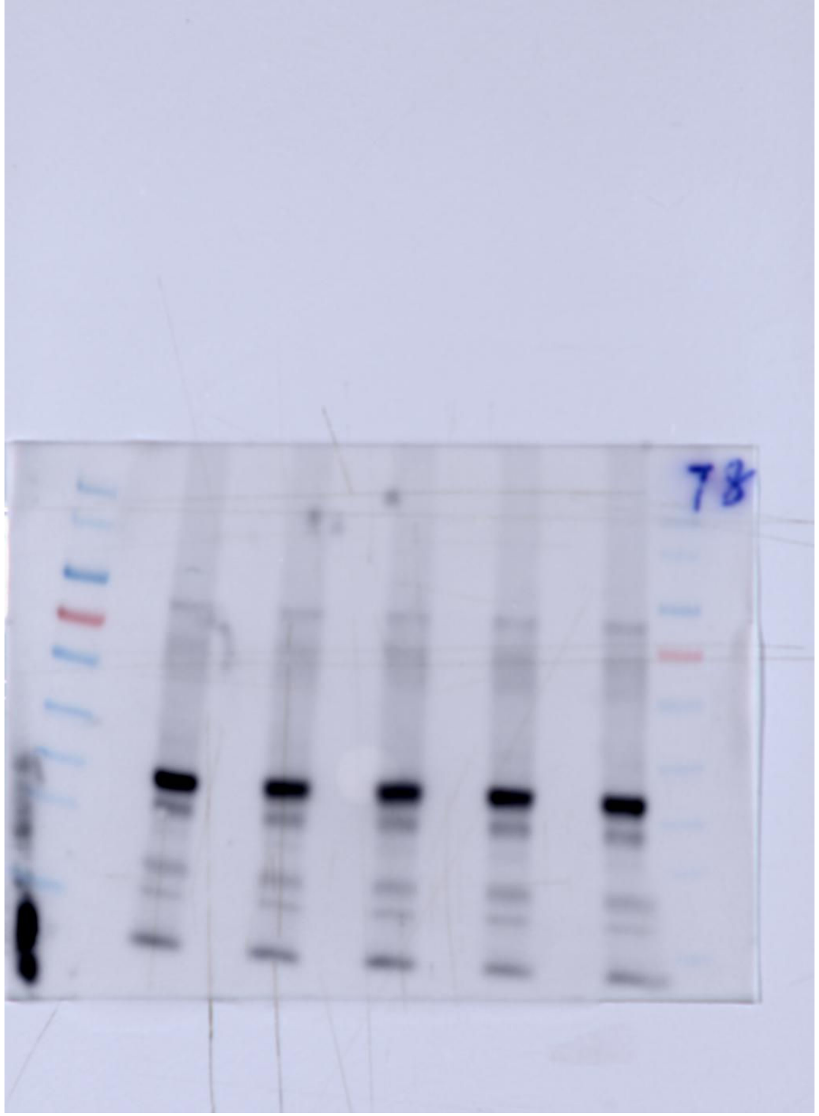

Figure S12E T cells co-cultured with GBC/SD

CXCL13

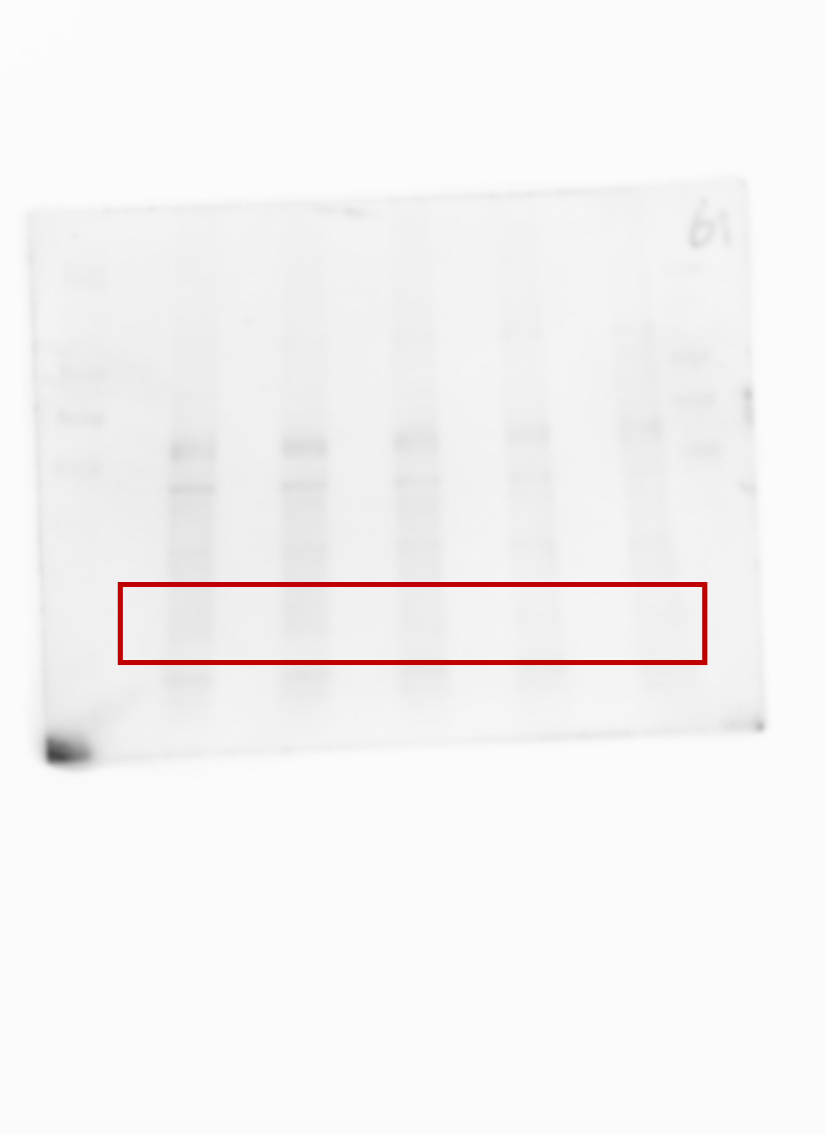

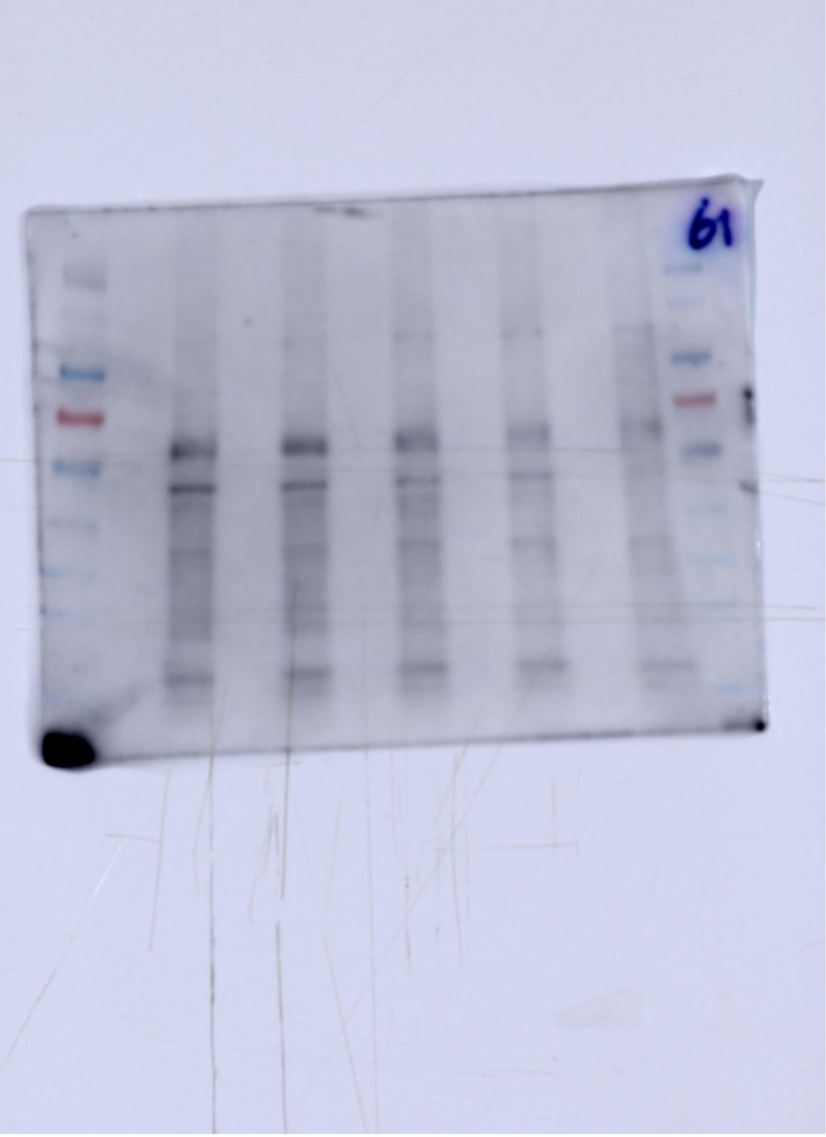

Figure S12E T cells co-cultured with GBC/SD

PD-1

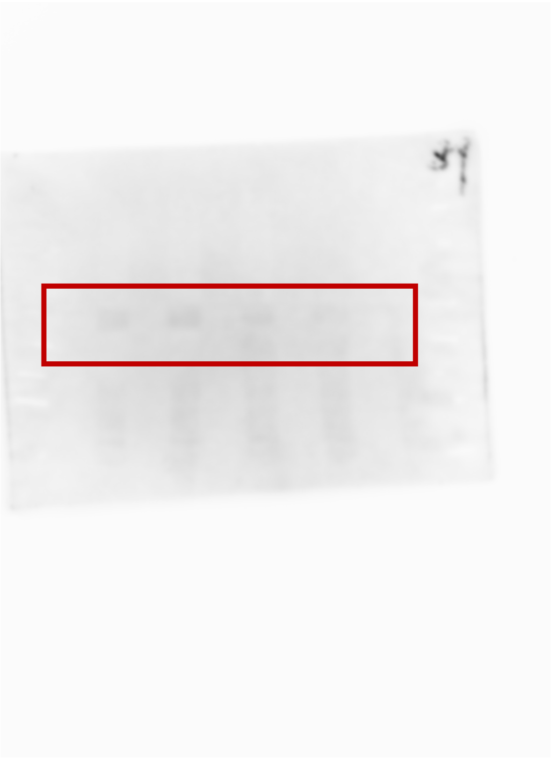

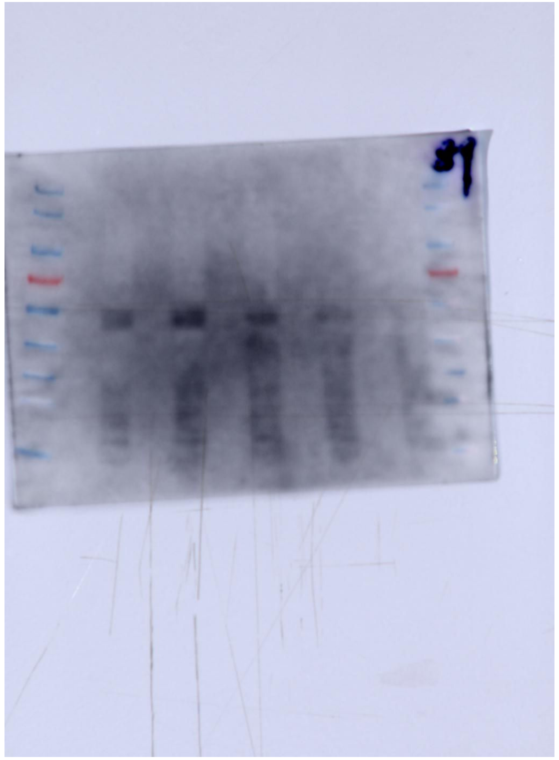

β -Tubulin

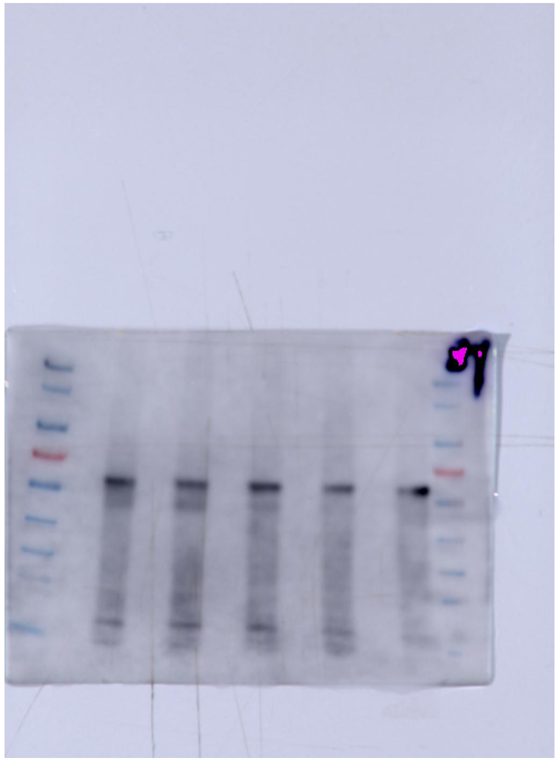

Figure S12E T cells co-cultured with GBC/SD

CTLA-4

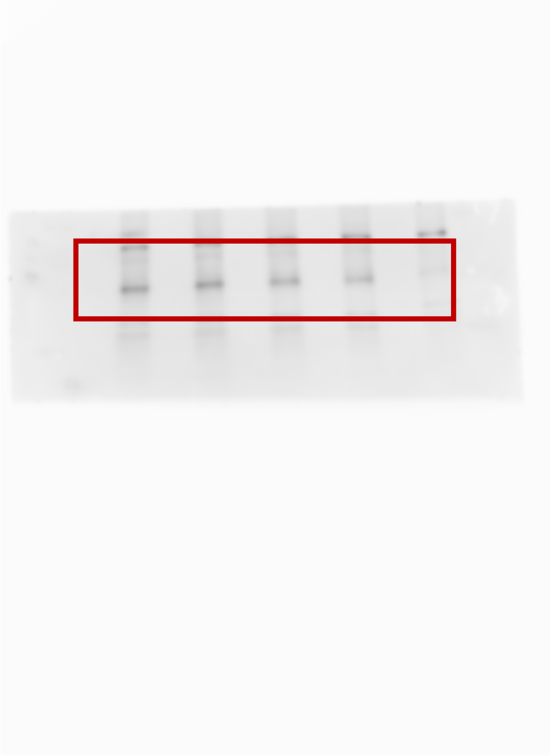

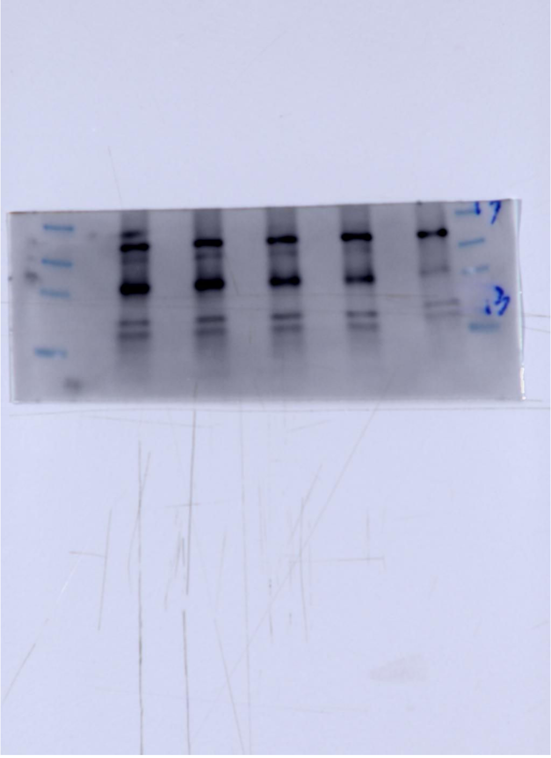

β -Tubulin

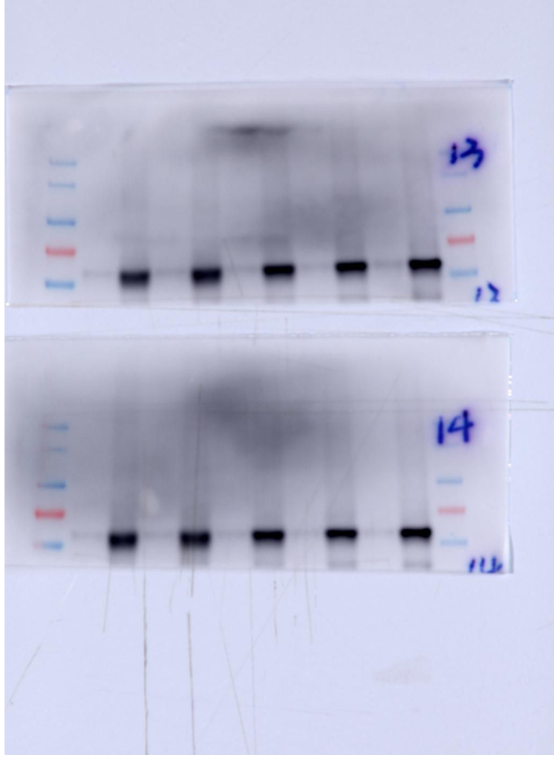

Figure S12E T cells co-cultured with GBC/SD

IFN- γ

β -Tubulin

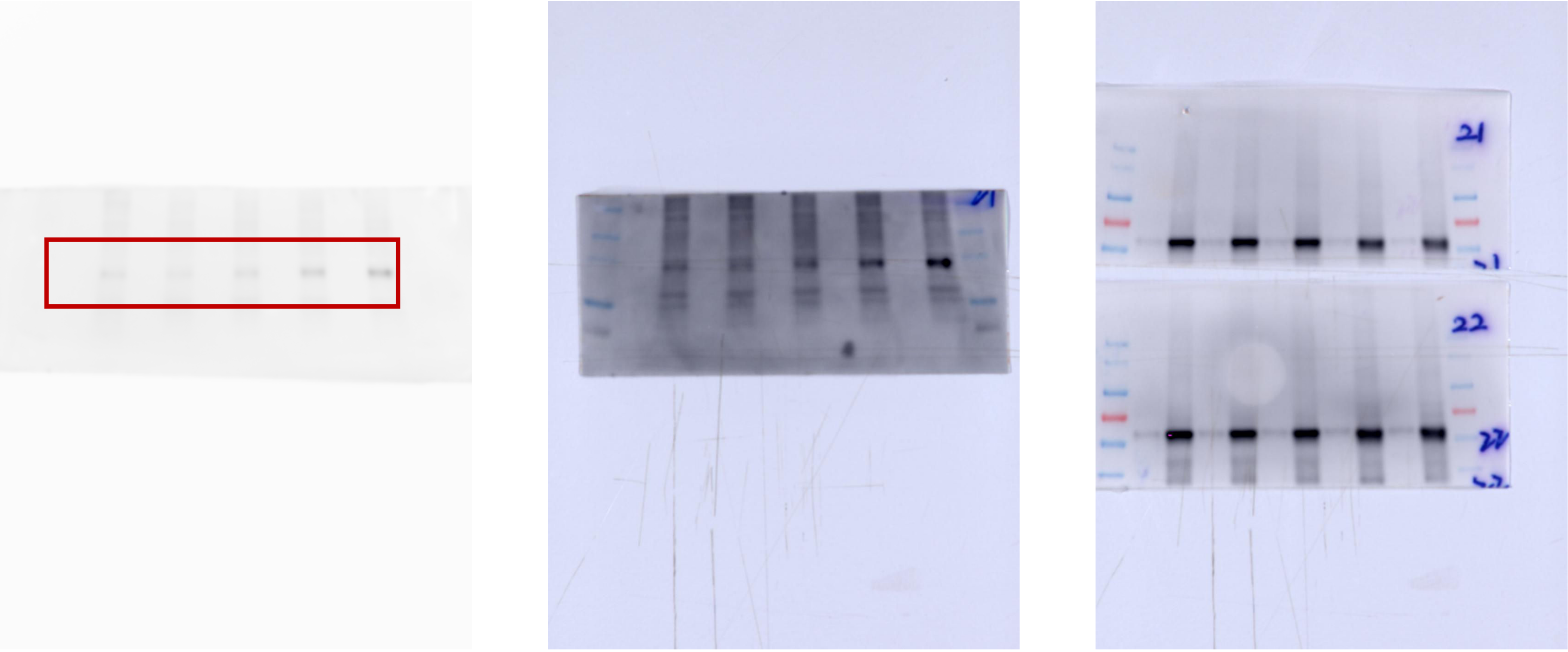

Figure S12E T cells co-cultured with GBC/SD

TNF-α

β -Tubulin

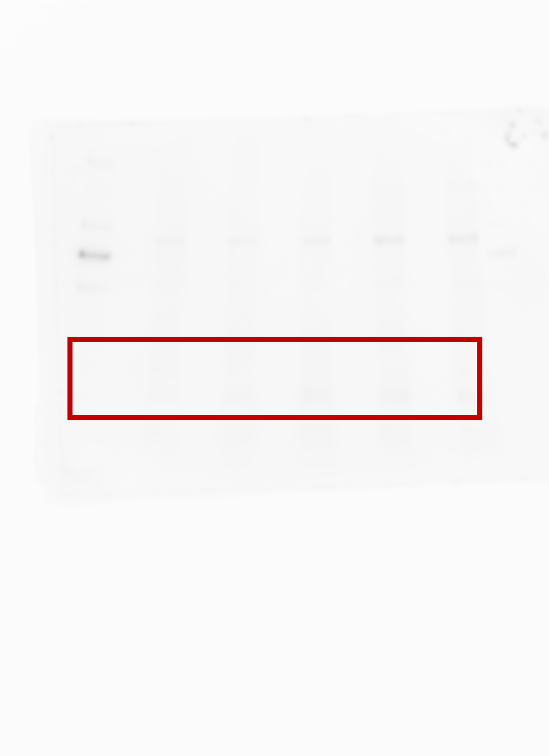

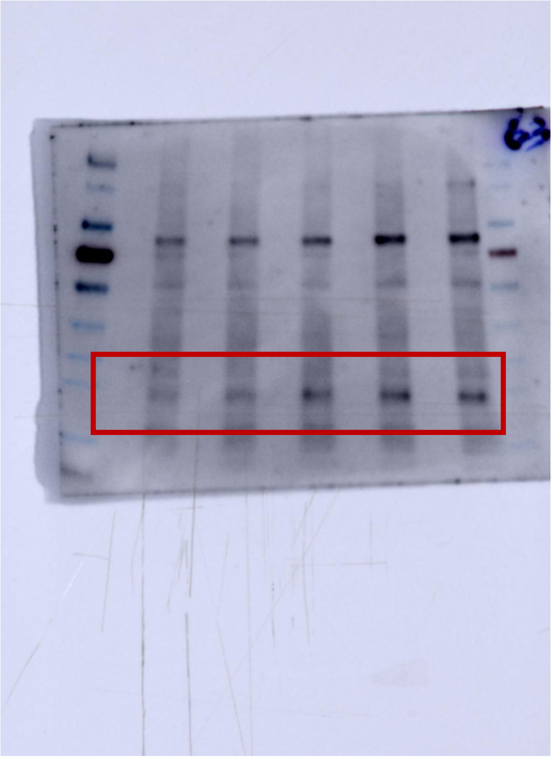

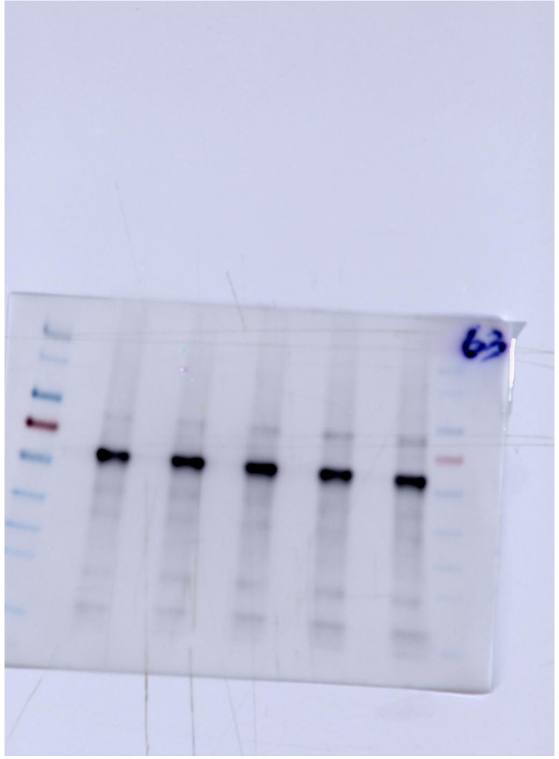

Figure S12E T cells co-cultured with ZJU0430

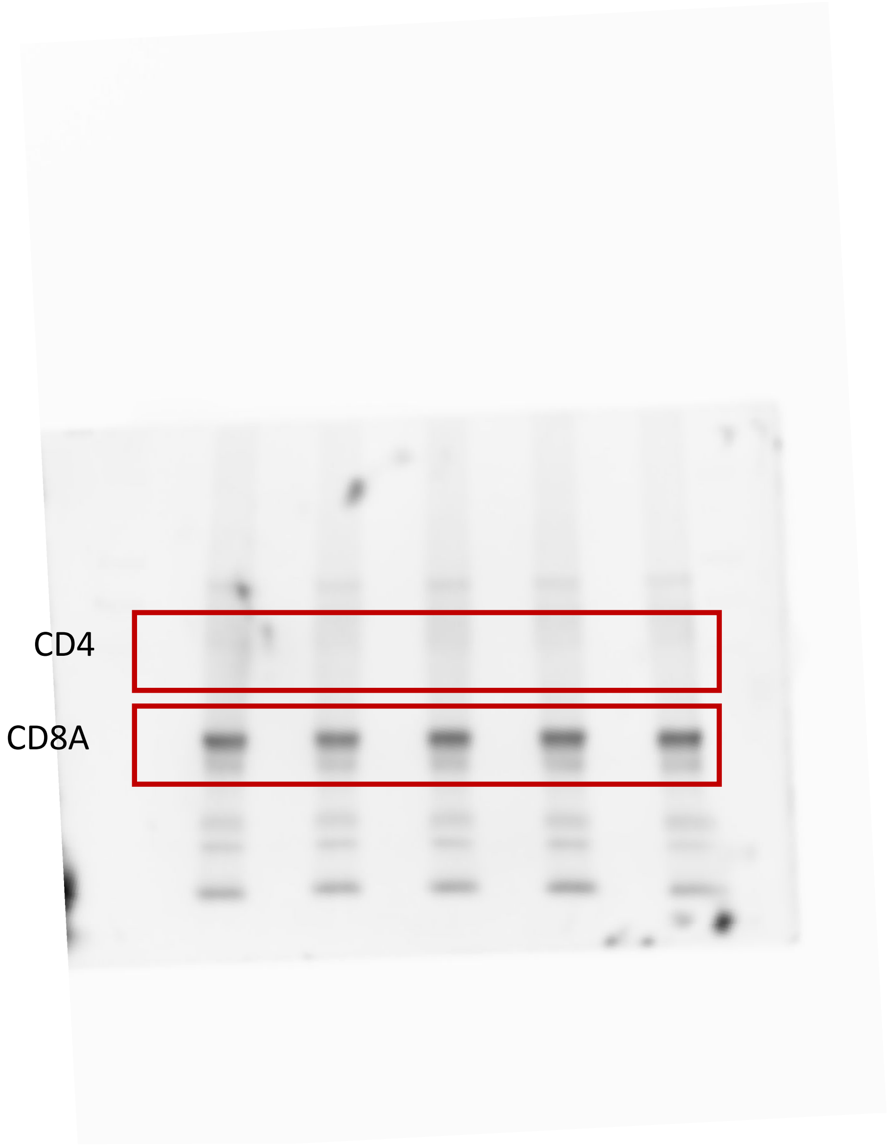

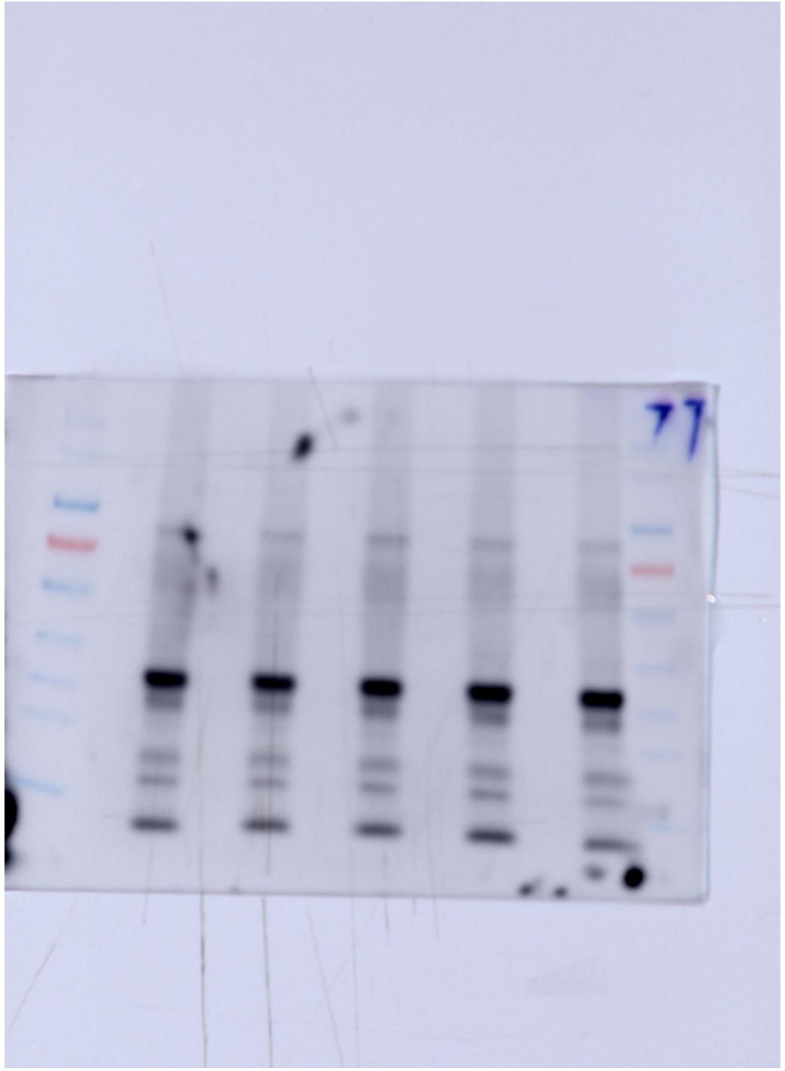

Figure S12E T cells co-cultured with ZJU0430

CXCL13

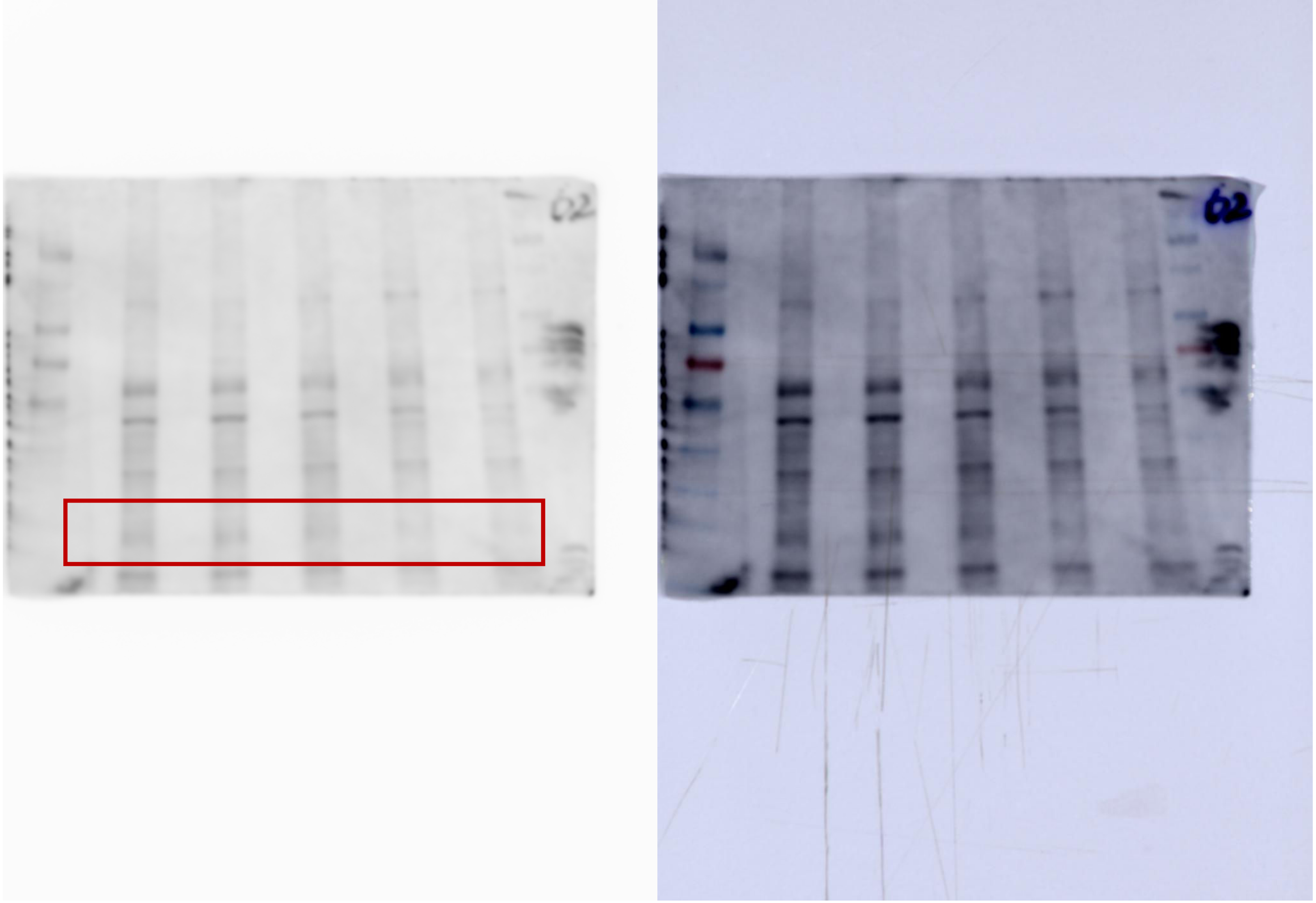

Figure S12E T cells co-cultured with ZJU0430

PD-1

β -Tubulin

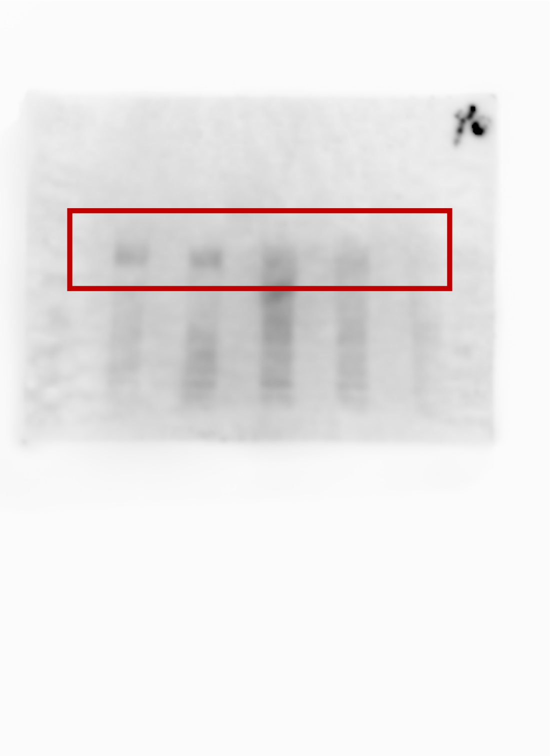

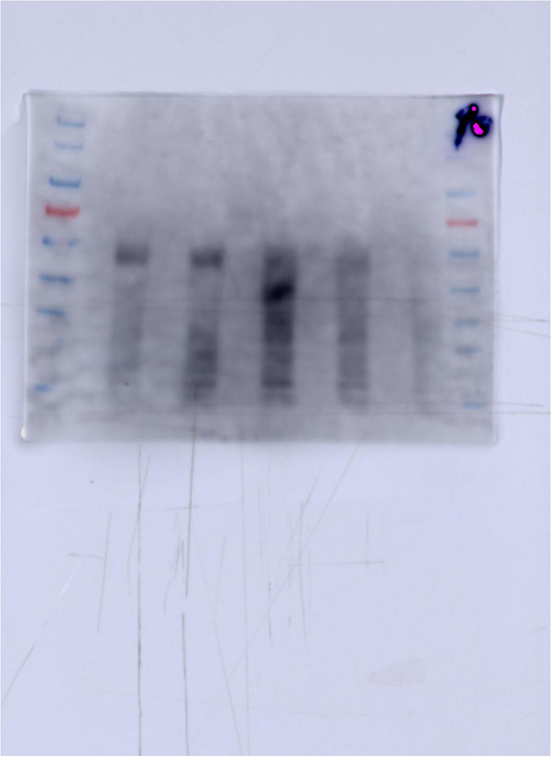

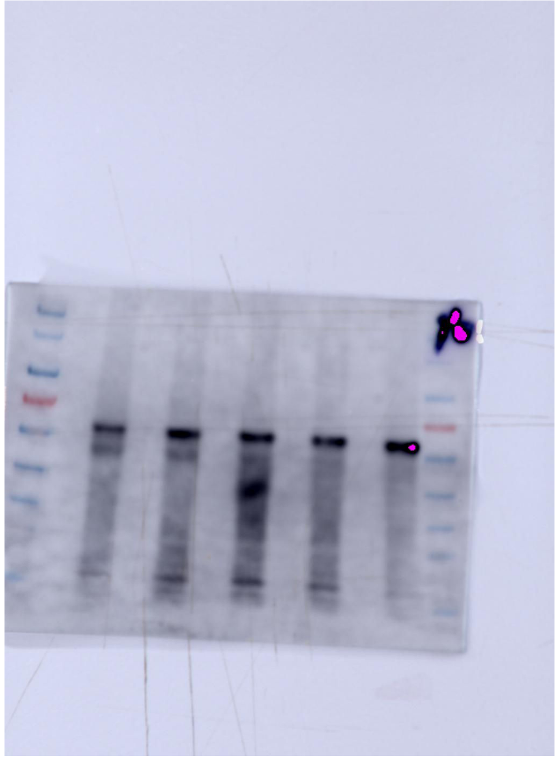

Figure S12E T cells co-cultured with ZJU0430

CTLA-4

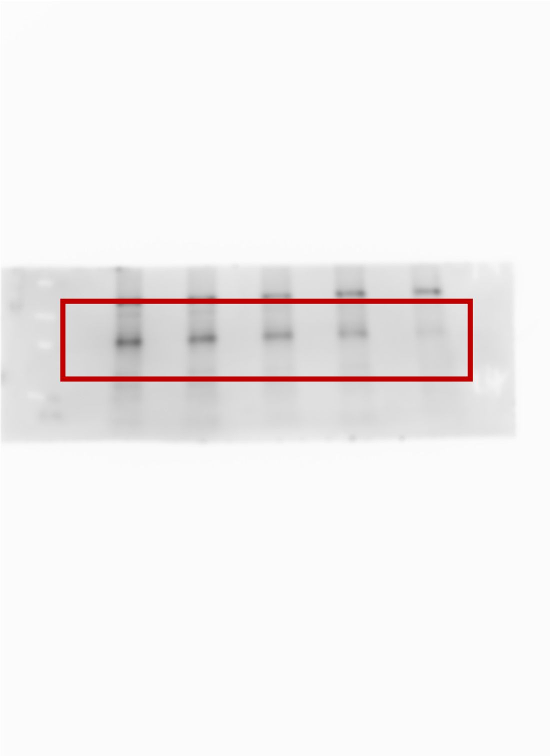

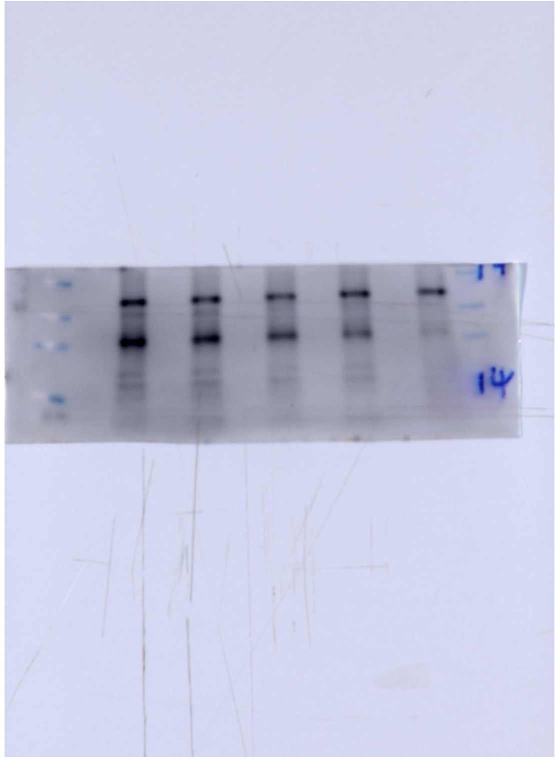

β -Tubulin

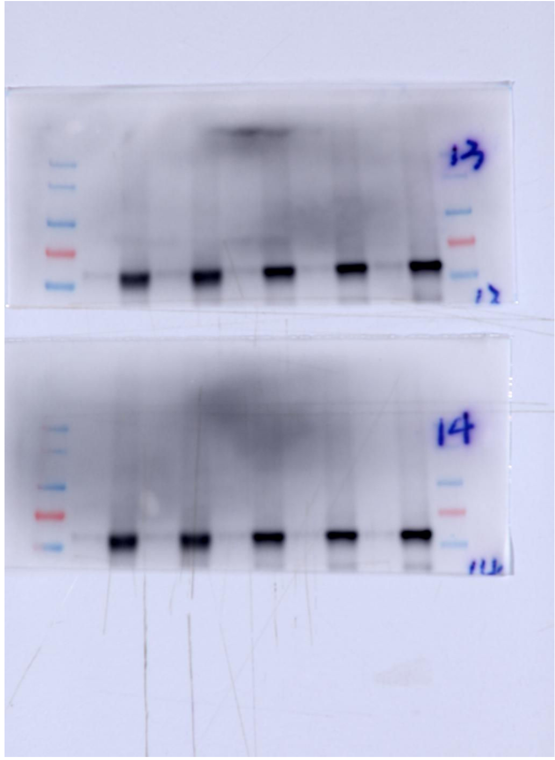

Figure S12E T cells co-cultured with ZJU0430

IFN- γ

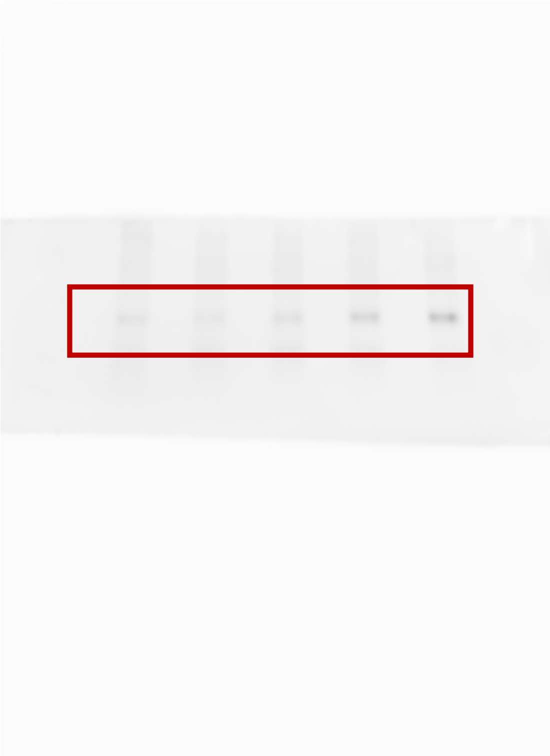

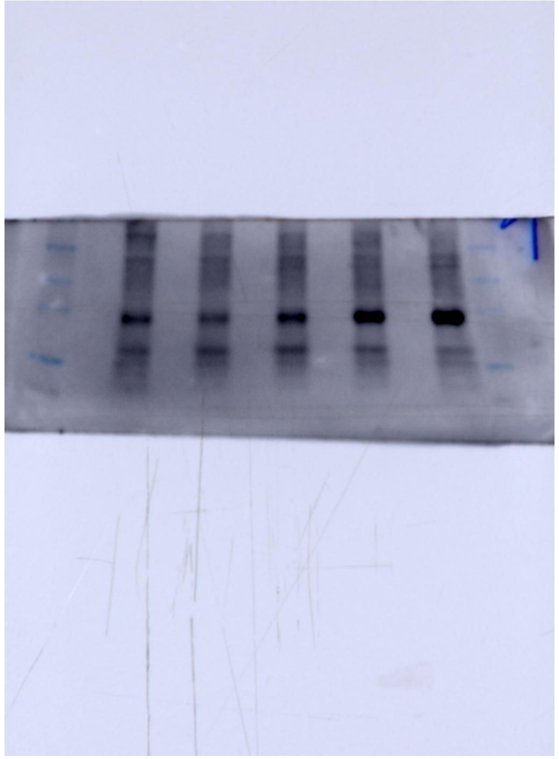

β -Tubulin

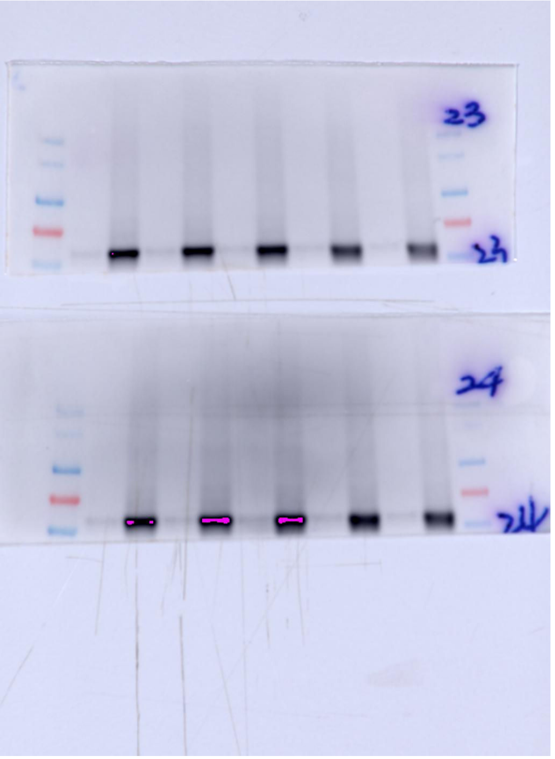

Figure S12E T cells co-cultured with ZJU0430

TNF-α

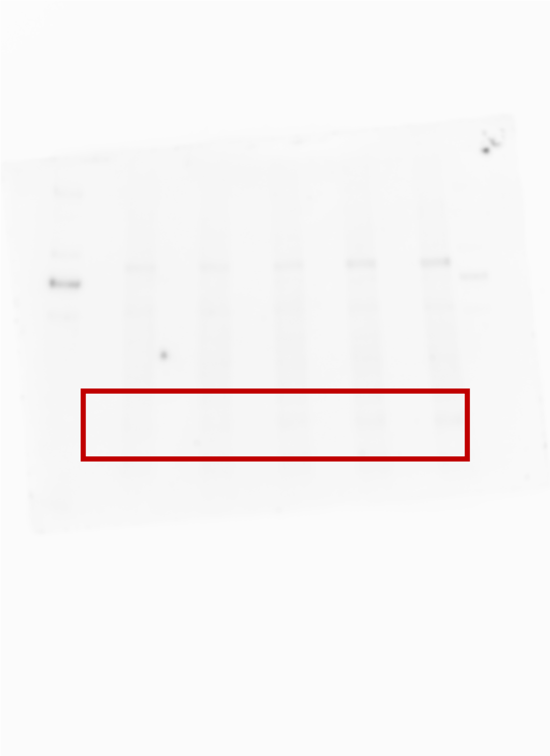

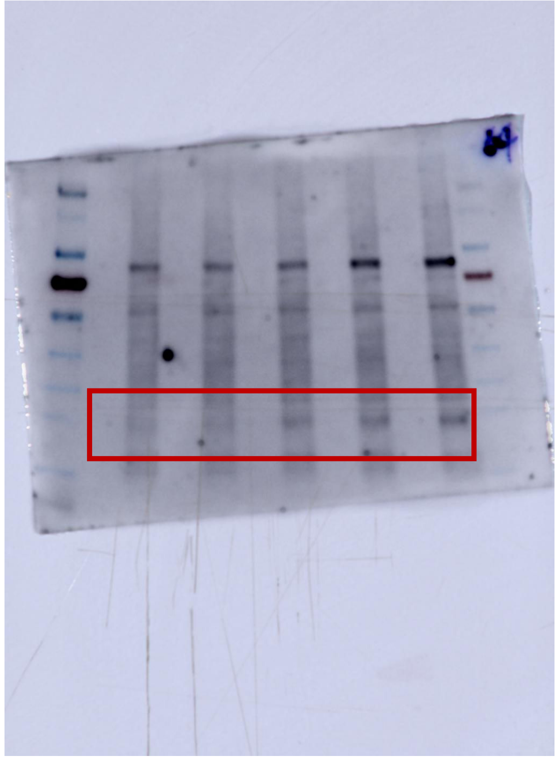

β -Tubulin

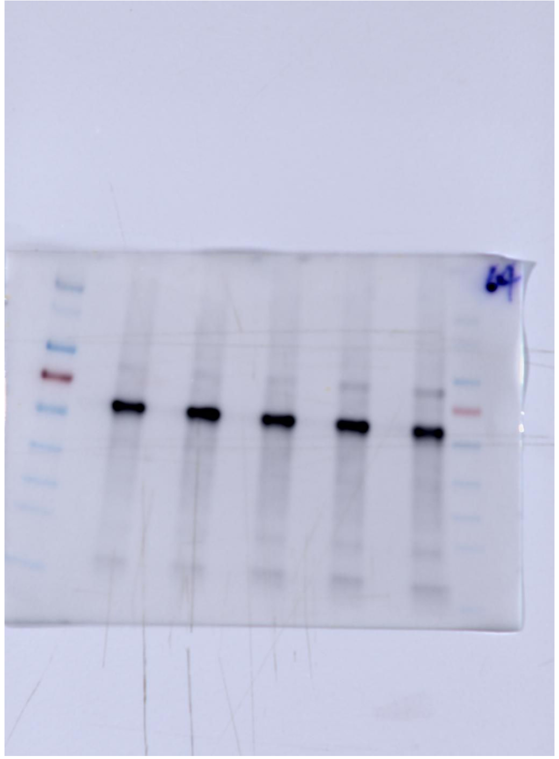

Supplement: Unedited blot and gel images [file jci-136-193672-s035.pdf]
